# Hemithioindigo‐Based Visible Light‐Activated Molecular Machines Kill Bacteria by Oxidative Damage

**DOI:** 10.1002/advs.202203242

**Published:** 2022-08-24

**Authors:** Ana L. Santos, Alexis van Venrooy, Anna K. Reed, Aaron M. Wyderka, Víctor García‐López, Lawrence B. Alemany, Antonio Oliver, George P. Tegos, James M. Tour

**Affiliations:** ^1^ Department of Chemistry Rice University Houston TX 77005 USA; ^2^ IdISBA – Fundación de Investigación Sanitaria de las Islas Baleares Palma 07120 Spain; ^3^ Shared Equipment Authority Rice University Houston TX 77005 USA; ^4^ Servicio de Microbiologia Hospital Universitari Son Espases Palma 07120 Spain; ^5^ Office of Research Reading Hospital Tower Health 420 S. Fifth Avenue West Reading PA 19611 USA; ^6^ Smalley‐Curl Institute Rice University Houston TX 77005 USA; ^7^ Department of Materials Science and Nanoengineering Rice University Houston TX 77005 USA; ^8^ NanoCarbon Center and the Welch Institute for Advanced Materials Rice University Houston TX 77005 USA

**Keywords:** antibiotic resistance, Gram‐positive bacteria, hemithioindigo, molecular switches and motors, visible light active

## Abstract

Antibiotic resistance is a growing health threat. There is an urgent and critical need to develop new antimicrobial modalities and therapies. Here, a set of hemithioindigo (HTI)‐based molecular machines capable of specifically killing Gram‐positive bacteria within minutes of activation with visible light (455 nm at 65 mW cm^−2^) that are safe for mammalian cells is described. Importantly, repeated exposure of bacteria to HTI does not result in detectable development of resistance. Visible light‐activated HTI kill both exponentially growing bacterial cells and antibiotic‐tolerant persister cells of various Gram‐positive strains, including methicillin‐resistant *S. aureus* (MRSA). Visible light‐activated HTI also eliminate biofilms of *S. aureus* and *B. subtilis* in as little as 1 h after light activation. Quantification of reactive oxygen species (ROS) formation and protein carbonyls, as well as assays with various ROS scavengers, identifies oxidative damage as the underlying mechanism for the antibacterial activity of HTI. In addition to their direct antibacterial properties, HTI synergize with conventional antibiotics in vitro and in vivo, reducing the bacterial load and mortality associated with MRSA infection in an invertebrate burn wound model. To the best of the authors’ knowledge, this is the first report on the antimicrobial activity of HTI‐based molecular machines.

## Graphical abstract

Hemithioindigo‐based molecular machines capable of specifically killing Gram‐positive bacteria are synthesized. Killing occurs within minutes of activation of the molecular machines with visible light. Hemithioindigo‐based molecular machines kill methicillin‐resistant *S. aureus*. The molecular machines are safe for mammalian cells. Repeated exposure of bacteria to hemithioindigo‐based molecular machines does not result in detectable resistance development.

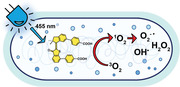

## 1 Introduction

Antimicrobial resistance (AMR) represents one of the most pressing global health threats facing humankind.^[^ ^1^ ^]^ In 2019, AMR was the third leading cause of death worldwide, with bacterial AMR estimated to be directly responsible for 1.27 million deaths, surpassing the mortality rates of malaria and HIV/AIDS.^[^ ^2^ ^]^ By 2050, antimicrobial‐resistant infections could be responsible for more than 10 million deaths per year^[^ ^3^ ^]^ in an impending bacterial pandemic.

Addressing the problem of antibiotic resistance will require not only changing the way antibiotics are used but also developing new antibiotics that have different modes of action from those to which resistance already exists while restoring, maintaining, and/or improving the efficacy of existing antibiotics. However, since the golden age of antibiotic discovery, from the 1940s to the 1960s, resistance to all classes of antibiotics has been recorded, while few new antibiotics have been discovered.^[^ ^4^ ^]^

In recent decades, some advances in antimicrobial research have been made possible by the development of synthetic nanomaterials. Unlike conventional antibiotics, antimicrobial nanomaterials kill bacteria by mechanisms that microorganisms do not typically encounter in nature and, therefore, are not inherently part of their defensive arsenal.^[^ ^5^, ^6^ ^]^ Stimuli‐responsive nanomaterials have the added advantage that their bioactivity can be remotely controlled by external stimuli. Among the stimuli that can be used to activate nanomaterials, light is unmatched in its capacity to control biological systems with high spatial and temporal resolution because it is noninvasive and can be manipulated remotely in an easily reversible manner.^[^ ^7^, ^8^, ^9^ ^]^ The ability to control the release of antimicrobials in space and time can help to minimize the side effects of systemic antibiotics and curb the emergence of resistance while mitigating the long‐term consequences of antimicrobial accumulation in the environment.^[^ ^10^ ^]^

One class of promising stimuli‐activated materials is molecular machines, the discovery of which was awarded the 2016 Nobel Prize in Chemistry.^[^ ^11^ ^]^ One type of synthetic molecular machines can rotate unidirectionally in a controlled manner in response to stimuli, resulting in mechanical action.^[^ ^12^ ^]^ Feringa‐type molecular machines consist of two aryl groups connected by a sterically crowded C=C double bond. One of the aryl groups, the “rotor,” has a chiral carbon atom and rotates relative to the position of the other aryl group, the “stator.” When activated by light, the molecule undergoes two cycles of photoisomerization, followed by thermal helical inversion, resulting in a 360° unidirectional rotation. This “drill‐like” motion can be used for various purposes, such as opening holes in cell membranes through which drugs can be delivered.^[^ ^13^ ^]^ There are other types of molecular machines that “switch” between the *E* and *Z* conformations as opposed to the 360° rotation of the Feringa‐type molecules.^[^ ^13^, ^14^, ^15^ ^]^

Molecular machines show great promise in various technological and medical applications, from drug delivery to antimicrobial therapy.^[^ ^16^, ^17^ ^]^ Feringa‐type molecular machines have been shown to kill not only bacteria^[^ ^15^, ^18^ ^]^ but also mammalian cancer cells^[^ ^13^, ^14^ ^]^ and to destroy tissue and multicellular organisms by nanomechanical action.^[^ ^19^ ^]^

**Figure 1:**
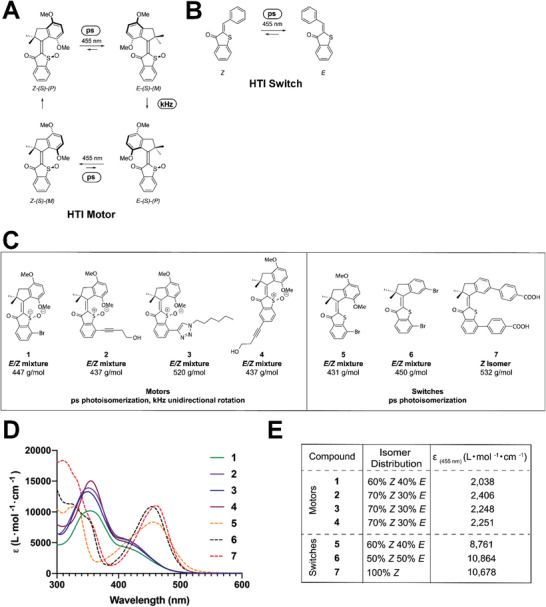
A) Rotation of one enantiomer (*S*) of an unfunctionalized HTI motor starting with the (*Z*) isomer. When illuminated with visible light, the motor photoisomerizes from *Z‐(S)‐(P)* to *E‐(S)‐(M)*. This photochemical step occurs in 7.9 ps. At 22 °C, the *E*‐*(S)‐(M)* form then thermally converts to *E‐(S)‐(P)* in 1.2 ms. The *E‐(S)‐(P)* form is subsequently photoisomerized to *Z‐(S)‐(M)* in a 1.5 ps process and then thermally converts to the *Z‐(S)‐(P)* in a 3.0 ns process.^[^ ^23^, ^104^ ^]^ B) Photoisomerization of an unfunctionalized HTI switch. When excited with visible light, the HTI switch converts from the more thermally stable *Z* isomer to the *E* isomer in 38 ps. The reverse *E* to *Z* photoisomerization occurs in 23 ps.^[^ ^24^ ^]^ HTI‐based photoresponsive molecules screened in this study for their antibacterial activity. C) Chemical structures of HTI motors and switches. D) Representative UV–vis spectra of HTI **1**–**7** in DMSO. E) Molar extinction coefficient at 455 nm of HTI motors and switches calculated from serial dilutions in DMSO and isomer distribution determined by ^1^H NMR.

Hemithioindigos (HTI) consist of an indigo or thioindigo half linked to a stilbene moiety via a central photoisomerizable C=C double bond.^[^ ^20^, ^21^ ^]^ As with Feringa‐type molecular machines, the unidirectional 360° rotation of HTI‐based molecular motors involves an initial photoisomerization step between the *E* and *Z* conformations, followed by a second thermal isomerization step that is rate‐determining (**Figure** **1**A), while “ON/OFF” HTI switches can only change between the *E* and *Z* conformations (Figure 1B).^[^ ^22^ ^]^ However, unlike Feringa‐type molecules, HTI‐based molecular machines do not have a defined motor or stator; instead, both parts rotate around the central double bond. In addition, HTI have the advantage of being activated by visible light (455 nm) farther from the UV range. Other advantages of HTI include high thermal stability of the *E* isomer and a bathochromically shifted (10–20 nm) absorbance of the *E* isomer relative to the *Z*, which allows the accumulation of large amounts of the desired isomer upon irradiation, as well as slower rotational speeds in the kHz range.^[^ ^23^, ^24^ ^]^ However, the applications of hemithioindigos in chemical biology are mostly limited to the conformational control of peptides.^[^ ^25^ ^]^ To our knowledge, the antimicrobial potential of visible light‐activated HTI‐based molecular machines has never been investigated.

In this work, we report the ability of visible light (455 nm)‐activated HTI‐based molecular machines to kill Gram‐positive bacteria without noticeable development of resistance and minimal toxicity to mammalian cells. Surprisingly, the “ON/OFF” HTI switches showed more potent antibacterial activity than the 360° unidirectional rotational “drilling” HTI motors, suggesting that the antimicrobial activity of these molecules is not due to mechanical action. Indeed, we show that the antibacterial activity of HTI‐based molecular machines was associated with enhanced generation of reactive oxygen species (ROS) and oxidative damage to biomolecules. This mechanism of action differs from that of previously described molecular machines that mechanically destroy cells,^[^ ^13^ ^]^ demonstrating that molecular machines with different chemical cores can have distinct modes of action. In addition to their direct antibacterial activity, HTI were found to synergize with conventional antibiotics to kill methicillin‐resistant *S. aureus* (MRSA) in vitro and in vivo and reduce infection‐related mortality and bacterial load. These findings identify light‐activated HTI‐based molecular machines that kill bacteria by oxidative stress as a safe narrow‐spectrum antibacterial therapy that can be used alone or in combination with conventional antimicrobial modalities.

## 2 Results

### 2.1 HTI Kill Gram‐Positive Bacteria

**Scheme 1:**
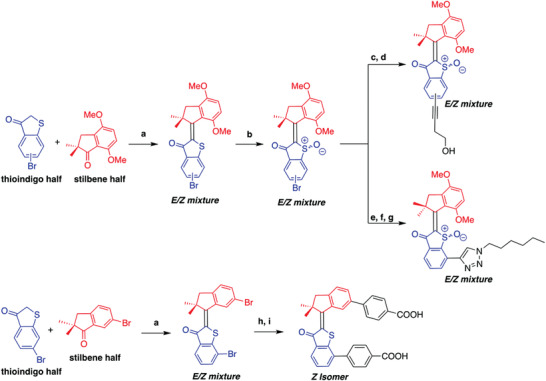
General synthesis of HTI motors and switches. a) BCl_3_, DCM −78 °C → 0 °C; b) NaBO_3_·4H_2_O, EtOAc, AcOH; c) (but‐3‐yn‐1‐yloxy)(*tert*‐butyl)dimethylsilane, Pd_2_(dba)_3_, PPh_3_, CuI, Et_3_N, THF, 70 °C; d) TBAF, THF; e) trimethylsilylacetylene, Pd_2_(dba)_3_, PPh_3_, CuI, Et_3_N, THF, 70 °C; f) K_2_CO_3_, MeOH, THF; g) 1‐azidohexane, CuSO_4_·5H_2_O, sodium ascorbate, H_2_O, CH_2_Cl_2_; h) 4‐(*tert*‐Butoxycarbonyl)phenylboronic acid pinacol ester, sSPhos Pd G2, K_3_PO_4_, EtOH, PhMe, 100 °C; i) HCOOH, CH_2_Cl_2_.

In this study, the antibacterial activities of seven different light‐activated HTI‐based molecular machines (four HTI motors and three HTI switches) with different optical properties were investigated (Figure 1C–E). The general HTI synthesis procedure is summarized in **Scheme** **1**. The motors and switches were synthesized via condensation reactions between the stilbene and thioindigo halves of the molecules. The motors were oxidized and both switches and motors were functionalized with palladium‐catalyzed cross‐coupling chemistry (Scheme 1). Further experimental details on the synthesis of the molecules can be found in the Supporting Information. A panel of six distinct Gram‐negative and seven Gram‐positive strains (Table S1, Supporting Information) was treated with increasing concentrations of different HTI molecules. The cells were then irradiated for 10 min with 455 nm light at 65 mW cm^−2^ (39 J cm^−2^), and after overnight incubation in Mueller–Hinton broth (MHB), the optical density at 600 nm (OD_600_) of the cell suspensions was determined. The HTI concentration resulting in complete growth arrest after 10 min of irradiation at 455 nm was defined as the minimal inhibitory concentration (MIC). For Gram‐positive strains, the MIC ranged from 10 to 320 × 10^−6^ m for the HTI motors and between 0.31 and 80 × 10^−6^ m for the HTI switches (**Table** **1**), corresponding to a median MIC of 160 × 10^−6^ m for the motors and a median MIC of 10 × 10^−6^ m for the switches. Complete growth arrest was detected only in irradiated samples, demonstrating the importance of light for the activation of HTI to exert antibacterial activity (Figure S1A, Supporting Information). For Gram‐negative bacteria, while significant growth inhibition (up to 80% based on OD_600_) could be detected at high HTI concentrations, complete growth arrest was not achieved under the experimental conditions tested (Figure S1B, Supporting Information). The vehicle (1% DMSO) plus 455 nm light (39 J cm^−2^) had only a marginal effect on bacterial growth (Figure S1C, Supporting Information), demonstrating that the observed antibacterial properties of HTI are not due to the vehicle.

**Table 1:** MIC values (× 10^−6^ m) for different HTI in the Gram‐positive and Gram‐negative bacteria examined in this study. The results are representative of at least three independent biological replicates. Further details are provided in the main text

	HTI **1**	HTI **2**	HTI **3**	HTI **4**	HTI **5**	HTI **6**	HTI **7**
**Gram‐positive**							
*S. aureus*	10	80	40	160	40	5	5
*S. epidermidis*	320	320	40	320	40	10	40
*B. subtilis*	10	10	160	20	40	1.25	0.31
*E. faecalis*	80	160	320	160	80	5	2.5
*B. megaterium*	80	160	40	80	2.5	0.63	0.63
*E. faecium*	160	320	320	160	40	20	5
MRSA	20	320	320	40	80	10	10
**Gram‐negative**							
*E. coli*	>320	>320	>320	>320	>320	>320	>320
*E. cloacae*	>320	>320	>320	>320	>320	>320	>320
*P. aeruginosa*	>320	>320	>320	>320	>320	>320	>320
*A. baumannii*	>320	>320	>320	>320	>320	>320	>320
*B. cepacia*	>320	>320	>320	>320	>320	>320	>320
*X. maltophila*	>320	>320	>320	>320	>320	>320	>320

**Figure 2:**
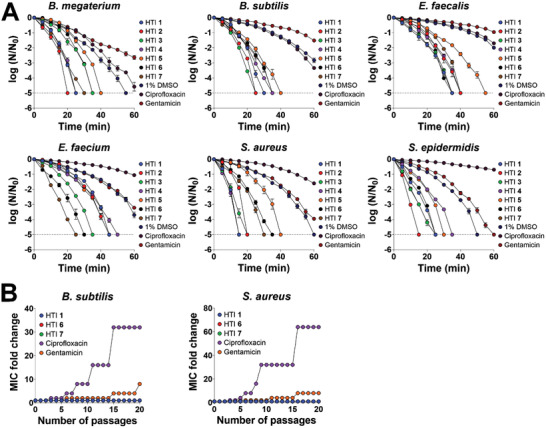
HTI kill Gram‐positive bacteria without detectable resistance. A) Time‐dependent reduction in CFU (expressed as the logarithm of base 10 of the ratio between the CFU value at every time point and the CFU value at time zero) of different exponentially growing Gram‐positive bacterial strains in the presence of 1% DMSO (solvent control) + 455 nm light at 65 mW cm^−2^, 1× MIC of each HTI + 455 nm light at 65 mW cm^−2^, or 2× the MIC of conventional antibiotics. The dashed line denotes the limit of detection of the method. All results are shown as the mean of at least three biological replicates ± standard error of the mean. B) MIC fold change relative to the original MIC following repeated exposure to different light‐activated HTI (10 min of irradiation with 455 nm light at 65 mW cm^−2^ corresponding to a light dose of 39 J cm^−2^) and control antibiotics in *B. subtilis* and *S. aureus*. Note that the curves for HTI **6** and **7** are not visible because they are superimposed on those of HTI **1**. Results are representative of three independent biological replicates. Unless otherwise noted, the results for HTI and DMSO are always reported in the presence of light.

The antibacterial properties of HTI were further investigated in time‐kill experiments with different Gram‐positive bacterial strains by treating cell suspensions with 1× MIC of different HTI (Table 1), followed by irradiation with 455 nm light at 65 mW cm^−2^ for up to 60 min. Comparison with samples treated with 1% DMSO and irradiated under the same conditions allowed the distinction between HTI‐induced effects and effects induced by light alone. Samples treated with 2× MIC of different antibiotics (Table S2, Supporting Information) were used as controls. Depending on the molecule, the bacterial cell number was reduced to the limit of detection of the method within 15–55 min of irradiation (**Figure** **2**A), vastly outpacing the performance of conventional antibiotics. Bacterial killing by HTI varied in a dose‐dependent manner, with higher light doses resulting in enhanced cell death, whereas at the same light dose, there was generally no significant effect of fluence rate on killing by HTI (Figure S2, Supporting Information).

The ability of *B. subtilis* and *S. aureus* to develop resistance to repeated exposure to visible light‐activated HTI was assessed by serial passage experiments.^[^ ^26^ ^]^ Cells surviving exposure to 0.5× MIC of the most potent light‐activated HTI (HTI **1**, **6**, and **7**) were collected and rechallenged with light‐activated HTI for up to 20 cycles of repeated treatment. The results were compared with those obtained after repeated exposure to the conventional antibiotics ciprofloxacin and gentamicin. While cells repeatedly treated with conventional antibiotics showed an increase in the MIC over time, the HTI MIC in *B. subtilis* and *S. aureus* remained constant for up to 20 cycles of repeated treatment (Figure 2B). Importantly, antibiotic‐resistant mutants retrieved from serial passage experiments did not exhibit cross‐resistance to HTI (Table S3, Supporting Information).

### 2.2 HTI Eliminate MRSA, Persisters, and Biofilms

**Figure 3:**
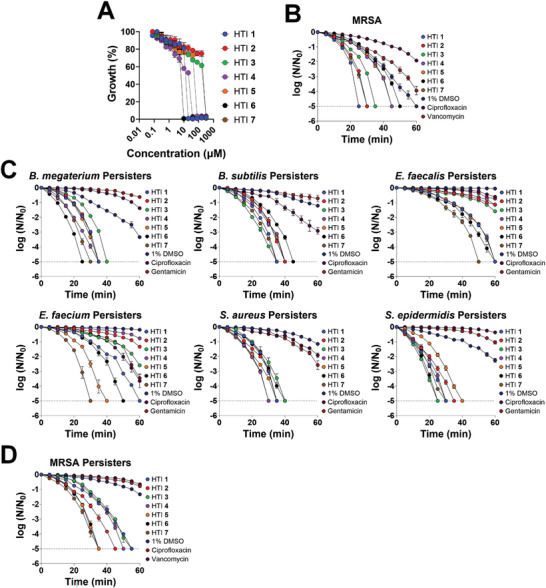
HTI eliminate antibiotic‐resistant and antibiotic‐tolerant persister cells of Gram‐positive bacteria. A) Growth inhibition of MRSA by different HTI in the presence or absence of light. Growth was assessed as OD600. Time‐dependent reduction in CFU (expressed as the logarithm of base 10 of the ratio between the CFU at every time point and the CFU at time zero) of B) exponentially growing MRSA, C) antibiotic‐tolerant persister cells of different Gram‐positive strains, or D) persisters of MRSA in the presence of 1% DMSO (solvent control) + 455 nm light at 65 mW cm^−2^, 1× MIC of each HTI + 455 nm light at 65 mW cm^−2^, or 2× the MIC of conventional antibiotics. The dashed line denotes the limit of detection of the method. All results are shown as the mean of at least three biological replicates ± standard error of the mean. Unless otherwise noted, the results for HTI and DMSO are always reported in the presence of light.

The ability of HTI to eliminate antibiotic‐resistant and antibiotic‐tolerant Gram‐positive strains was also investigated. A methicillin‐resistant, cefoxitin‐resistant strain of *S. aureus* (MRSA, *Staphylococcus aureus* Rosenbach ATCC BAA‐1680) was used as a representative of an antibiotic‐resistant Gram‐positive strain. In MRSA, the MIC of the different HTI ranged from 10 × 10^−6^ m (HTI **6**, HTI **7**) to 320 × 10^−6^ m (HTI **2**) (**Figure** **3**A). Treatment of exponentially growing MRSA with 1× MIC of different HTI reduced the bacterial numbers to the limit of detection in as little as 25 min (HTI **1**) of irradiation with 455 nm light at 65 mW cm^−2^ (Figure 3B).

The ability of HTI (1× MIC) to eradicate antibiotic‐tolerant persister cells of different Gram‐positive strains was also investigated. Cells were treated with 10× MIC linezolid for 24 h to eliminate active cells, after which persister cells were collected and washed to remove any trace of antibiotic.^[^ ^27^ ^]^ Depending on the bacterial strain and HTI, persister cells could be eradicated in as little as 25 min with 1× MIC of HTI, outperforming conventional antibiotics at even 2× MIC (Figure 3C). Treatment with 1× MIC of HTI also eradicated antibiotic‐tolerant persisters of MRSA in 35 min (HTI **5**, **6**, and **7**) to 55 min (HTI **1** and **3**) of light activation compared with an ≈1−log_10_ reduction observed in DMSO‐treated samples even after 60 min of irradiation (Figure 3D).

The antibiofilm activity of HTI was investigated by treating established biofilms of *S. aureus* and *B. subtilis* with 2× MIC of the most potent HTI (i.e., those with the lowest MIC in *B. subtilis* and *S. aureus*) or 1% DMSO, followed by 20, 40, or 60 min of irradiation with 455 nm light at 65 mW cm^−2^. Antibiotic controls (nisin in the case of *B. subtilis* and rifampin in the case of *S. aureus*) were processed similarly, except that light was omitted. Four parameters were used to evaluate antibiofilm potential: the total bacterial cell number was assessed using the fluorescence of acridine orange, metabolically active cells were estimated from intracellular ATP levels, total protein content was evaluated using fluorescein isothiocyanate (FITC) fluorescence, and total biofilm biomass was determined using the crystal violet method.

**Figure 4:**
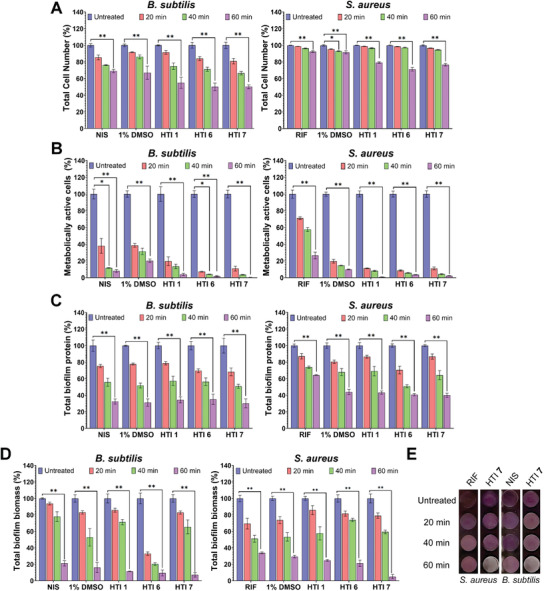
HTI display antibiofilm activity. Reduction in A) the total number of bacterial cells assessed using acridine orange, B) metabolically active cells assessed from ATP levels, C) total biofilm protein assessed using FITC fluorescence, and D) total biofilm biomass assessed using crystal violet in biofilms of *B. subtilis* and *S. aureus* after irradiation with 455 nm light at 65 mW cm^−2^ for 20, 40, and 60 min in the presence of 1% DMSO or 2× MIC of different HTI or in the presence of 2× MIC of conventional antibiotics. E) Representative biofilms of *B. subtilis* and *S. aureus* treated with 2× MIC of conventional antibiotics nisin and rifampin, respectively, or visible light‐activated HTI **7** for increasing amounts of time and then stained with crystal violet. Further experimental details are provided in the main text. NIS: nisin. RIF: rifampin. All results are shown as the mean of at least three biological replicates ± standard error of the mean. Asterisks denote the significance of the difference in pairwise comparisons using a Kruskal–Wallis test in GraphPad Prism. * *p* < 0.05, ** *p* < 0.01, *** *p* < 0.001, **** *p* < 0.0001. Unless otherwise noted, the results for HTI and DMSO are always reported in the presence of light.

Compared with untreated samples, visible light‐activated HTI reduced the total number of bacterial cells within the biofilms of *B. subtilis* and *S. aureus* by 29%–50% (*p* < 0.01), whereas DMSO and antibiotic treatment reduced the bacterial cell number by 10%–33% (*p* < 0.01) and 10%–31% (*p* < 0.01), respectively (**Figure** **4**A). Metabolically active cells within biofilms were reduced by 95%–100% (*p* < 0.01) following treatment with visible light‐activated HTI for 60 min, compared with a 73%–92% reduction in antibiotic‐treated samples (*p* < 0.01) and 80%–90% in DMSO‐treated samples (*p* < 0.01) (Figure 4B). Treatment with HTI, DMSO, or nisin resulted in a similar reduction in the total protein content of *B. subtilis* biofilms of 65%–70% (*p* < 0.01) compared with the respective untreated controls (Figure 4C). In *S. aureus*, treatment of biofilms with rifampin resulted in an average reduction in total biofilm protein of 36% compared with untreated controls, while samples treated with DMSO and HTI showed a similar reduction in total biofilm protein of up to 60% (Figure 4C). Compared with untreated samples, total biofilm biomass was reduced by up to 95% in HTI‐treated samples (*p* < 0.01), while treatment with antibiotics or DMSO reduced total biofilm biomass by up to 79% and 84%, respectively (Figure 4D). Figure 4E shows the superior antibiofilm performance of visible light‐activated HTI **7** (2× MIC) compared with antibiotics for the same treatment period.

### 2.3 HTI‐Induced Killing Is Mediated by ROS and Oxidative Damage

**Figure 5:**
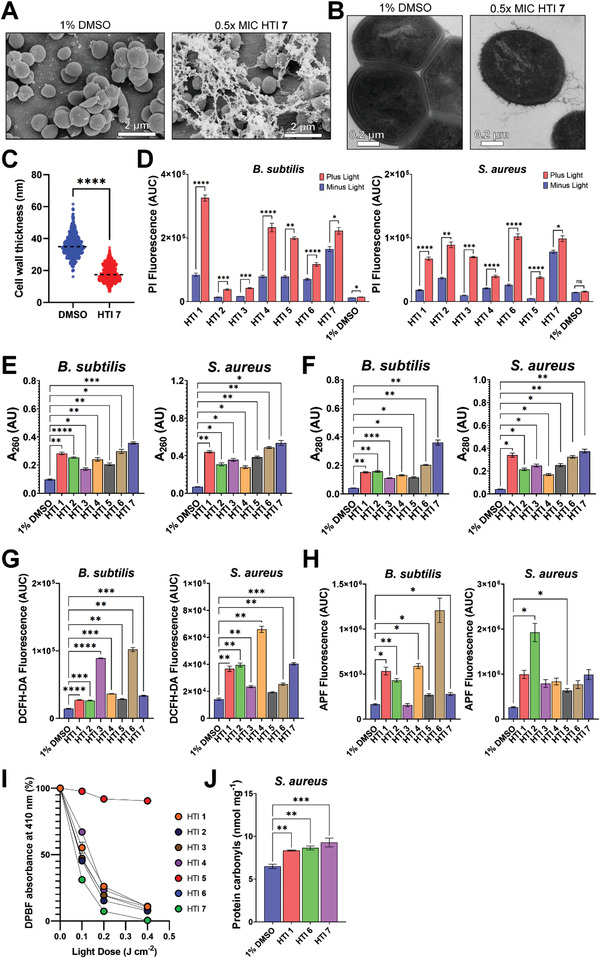
The mode of action of HTI involves altered membrane permeability, ROS generation, and protein oxidative damage. Ultrastructural changes of *S. aureus* in the presence of 1% DMSO or 0.5× MIC of HTI **7** plus 455 nm light (39 J cm^−2^) examined by A) SEM and B) TEM. C) Cell wall thickness of *S. aureus* treated with 1% DMSO or 0.5× MIC of HTI **7** plus 455 nm light (39 J cm^−2^). Cell wall length measurements from 90 cells were extracted from TEM images using ImageJ. The dashed line indicates the median. Samples were compared using a two‐tailed Mann–Whitney test in GraphPad Prism. D) HTI‐induced membrane damage assessed from PI uptake after treating *B. subtilis* and *S. aureus* with 1× MIC of different HTI in the presence and absence of 455 nm light (39 J cm^−2^). Leakage of cell contents after exposure to 2× MIC of HTI plus 455 nm light (39 J cm^−2^) was investigated by monitoring the absorbance of extracellular material at E) 260 nm (*A*_260_) and F) 280 nm (*A*_280_) corresponding to nucleic acids and proteins, respectively, in *B. subtilis* and *S. aureus*. ROS generation assessed with the fluorescent probes G) DCFH‐DA and H) APF following treatment of *B. subtilis* and *S. aureus* with 1× MIC of different HTI plus light (39 J cm^−2^). I) Decreased absorbance of DPBF at 410 nm, used as a proxy for singlet oxygen generation, in the presence of different HTI at 0.8 × 10^−3^ m after exposure to increasing doses of 455 nm light. J) Protein carbonyl levels in *S. aureus* treated with 2× MIC of HTI **1**, **6**, and **7** and irradiated with 455 nm light (39 J cm^−2^). All results are shown as the mean of at least three biological replicates ± standard error of the mean. Asterisks denote the significance of the difference in pairwise comparisons using a Kruskal–Wallis test in GraphPad Prism. * *p* < 0.05, ** *p* < 0.01, *** *p* < 0.001, **** *p* < 0.0001. Unless otherwise noted, the results for HTI and DMSO are always reported in the presence of light.

The effects of HTI on the ultrastructure of *S. aureus* were examined by transmission electron microscopy (TEM) and scanning electron microscopy (SEM) after sublethal (0.5× MIC) treatment with HTI **7** or 1% DMSO plus 455 nm light (39 J cm^−2^). SEM images revealed that *S. aureus* cells treated with sublethal doses of HTI showed extensive and widespread extrusion of extracellular polymeric material without overt changes in cell surface morphology (**Figure** **5**A). *S. aureus* cells treated with 0.5× MIC of HTI **7** also displayed substantial thinning of the cell wall compared to DMSO controls (Figure 5B), with the median cell wall thickness decreasing from 35.0 nm in DMSO‐treated cells to 17.4 nm (*p* < 0.0001) in HTI‐treated cells (Figure 5C). In addition, HTI‐treated cells displayed a discontinuous peptidoglycan layer and a highly irregular surface exhibiting multiple protrusions, also detected by SEM, compared with the smoother cell surface of DMSO‐treated cells (Figure 5B).

The impact of HTI on the membrane integrity of *B. subtilis* and *S. aureus* treated with 1× MIC of different HTI and irradiated with 455 nm light (39 J cm^−2^) was evaluated using the nucleic acid binding dye propidium iodide (PI). Compared with the dark controls, *B. subtilis* treated with 1× MIC of different HTI showed a 3.9‐fold increase (*p* < 0.0001) in PI uptake in irradiated samples, whereas DMSO‐treated samples displayed only a 1.2‐fold increase (*p* < 0.05) (Figure 5D). Compared with dark controls, *S. aureus* samples treated with 1× MIC of different HTI showed a 7.6‐fold increase in PI uptake (*p* < 0.0001), while DMSO‐treated samples showed only a nonsignificant rise (Figure 5D).

Leakage of cell contents in *B. subtilis* and *S. aureus* treated with 2× MIC of HTI and irradiated with 455 nm light (39 J cm^−2^) was investigated by monitoring the absorbance of extracellular material at 260 nm (*A*_260_) and 280 nm (*A*_280_), corresponding to nucleic acids and proteins, respectively. Compared with the corresponding DMSO controls, treatment of *B. subtilis* with different HTI resulted in a significant increase (*p* < 0.01) in *A*_260_ and *A*_280_ of up to 3.7‐ and 9.0‐fold, respectively (Figure 5E,F). Likewise, treatment of *S. aureus* with different HTI resulted in an increase in *A*_260_ of up to 7.8‐fold (*p* < 0.05) and an increase in *A*_280_ of up to 8.8‐fold (*p* < 0.01) (Figure 5E,F), compared with DMSO controls.

Mode of action studies proceeded by examining whether ROS and oxidative damage are involved in HTI‐induced antibacterial activity. Compared with the corresponding DMSO controls, samples treated with 1× MIC of different HTI plus 455 nm light (39 J cm^−2^) showed an increase in the fluorescence of the ROS‐sensitive probe 2′,7′‐dichlorofluorescein diacetate (DCFH‐DA) of up to 7.1‐ and 4.6‐fold (*p* < 0.01) in *B. subtilis* and *S. aureus*, respectively (Figure 5G). Likewise, treatment of *B. subtilis* and *S. aureus* with 1× MIC of HTI plus 455 nm light (39 J cm^−2^) resulted in an increase in the fluorescence of the hydroxyl radical‐specific probe 3′‐(*p*‐aminophenyl) fluorescein (APF) by up to sevenfold (*p* < 0.01) (Figure 5H). Singlet oxygen production by irradiated HTI was estimated using the singlet oxygen trap 1,3‐diphenylisobenzofuran (DPBF). UV–vis absorption spectra of DPBF in the presence of 0.8 × 10^−3^ m of different HTI and increasing doses of 455 nm light are shown in Figure S3 (Supporting Information). In all but HTI **5**, HTI irradiation resulted in a sharp reduction in the absorbance of DPBF at 410 nm (Figure 5I), reflecting the generation of singlet oxygen.

The increased ROS production in HTI‐treated samples was accompanied by the accumulation of oxidized proteins, denoted by a significantly higher (*p* < 0.01) level of protein carbonyls in *S. aureus* treated with 2× MIC of different light‐activated HTI compared with DMSO‐treated cells that were irradiated under the same conditions (Figure 5J).

**Figure 6:**
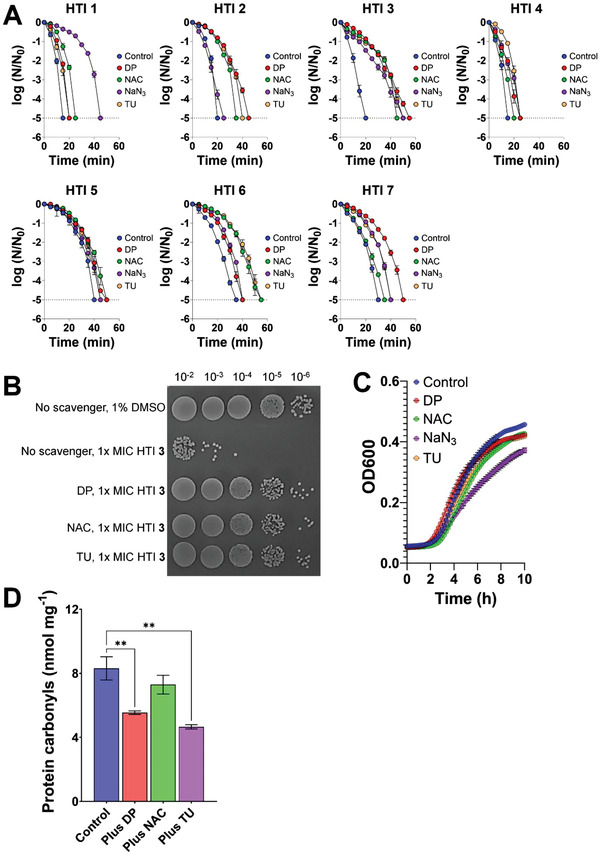
ROS scavengers mitigate HTI‐induced killing. A) Time‐dependent reduction in CFU (expressed as the logarithm of base 10 of the ratio between the cell number at every time point and the cell number at time zero) of *S. aureus* grown with and without iron (DP) and ROS (NAC, NaN_3_, TU) scavengers and subsequently challenged with 1x MIC of different HTI plus 455 nm light (39 J cm^−2^). The dashed line denotes the limit of detection of the method. B) Representative spot plate of *S. aureus* grown with and without scavengers (DP, NAC, or TU) and then challenged with 1% DMSO or 1× MIC of HTI **3** and irradiated with 455 nm light (39 J cm^−2^). C) Growth curves of *S. aureus* in the presence and absence of different scavengers (DP, NAC, NaN_3_, or TU). D) Protein carbonyl levels in *S. aureus* grown with and without different scavengers (DP, NAC, or TU) and then challenged with 2× MIC of HTI **3** and irradiated with 455 nm light (39 J cm^−2^). TU: thiourea, NAC: *N*‐acetyl‐l‐cysteine, NaN_3_: sodium azide, DP: dipyridyl. All results are shown as the mean of at least three biological replicates ± standard error of the mean. Unless otherwise noted, the results for HTI and DMSO are always reported in the presence of light. Asterisks denote the significance of the difference in pairwise comparisons using a Kruskal–Wallis test in GraphPad Prism. * *p* < 0.05, ** *p* < 0.01, *** *p* < 0.001, **** *p* < 0.0001.

The influence of different scavengers on the susceptibility of *S. aureus* to HTI‐induced killing was examined by growing *S. aureus* in the presence of the ROS scavengers *N*‐acetyl‐L‐cysteine (NAC, 1 × 10^−3^ m), sodium azide (NaN_3_, 2.5 × 10^−3^ m), and thiourea (TU, 100 × 10^−3^ m), or the iron scavenger 2,2′‐dipyridyl (DP, 0.5 × 10^−3^ m). Growth in the presence of scavengers increased the time necessary to eradicate bacterial populations by light‐activated HTI by 5 min to 35 min, compared with cells grown without scavengers (**Figure** **6**A). The protective effect of scavengers was particularly pronounced in the case of HTI **3**, resulting in an average increase in the time required for population eradication of 30 min. The spot plate pictured in Figure 6B shows that treatment of cells grown without scavenger with light‐activated HTI resulted in an ≈3 log_10_ reduction in cell numbers compared with DMSO‐treated samples. However, HTI‐induced killing was almost abolished in cells grown in the presence of different scavengers (Figure 6B). These observations provide further support for the role of ROS in HTI‐mediated killing. Importantly, with the possible exception of NaN_3_, the growth curves of *S. aureus* in the presence of scavengers were nearly identical to those of *S. aureus* without scavengers (Figure 6C), demonstrating that the protective effects of the scavengers are not a side effect of their impact on bacterial growth rate and/or doubling time.

To determine how ROS and iron scavengers mitigate HTI‐induced killing, protein oxidation products (protein carbonyls) were quantified in cells grown with scavengers (NAC, TU, or DP) or 1% DMSO (control) and then challenged with HTI **3** (2× MIC) plus 455 nm light (39 J cm^−2^). Protein carbonyl levels in cells grown with DP and TU and then treated with visible light‐activated HTI **3** were significantly lower (*p* < 0.01) than those detected in cells grown without scavenger (Figure 6D). This observation suggests that growth in the presence of scavengers mitigates HTI‐induced death by reducing the amount of oxidatively damaged biomolecules that arise after HTI treatment.

**Figure 7:**
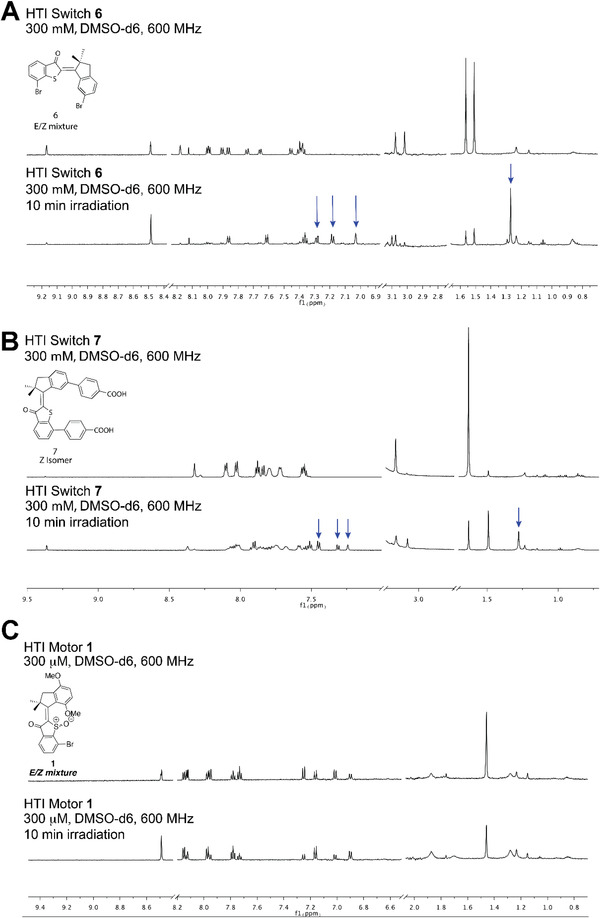
HTI motor **1** displays superior photostability than HTI switches **6** and **7**. Photostability of A,B) HTI switches **6** and **7** and C) HTI motor **1**. Representative NMR spectra of the molecule before (top) and after (bottom) light exposure. A 300 × 10^−6^ m sample of the HTI in DMSO‐d6 was prepared. The solution was added to an NMR tube, and a ^1^H NMR spectrum was acquired at 600 MHz. The sample was then emptied into a new 20 mL scintillation vial. The open vial was placed under the light source (LED Light, Prizmatix, UHP‐F‐455) and irradiated for 10 min with 455 nm at 65 mW cm^−2^, corresponding to a light dose of 39 J cm^−2^. The sample was then added back to the original NMR tube, and the NMR spectrum of the irradiated sample was acquired. Arrows indicate new peaks attributed to photobleaching and/or photodecomposition of the molecules upon irradiation. Note that in the case of HTI motor **1** the changes in the height of specific peaks reflect the expected changes in the relative abundance of the *E* and *Z* isomers of the molecule following irradiation.

The photostability of the most potent HTI molecules (HTI **1**, **6**, and **7**) was evaluated by acquiring the ^1^H NMR spectra of the molecules in DMSO‐d6 (HTI final concentration of 300 × 10^−6^ m) before and after irradiation with 39 J cm^−2^ of 455 nm light, the same irradiation conditions used for most bacterial assays. Irradiation of HTI switches **6** and **7** resulted in the appearance of new peaks in the NMR spectra, indicating some degree of photodecomposition of the molecules (**Figure** **7**A,B). In contrast, except for the expected changes in the relative abundance of the *E* and *Z* isomers, the spectra of the irradiated and nonirradiated HTI motor **1** were identical (Figure 7C), suggesting that the molecule did not undergo detectable photodecomposition.

### 2.4 HTI Potentiate the Antibiotic Killing of MRSA In Vitro and In Vivo

The observation that cells treated with HTI exhibited altered membrane permeability, as evidenced by increased PI uptake (Figure 5D) and leakage of intracellular contents (Figure 5E,F), prompted us to investigate the possibility that HTI might enhance the accessibility of antibiotics to their cellular targets and thus potentiate killing by antibiotics. To test this hypothesis, MRSA cell suspensions were challenged with 0.5× MIC of the most potent HTI molecules (HTI **1**, **6**, and **7**), followed by 10 min of irradiation with 455 nm light at 65 mW cm^−2^ (39 J cm^−2^). The cells were then treated with 0.5× MIC of the antibiotics ciprofloxacin or vancomycin. Controls consisted of cells treated with 0.5× MIC of HTI alone or 0.5× MIC of each antibiotic alone (Figure S4, Supporting Information).

**Figure 8:**
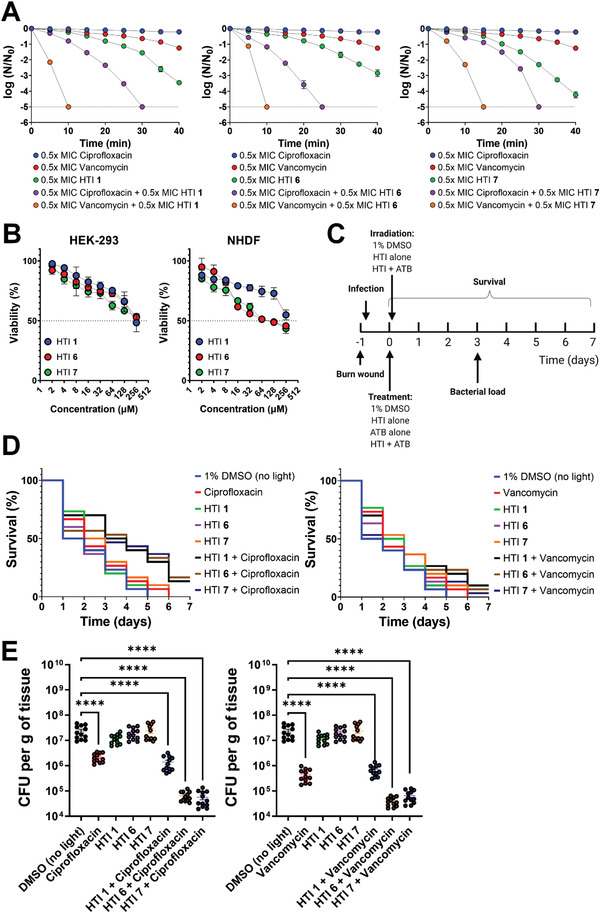
Light‐activated HTI synergize with conventional antibiotics to potentiate the killing of MRSA in vitro and in vivo. A) Time‐dependent reduction in colony‐forming units (expressed as the logarithm of base 10 of the ratio between the cell number at every time point and the cell number at time zero) of exponentially growing MRSA challenged with 0.5× MIC of HTI **1**, **6**, or **7** followed by 10 min of irradiation with 455 nm at 65 mW cm^−2^ and subsequent treatment with 0.5× MIC of antibiotics ciprofloxacin and vancomycin. Controls were treated with 0.5× MIC of light‐activated HTI alone (10 min with 455 nm light at 65 mW cm^−2^) or 0.5× MIC of each antibiotic alone. The dashed line denotes the limit of detection of the method. All results are shown as the mean of at least three biological replicates ± standard error of the mean. B) Viability of NHDF and HEK cells after treatment with increasing concentrations of HTI **1**, **6**, and **7** plus 455 nm light (39 J cm^−2^). All results are shown as the mean of at least three biological replicates ± standard error of the mean. The dashed line denotes the IC50 value, i.e., the concentration of HTI that results in a 50% reduction in cell viability after irradiation with 39 J cm^−2^ of 455 nm light. C) Schematic representation of the experimental setup used to assess the ability of HTI to potentiate antibiotic activity in vivo. ATB: antibiotic. D) Survival curves of *G. mellonella* infected with MRSA and then treated with 2× MIC of HTI **1**, **6**, or **7** followed by irradiation with 455 nm light (39 J cm^−2^) and subsequently challenged with 2× MIC of antibiotics ciprofloxacin and vancomycin compared with the survival of infected worms treated with 1% DMSO, HTI alone, or antibiotic alone. Data represent the pooled results of three independent biological replicates, each containing ten individuals (*n* = 30) per treatment group. D) Bacterial load (expressed as CFU per gram of larvae) on day 3 post‐treatment in *G. mellonella* infected with MRSA and subsequently treated with 1% DMSO, antibiotic alone, HTI alone, or a combination of 2× MIC of HTI followed by 2× MIC of antibiotics ciprofloxacin and vancomycin. Data represent the pooled results of three independent biological replicates, each containing four individuals (*n* = 12) per treatment group. Asterisks denote the significance of the difference in pairwise comparisons using a Kruskal–Wallis test in GraphPad Prism. * *p* < 0.05, ** *p* < 0.01, *** *p* < 0.001, **** *p* < 0.0001. Unless otherwise noted, the results for HTI and DMSO are always reported in the presence of light.

Treatment with 0.5× MIC of different HTI or antibiotics resulted in a maximal reduction in bacterial numbers of ≈4 log_10_ even after 40 min (**Figure** **8**A). However, in cells prechallenged with 0.5× MIC of light‐activated HTI, the time necessary to eradicate the MRSA population was reduced from over 40 min to as little as 10 min in cells subjected to dual therapy (Figure 8A), denoting increased susceptibility to killing by antibiotics.

The safety of the three most potent antibacterial HTI against MRSA (HTI **1**, **6**, and **7**) was investigated in two cell lines: human embryonic kidney cells (HEK‐293) and primary normal human dermal fibroblasts (NHDF). Mammalian cell lines were treated with increasing concentrations of different HTI (0–256 × 10^−6^ m) and irradiated with 455 nm light (39 J cm^−2^), the same conditions used to determine the bacterial MIC. Cell viability was then assessed by quantifying ATP levels. The HTI concentration necessary to reduce cell viability by 50% (IC50) in the presence of 39 J cm^−2^ of 455 nm light was found to be ≥256 × 10^−6^ m for HEK‐293 cells and ≥64 × 10^−6^ m for NHDF (Figure 8B).

The ability of HTI to synergize with antibiotics and mitigate MRSA‐induced mortality in vivo was investigated in a burn wound infection model of the invertebrate *G. mellonella*. Based on safety data, in vivo assays were performed using 2× MIC of different HTI in MRSA, corresponding to 40 × 10^−6^ m for HTI **1** and 10 × 10^−6^ m for HTI **6** and HTI **7**. After the generation of the burn wound, the larvae were treated with 2× MIC of different HTI. Following a 30 min incubation, the worms were irradiated with 455 nm light (39 J cm^−2^) and then treated with 2× MIC ciprofloxacin or vancomycin. Controls consisting of 1% DMSO alone in the presence and absence of light, 2× MIC of HTI alone in the presence and absence of light, or 2× MIC of individual antibiotics alone were also examined. The survival of the worms was then monitored for up to seven days (Figure 8C).

All (100%) MRSA‐infected worms treated with 1% DMSO in the presence and absence of light, as well as those treated solely with visible light‐activated HTI **1** and HTI **6**, were dead by day 5 post‐treatment (Figure 8D). By day 6, 100% of the worms treated with antibiotics alone were dead. However, up to 17% of worms treated with both light‐activated HTI and antibiotics were still alive by day 7 (*p* < 0.05), particularly for combinations with the antibiotic ciprofloxacin (Figure 8D and Table S4, Supporting Information).

An independent group of larvae processed as described for survival experiments was used to assess bacterial load on day 3 post‐treatment (Figure 8C). Compared with worms that were treated only with 1% DMSO, antibiotic treatment reduced the bacterial load by 1.7 log_10_ (*p* < 0.0001) (Figure 8E). However, worms that were treated with a combination of light‐activated HTI and antibiotics exhibited a reduction in bacterial load of up to 2.8 log_10_ (*p* < 0.0001) compared with DMSO controls (Figure 8E).

## 3 Discussion

Antibiotic resistance is a global public health problem that threatens millions of lives.^[^ ^1^ ^]^ However, while antibiotic‐resistant bacterial infections are on the rise, the development of new antimicrobial agents is virtually stagnant. Therefore, new antibacterial agents and therapies are urgently needed. Stimuli‐responsive synthetic materials, such as synthetic molecular machines, capable of providing on‐site, on‐demand antimicrobial action, offer the possibility of treating an infection locally with lower doses of antimicrobials, thus reducing the potential for the emergence and spread of antimicrobial resistance.^[^ ^9^, ^10^, ^28^ ^]^ However, some previously described molecular machines have indiscriminate destructive capabilities and dependence on dangerous UV and near‐UV wavelengths for activation.^[^ ^19^ ^]^

In this work, several visible light‐activated molecules containing a hemithioindigo core, enabling activation by visible light at 455 nm, were synthesized and screened for antibacterial activity. All HTI molecules exhibited light‐dependent bacteriostatic and bactericidal properties, killing all seven Gram‐positive strains tested only in the presence of light (Figure 2A). Interestingly, this effect was not observed in the Gram‐negative strains tested (Table 1), suggesting that the different cell wall compositions of the two groups of bacteria affect the antibacterial activity of HTI. Accordingly, increasing the permeability of the outer membrane of Gram‐negative bacteria by pretreatment with Tris‐EDTA resulted in increased sensitivity to HTI‐induced killing (Figure S5, Supporting Information). The ability to specifically kill Gram‐positive bacteria can reduce adverse events associated with antimicrobial therapy^[^ ^29^ ^]^ and minimize the side effects of broad‐spectrum antibiotics that target not only pathogens but also the many nonpathogenic bacteria that reside in the body and make up the microbiome.^[^ ^30^, ^31^ ^]^ In addition, narrow‐spectrum antibacterial agents are less likely to elicit the development of resistance because only a few bacterial species are affected.^[^ ^32^ ^]^

In some cases, light alone had a substantial detrimental effect on cell viability, especially in *B. megaterium*, *S. aureus*, and *S. epidermidis* (Figure 2A). In the case of *S. aureus*, this could be due to light‐induced disassembly of membrane microdomains, possibly as a result of photolysis of staphyloxanthin, the carotenoid pigment produced by some *S. aureus* strains.^[^ ^33^, ^34^ ^]^ However, eradication of the bacterial population by light alone required at least 10 min longer than in the samples treated with HTI, while *B. subtilis*, *E. faecalis*, and *E. faecium* could not be eradicated by light alone even after 60 min of irradiation (Figure 2A).

Importantly, unlike conventional antibiotics, repeated exposure to visible light‐activated HTI did not result in increased resistance to treatment, as indicated by the stability of the MIC value over 20 cycles of repeated exposure to HTI (Figure 2B). Furthermore, antibiotic‐resistant mutants obtained during serial passage experiments did not show cross‐resistance to HTI‐mediated killing (Table S3, Supporting Information).

In addition to exponentially growing cells, visible light‐activated HTI eradicated persister cells of different Gram‐positive strains in as little as 25 min, much faster than conventional antibiotics (Figure 3C). Persister cells represent a subfraction of any bacterial population that is slow growing or inactive and, therefore, tolerant to antibiotics that normally target biological processes associated with active growth.^[^ ^35^ ^]^ Antibiotic tolerance reduces the efficacy of antibiotics in vitro and in vivo and may also contribute to the development of antibiotic resistance.^[^ ^36^, ^37^, ^38^ ^]^

Visible light‐activated HTI also eliminated exponentially growing and persister cells of an antibiotic‐resistant strain of *S. aureus* (MRSA, Rosenbach ATCC BAA‐1680) (Figure 3A,B,D). MRSA is the most common drug‐resistant bacterium responsible for healthcare‐ and community‐acquired infections.^[^ ^39^, ^40^ ^]^ In the United States, at least 94 000 MRSA infections are estimated to occur each year, resulting in 18 650 MRSA‐related deaths.^[^ ^41^ ^]^ Worldwide, in 2019 over 100 000 deaths were attributed to MRSA.^[^ ^2^ ^]^ Since the various light‐activated HTI investigated in this study killed MRSA efficiently, the mechanisms responsible for antibiotic resistance in this bacterium are unlikely to confer cross‐resistance to HTI‐induced killing.

Similar to antibiotic‐tolerant persister cells, biofilms pose a significant challenge for the treatment of bacterial infections because they are naturally refractory to many types of antibiotics.^[^ ^42^, ^43^ ^]^ Even when biofilms are genetically sensitive to antibiotics, the presence of extracellular polymeric substances, as well as altered metabolic activity and/or gene expression, can contribute to antibiotic resistance in biofilms.^[^ ^44^ ^]^ In addition, there is growing evidence that higher mutation rates within the biofilm environment favor the emergence of antibiotic‐resistant mutants.^[^ ^45^ ^]^ Visible light‐activated HTI showed antibiofilm activity against the two Gram‐positive strains tested (*S. aureus* and *B. subtilis*), reducing bacterial cell numbers, total biofilm biomass, and biofilm protein as effectively or better than conventional antibiotics (Figure 4A–E).

Surprisingly, HTI switches (HTI **5**–**7**), which can only change between the *E* and *Z* conformations and thus have only two distinct positions, showed, in general, overall superior bacteriostatic properties (median switch MIC = 10 × 10^−6^ m) than HTI motors (HTI **1**–**4**, median motor MIC = 160 × 10^−6^ m), which can undergo 360° unidirectional rotation. This finding is in contrast with previous results obtained using Feringa‐type molecular machines^[^ ^14^, ^15^ ^]^ and appears to rule out light‐induced intramolecular rotation (i.e., mechanical action) alone as the underlying mechanism of action of the HTI investigated in this study.

In addition to intramolecular rotation, excited chromophores can return to the ground state through two other pathways of nonradiative decay: heat generation by internal conversion or intersystem crossing, which results in the formation of free radicals and ROS.^[^ ^46^, ^47^ ^]^ The observation that the sample temperature showed only a nonsignificant increase of 2.5 °C throughout the irradiation period of 60 min (Figure S6, Supporting Information) rules out bulk photothermal effects as the cause of the observed antibacterial activities of HTI.

In this work, HTI‐treated cells showed increased ROS formation compared with DMSO controls, as detected using the fluorescent probes DCFH‐DA and APF (Figure 5F,G). Furthermore, all but HTI **5** showed high rates of singlet oxygen production, as measured using the singlet oxygen trap DPBF (Figure 5I). Increased ROS and singlet oxygen production were accompanied by the accumulation of oxidative protein damage (protein carbonyls) (Figure 5J), denoting the involvement of oxidative stress in HTI‐mediated bacterial killing. Within the range of concentrations tested, competition experiments with the major phospholipids of the *S. aureus* membrane revealed no effect on HTI MIC, indicating that HTI do not physically bind the bacterial membrane (Figure S7, Supporting Information). Oxidative damage to the cell membrane is, thus, likely responsible for the observed membrane permeabilization (Figure 5D) and leakage of cell contents (Figure 5A,E,F) following treatment of cells with visible light‐activated HTI.

These observations suggest that HTI‐induced bacterial killing is mediated by intersystem crossing, a process in which the excited singlet state of the molecule undergoes spin inversion to an excited triplet state. The triplet state can then return to the ground state via the emission of a photon through phosphorescence. Alternatively, the excited triplet state can undergo two types of reactions with nearby triplet state molecules, particularly molecular oxygen (^3^O_2_): an electron transfer process that produces free radicals such as hydroxyl or superoxide radicals (Type I reaction) or an energy transfer process with ground triplet state diatomic oxygen that leads to its transition to the excited singlet oxygen state (Type II reaction).^[^ ^48^ ^]^ Both reactions can occur simultaneously, and the resulting ROS cause oxidative damage to biomolecules, ultimately leading to cell death.^[^ ^49^ ^]^

ROS release through intersystem crossing relaxation is the primary mechanism behind photodynamic therapy (PDT), an FDA‐approved, minimally invasive procedure used primarily for cancer treatment.^[^ ^50^ ^]^ PDT uses a pharmacologically inert chromophore called a photosensitizer, molecular oxygen, and light, which individually are not toxic but, in combination, trigger the production of harmful oxygen radicals that can kill cells.^[^ ^51^ ^]^

The involvement of ROS and oxidative stress in the antibacterial mechanism of action of HTI was further investigated by comparing the sensitivity of cells grown with and without ROS and iron scavengers to HTI‐induced killing. By chelating free iron, DP inhibits the Fenton reaction and the subsequent formation of the potent hydroxyl radical, while TU quenches the hydroxyl radical directly.^[^ ^52^, ^53^, ^54^ ^]^ NAC is also a potent scavenger of hydroxyl radicals, hydrogen peroxide, and hypochlorous acid, and to a lesser extent, also inhibits superoxide radical formation.^[^ ^55^ ^]^ Sodium azide (NaN_3_) is a well‐known singlet oxygen scavenger that can also react with hydroxyl radicals.^[^ ^56^ ^]^

Cells grown in the presence of various ROS scavengers were less susceptible to HTI‐induced killing, as evidenced by the requirement for more extended irradiation periods to achieve the same level of killing as untreated cells (Figure 6A,B). Protection by antioxidants, notably DP and TU, was associated with lower levels of oxidized proteins after HTI treatment compared to untreated samples (Figure 6D). These findings identify the mechanism of action of visible‐light‐activated HTI as involving increased ROS production, accumulation of oxidative damage, and ultimately cell death, i.e., photodynamic action via Type I and Type II mechanisms. This mechanism of action is also consistent with the ultrastructural changes detected by electron microscopy in HTI‐treated *S. aureus*, including cell wall thinning and content leakage, which are similar to those previously reported in PDT‐treated *S. aureus*.^[^ ^57^, ^58^, ^59^, ^60^ ^]^ In the case of *S. aureus*, photolysis of staphyloxanthin can also contribute to the rapid elimination of the bacterial population by visible light‐activated HTI by increasing membrane permeability and thus rendering *S. aureus* more susceptible to killing by HTI‐generated ROS.^[^ ^34^ ^]^

Untargeted oxidative damage to biomolecules could explain the lack of detectable development of resistance to repeated HTI treatment (Figure 2B) since resistance to such an attack would require the unlikely accumulation of numerous beneficial mutations in a single cell. A similar rationale has been given for the low likelihood of resistance development with antimicrobial PDT.^[^ ^61^ ^]^

Taken together, these results identify HTI‐based molecular machines as a new class of photosensitizers. Additionally, the identified mechanism of action of HTI‐based molecular machines differs from that of previously described Feringa‐type molecular machines that rely on mechanical action,^[^ ^13^, ^14^, ^15^ ^]^ demonstrating that different molecular machines can impact biological systems through distinct mechanisms.

The finding that HTI are photosensitizers led us to hypothesize that the differences in antibacterial activity among HTI might be due to differences in the parameters that make molecules good photosensitizers, in particular their ROS production ability, light absorption potential, or chemical stability following irradiation.^[^ ^51^, ^62^ ^]^ Overall, the ROS‐generating ability of the various molecules detected using the fluorescent probes DCFH‐DA and APF was similar for all molecules tested (Figure 5G,H). Likewise, except for HTI **5**, the least potent switch, which showed no singlet oxygen production, all other molecules generated comparable amounts of singlet oxygen (Figure 5I). However, the extinction coefficient of the switches was, on average, five times higher than that of the motors (Figure 1E). Thus, the substantially higher light absorption capacity of HTI switches probably explains their superior antibacterial activity relative to the motors.

Interestingly, the comparable antibacterial performance of HTI motor **1** and HTI switches **6** and **7** could be due to its superior photochemical stability, despite its lower light‐absorbing capacity, i.e., extinction coefficient. Indeed, while the irradiation of HTI motor **1** did not result in detectable photodecomposition (Figure 7C), the NMR spectra of irradiated HTI switches **6** and **7** revealed the appearance of new peaks, consistent with some degree of photochemical degradation of the molecules (Figure 7A,B). Therefore, the antibacterial activity of HTI‐based molecular machines depends on a trade‐off between light absorption and photostability.

Unlike existing Feringa‐type molecular machines,^[^ ^19^ ^]^ HTI showed low toxicity to eukaryotic cells, as evidenced by an IC50 (≥64 × 10^−6^ m) substantially higher than the HTI MIC in MRSA (≤20 × 10^−6^ m) (Figure 8B). The relatively low toxicity of HTI to mammalian cells may be attributed to charge differences between bacterial and mammalian membranes. Bacterial membranes are negatively charged due to a high proportion of anionic phospholipids, whereas mammalian membranes have a neutral charge due to the predominance of zwitterionic (neutral) phospholipids.^[^ ^63^, ^64^ ^]^ Other factors contributing to the selectivity of HTI toward bacterial cells include the absence of cholesterol in bacterial membranes,^[^ ^65^ ^]^ which in mammalian cells contributes to membrane stiffness preventing, for example, the penetration of antimicrobial agents.^[^ ^66^ ^]^ In addition, the presence of lipid species susceptible to photolysis, particularly in *S. aureus* strains, could contribute to increased bacterial susceptibility to ROS produced by irradiated HTI.^[^ ^34^ ^]^

Molecular machines hold great promise in numerous biomedical applications. Chief among these is the ability to deliver drugs directly into cells, thus reducing the side effects of systemic chemotherapeutic regimens. However, the biomedical applications of existing molecular machines are limited by their indiscriminate destructive capabilities through mechanical action at the molecular level.^[^ ^19^ ^]^ The inherent ability of HTI to selectively kill bacteria without damaging mammalian cells, due to their distinct ROS‐based mechanism of action, represents a complementary and significant advantage over previously described molecular machines.

Visible light‐activated HTI also potentiated antibiotic activity in vitro (Figure 8A) and in vivo, reducing mortality and bacterial load in *G. mellonella* infected with MRSA (Figure 8D,E). *G. mellonella* is an established, inexpensive, and low‐maintenance model for bacterial and fungal infections^[^ ^67^, ^68^ ^]^ with a complex innate immune system with similarities to that of mammals.^[^ ^69^, ^70^ ^]^ Importantly, immune responses to pathogens in *G. mellonella* and mice are correlated, demonstrating that the results obtained with this invertebrate model can provide information relevant to mammals.^[^ ^71^ ^]^

Potentiation of antibiotic activity by pretreatment with visible light‐activated HTI can be due to enhanced accessibility of the antibiotic to intracellular targets after HTI treatment, as previously reported for membrane‐active antimicrobial agents.^[^ ^72^, ^73^ ^]^ Potentiation of antibiotic activity by pretreatment with visible light‐activated HTI could also be due to orthogonal targeting of different processes in the cell: 1) oxidative stress triggered by visible light‐activated HTI, followed by 2) disruption of cell wall synthesis or DNA synthesis by vancomycin^[^ ^74^ ^]^ and ciprofloxacin,^[^ ^75^ ^]^ respectively. Moreover, destabilization of membrane microdomains,^[^ ^76^ ^]^ for instance, as a result of photolysis of membrane pigments,^[^ ^34^ ^]^ may also contribute to the increased susceptibility of HTI‐treated bacteria to antibiotics. These findings are also consistent with previous observations that antimicrobial PDT can enhance the activities of conventional antibiotics.^[^ ^77^ ^]^

Among different types of wounds, burns have one of the highest infection rates, estimated at about 20%, and infections account for 75% of mortality in burn patients.^[^ ^78^ ^]^ The efficacy of systemic antibiotics to treat burn wounds is limited by inadequate perfusion. Conversely, topical antimicrobial treatments require intensive and painful dressing changes and can impair wound healing by depositing toxic antimicrobial concentrations at the site of infection.^[^ ^79^, ^80^ ^]^ Due to the intense selective pressure associated with the use of high antibiotic doses at the injury site and the requirement for long treatment regimens,^[^ ^81^, ^82^, ^83^, ^84^, ^85^, ^86^ ^]^ burn wounds are also potential sites for the emergence and propagation of antibiotic resistance.

The most common bacterial pathogens associated with burns are the Gram‐positive strains *S. aureus* and *E. faecalis*.^[^ ^86^, ^87^ ^]^ *S. aureus*, in particular, is becoming increasingly difficult to treat as resistance to common antibiotics continues to emerge and spread. It is estimated that 30%–50% of clinical isolates of *S. aureus* are resistant to methicillin.^[^ ^88^ ^]^ While vancomycin is generally effective in killing MRSA, there is also an increasing trend toward vancomycin resistance among *S. aureus* clinical isolates, and strains resistant to both vancomycin and methicillin have been reported.^[^ ^89^ ^]^

Because of their location, skin wounds, such as burns, are particularly well suited for light‐mediated antimicrobial therapies. However, conventional photosensitizers are limited by their lack of specificity to bacteria, which can lead to collateral damage to host tissue.^[^ ^90^, ^91^ ^]^ In this work, we identify HTI as new photosensitizers that can rapidly and safely kill the bacterial species most commonly associated with burn infections, including MRSA, without the emergence of resistance. Future studies will show whether HTI, alone or in combination with conventional antibiotics and the host's innate immune system, can successfully treat infections in mammals.

## 4 Conclusions

To our knowledge, this is the first report on the antimicrobial activity of HTI‐based molecular machines in vivo and in vitro. The results obtained in this study identify visible light‐activated HTI as an antimicrobial therapy that selectively kills Gram‐positive bacteria, including antibiotic‐resistant MRSA and antibiotic‐tolerant persister cells and biofilms, without detectable resistance development and with minimal adverse effects on mammalian cells at therapeutic doses. Unlike other molecular machines that destroy cells by mechanical action, visible light‐activated HTI kill bacteria by increasing ROS production and oxidative damage. Finally, HTI not only exhibit antibacterial activity on their own but can also synergize with conventional antibiotics in vitro and in vivo to reduce infection‐associated mortality and bacterial load. These findings identify HTI‐based molecular machines as a promising new class of photosensitizers.

## 5 Experimental Section

### Synthetic Chemistry

Details on the synthesis and characterization of the HTI used in this study are provided in the Supporting Information.

### Strains and Reagents

The strains and cell lines used in this study, their growth conditions, and origin are listed in Table S1 (Supporting Information). Unless otherwise noted, all antibiotics and chemical reagents were purchased from Sigma (St. Louis, MO) and prepared in 100% DMSO or an appropriate solvent per the distributor's instructions.

### Preparation of Cells for Irradiation Experiments

Cells from glycerol stocks maintained at −80 °C were streaked onto agar plates to obtain single isolated colonies. A single colony was picked up from the plate and grown overnight in filter‐sterilized Luria broth (LB) growth medium at 30 or 37 °C (220 rpm), depending on the strain (Table S1, Supporting Information). After overnight incubation, the culture was diluted (1:50) in fresh filter‐sterilized growth media and incubated (30 or 37 °C, 220 rpm) until the optical density at 600 nm (OD_600_) was ≈1. Following centrifugation (15 min at 5000× *g*, 25 °C), the cells were resuspended in phosphate‐buffered saline (PBS) to a final OD_600_ ≈ 0.02.

### Irradiation Experiments

The chemical structures of the HTI screened in this study, along with their UV–vis spectral characteristics and *E*/*Z* ratios before photoexcitation determined by ^1^H NMR, are shown in Figure 2A. The appropriate volume of HTI stock (16 × 10^−3^ m in DMSO) necessary to achieve the desired concentration of the test molecule was transferred to a microcentrifuge tube to which bacterial cells (OD_600_ ≈ 0.02) were then added. The corresponding negative controls (DMSO only) were prepared similarly. The mixture was incubated in the dark at 30 or 37 °C for 30 min with agitation (220 rpm). HTI‐ or DMSO‐treated cells were then transferred to a glass beaker positioned in the center of the light beam (455 nm LED Light, Prizmatix UHP‐F‐455, Israel) placed at the appropriate distance necessary to achieve an intensity of 65 mW cm^−2^, as measured using an S415C thermal power sensor head (Thorlabs, Newton, MA). The beaker was placed inside a water tray to minimize sample overheating during irradiation. The temperature during irradiation was monitored using a thermocouple probe (Model SC‐TT‐K‐30‐36‐PP; Omega Engineering, Inc., Stanford, CT). Throughout the irradiation period, the temperature ranged from 19.9 to 22.7 °C (Figure S6, Supporting Information). Samples were agitated during irradiation. Dark controls were prepared as previously described, except that light was omitted.

### Minimal Inhibitory Concentration (MIC)

For MIC determination, samples were treated with a range of concentrations (0–320 × 10^−6^ m) of different HTI and irradiated one by one for 10 min with 455 nm light at 65 mW cm^−2^, corresponding to a dose of 39 J cm^−2^. After irradiation, samples were collected and inoculated in MHB and incubated overnight (30 or 37 °C) without agitation. Corresponding nonirradiated samples and negative controls (without bacteria) were processed similarly. OD_600_ was determined on a spectrophotometer after incubation overnight, and the MIC was identified. All experiments were conducted at least in triplicate.

### Time‐Kill Experiments

For time‐kill experiments, bacterial cell suspensions prepared as described above were treated with 1× MIC of each HTI and irradiated with 455 nm light at 65 mW cm^−2^ for up to 60 min. To account for possible inhibitory effects of light alone, DMSO controls were prepared by adding 1% DMSO to the cell suspension followed by irradiation, as described for HTI‐treated cells. Antibiotic controls were prepared by adding ciprofloxacin, gentamicin, or vancomycin at 2× MIC (Table S2, Supporting Information) to the cell suspension and incubating the samples in the dark for up to 60 min. Sample aliquots were collected every 5 min throughout the treatment period. Serial dilutions were subsequently prepared in PBS and spotted onto LB agar plates. The plates were incubated overnight at the appropriate temperature (Table S1, Supporting Information) for each strain, and the number of colony‐forming units per milliliter (CFU mL^−1^) was determined. The results were expressed as log_10_ (*N*/*N*_0_), where *N* is the CFU mL^−1^ at each irradiation time point, and *N*_0_ is the initial CFU mL^−1^ of the corresponding sample. The limit of detection of the method was ≈100 CFU mL^−1^. All experiments were conducted at least in triplicate.

### Preparation and Eradication of Persister Cells

Antibiotic‐tolerant persisters were generated as previously described.^[^ ^27^ ^]^ Briefly, bacterial cultures were grown with agitation (220 rpm) at the appropriate temperature (Table S1, Supporting Information) in LB broth to an OD_600_ ≈ 0.3. The cells were then diluted 1:1000 in fresh LB and grown for an additional 16 h at the appropriate temperature at 220 rpm. Linezolid at 10× MIC was then added to the cell suspensions to eliminate antibiotic‐sensitive cells. After 24 h of treatment, the cells were collected and washed to remove any trace of antibiotics and then resuspended in PBS to a final OD_600_ ≈ 0.02. The persister cells were then challenged with 1× MIC of HTI or 1% DMSO followed by 455 nm light at 65 mW cm^−2^, as described for exponential phase cells. Antibiotic controls (2× MIC) were processed similarly, except that light was omitted. Sample aliquots were collected at 5‐min intervals for 60 min, serially diluted, and spotted onto LB agar plates to determine colony‐forming units per mL (CFU mL^−1^). Survival was determined by dividing the CFU mL^−1^ of the sample at each time point by the initial CFU mL^−1^ for that sample, as described for exponential phase cells. All experiments were conducted at least in triplicate.

### Antibiofilm Potential of HTI

The antibiofilm potential of the three most potent HTI, i.e., those with the lowest MIC in both *B. subtilis* and *S. aureus* (HTI **1**, **6**, and **7**), was assessed in a 96‐well plate format using a combination of methods targeting different components of the biofilm: total bacterial cell number, metabolically active cells, total biofilm protein, and biofilm biomass. This combination of methods was previously reported to be effective in evaluating the antibiofilm potential of chemicals.^[^ ^92^ ^]^ *B. subtilis* and *S. aureus* were grown overnight in tryptic soy broth medium (TSB) at 30 and 37 °C, respectively. The overnight cultures were then diluted at 1:100 in fresh media, and 100 µL aliquots were distributed in a 96‐well plate. After 24 h of static growth at the appropriate temperature, planktonic cells were removed by plate inversion, and the biofilm was washed three times with PBS. After washing, 1% DMSO or 2× MIC of each visible light‐activated HTI was added to the biofilm and incubated statically in the dark for 60 min. The biofilm was then irradiated for 20, 40, and 60 min with 455 nm light at 65 mW cm^−2^. Control antibiotics at 2× MIC were also included and processed as described for HTI, except that light was omitted.

To determine the total bacterial cell number, acridine orange solution (0.02% in Walpole buffer) was added to the wells. After 15 min of incubation, the biofilm was thoroughly washed with 0.9% NaCl and then resuspended in 100 µL of 0.9% NaCl. Fluorescence intensity (excitation: 485 nm, emission: 528 nm) was measured in a microplate reader (BioTek Instruments Inc, Winooski, VT).^[^ ^92^ ^]^

To quantify metabolically active cells in biofilms, 100 µL of TSB was added to each well, and the plate was thoroughly mixed to remove cells from the biofilm. A volume of 100 µL of BacTiter‐Glo reagent (Promega, WI, USA), prepared according to the manufacturer's instructions, was then added to each well; after 5 min of incubation, luminescence was measured on a microplate reader (BioTek Instruments Inc, Winooski, VT).^[^ ^92^ ^]^

To quantify total biofilm protein, FITC solution (20 µg mL^−1^) was added to each well. After a 30 min incubation, the biofilm was thoroughly washed with 0.9% NaCl and resuspended in 100 µL ddH_2_O. The fluorescence intensity was measured (excitation: 485 nm, emission: 528 nm) in a microplate reader (BioTek Instruments Inc., Winooski, VT).^[^ ^92^ ^]^

For total biofilm biomass quantification, 100 µL of TSB medium was added to the irradiated biofilm, which was allowed to recover for 24 h at 30 or 37 °C for *B. subtilis* and *S. aureus*, respectively. Planktonic cells were then removed by inversion of the plate, and the biofilm was washed with water. Then, a 0.1% solution of crystal violet was added to the washed biofilm. After 15 min of incubation, the biofilm was rinsed with water and dried overnight, after which crystal violet was solubilized with acetic acid (30% in water). The solubilized crystal violet was then transferred to a new flat‐bottom microtiter plate. The absorbance at 550 nm was quantified in a microtiter plate reader (BioTek Instruments Inc, Winooski, VT) using 30% acetic acid in water as the blank.^[^ ^93^ ^]^

Untreated sample values minus background were defined as 100% and used to calculate the reduction in biofilm parameters after treatment. All experiments were conducted at least in triplicate.

### Resistance Development by Serial Passage

To assess the development of resistance by serial passage experiments,^[^ ^26^ ^]^ *B. subtilis* and *S. aureus* cells in the exponential phase were collected and processed as described to determine the MIC. The cells were incubated at the appropriate temperature for 24 h, after which they were inspected for growth. Cells able to grow at 0.5× MIC of each HTI were then collected and rechallenged with a range of HTI concentrations and then irradiated for 10 min with 455 nm light at 65 mW cm^−2^ (39 J cm^−2^) for 20 successive cycles. Cells treated with the antibiotics ciprofloxacin and gentamicin were processed similarly, except that light was omitted, and were used as controls. All experiments were conducted at least in triplicate.

### Electron Microscopy

*S. aureus* cell suspensions (OD_600_ ≈ 0.05 in PBS) were prepared as previously described and treated with 0.5× MIC of HTI **7** or 1% DMSO and incubated in the dark for 30 min to allow HTI binding to the cell. The cell suspensions were then irradiated for 10 min with 455 nm light at 65 mW cm^−2^ (39 J cm^−2^). The cells were fixed with Karnovsky fixative, post‐fixed with 1% osmium, and dehydrated with a series of ethanol washes. For TEM, samples were embedded in epoxy resin (PolyBed 812; Polyscienses, Inc., Warrington, PA) after dehydration in a series of graduated washes with 50%–100% ethanol. Ultrathin sections (65 nm) were cut using an ultramicrotome (Leica EM UC7, Leica Microsystems, Wetzlar, Germany) and post‐stained with lead citrate and uranyl acetate. Specimens were observed using a TEM instrument (Hitachi Corporation, Japan) operating at an accelerating voltage of 80 kV. Cell wall thickness was determined from TEM images using ImageJ.^[^ ^94^ ^]^ A total of 90 cells were analyzed, and for each cell, six independent measurements of the cell wall were extracted from the images to obtain a total of 640 data points.

For SEM, after ethanol dehydration, samples were critical‐point dried using a Leica EM CPD300 (Leica Microsystems, Wetzlar, Germany) and sputter‐coated with 10 nm of gold. An FEI Apreo SEM (FEI Apreo, ThermoFisher Scientific, Waltham, MA) equipped with a secondary electron detector was used for image acquisition.

### Membrane Damage

Cells of *B. subtilis* and *S. aureus* were prepared as previously described and treated with 1× MIC of different HTI or 1% DMSO, followed by 10 min of irradiation with 455 nm light at 65 mW cm^−2^ (39 J cm^−2^). PI (2 µg mL^−1^ final concentration) was added to the cells following irradiation. The mixture was incubated for 30 min at room temperature, after which the stained bacterial suspensions were transferred to a black 96‐well plate. The time‐dependent progression of fluorescence intensity (excitation: 535 nm, emission: 620 nm) was monitored in a microplate reader (BioTek Instruments Inc., Winooski, VT).^[^ ^95^ ^]^ Following background subtraction, the area under the curve (AUC) of the temporal profiles of PI fluorescence was extracted and used for the statistical comparison of PI uptake in the different treatments. All experiments were conducted at least in triplicate.

### Leakage Assay

Intracellular content leakage following treatment with HTI was determined by examining the absorbance of leaked nucleic acids (260 nm) and proteins (280 nm).^[^ ^96^ ^]^ Cell suspensions (OD_600_ ≈ 0.2) were treated with 1% DMSO or 2× MIC of HTI. Following irradiation for 10 min with 455 nm light at 65 mW cm^−2^ (39 J cm^−2^), the cells were collected by centrifugation (10 000× *g*, 10 min). The absorbance of the supernatant at 260 (*A*_260_) and 280 nm (*A*_280_) was determined using a UV cuvette in a Beckman Coulter DU‐800 spectrophotometer (Beckman Coulter, Fullerton, CA). Hexadecyltrimethylammonium bromide (CTAB) was used as the positive control. The results were expressed as arbitrary units (AU) corrected for absorbance in the absence of cells. All experiments were conducted at least in triplicate.

### Estimation of ROS Levels

Intracellular ROS generation was quantified using the fluorescent probes DA and APF.^[^ ^56^, ^97^ ^]^ *B. subtilis* and *S. aureus* cell suspensions (OD_600_ ≈ 0.2 in PBS) were treated with 1% DMSO or 1× MIC of different HTI. After a 30‐min incubation in the dark, cells were irradiated for 10 min with 455 nm light at 65 mW cm^−2^ (39 J cm^−2^). DCFH‐DA (4 × 10^−6^ m, Sigma, MO, USA) or APF (10 × 10^−6^ m, AAT Bioquest, CA, USA) was then added to the cells, and after a 20 min incubation period in the dark, the fluorescence intensity was measured (excitation: 485 nm, emission: 525 nm for DCFH‐DA; excitation: 490 nm, emission: 515 nm for APF) in a microplate reader (BioTek Instruments Inc, Winooski, VT). Following background subtraction, the AUC of the temporal profiles of DCFH‐DA and APF fluorescence was extracted and used for the statistical comparison of ROS and hydroxyl radical levels, respectively, in the different treatments.

Singlet oxygen generation by irradiated HTI was estimated using the singlet oxygen indicator DPBF.^[^ ^98^ ^]^ Briefly, an aqueous solution of DPBF (2 × 10^−3^ m) containing 0.8 × 10^−3^ m of HTI was exposed to increasing doses of 455 nm light in a quartz cuvette. UV–vis spectra of DPBF were then immediately acquired in a Beckman Coulter DU 800 UV/Visible spectrophotometer (Beckman Coulter, Fullerton, CA). The oxidation of DPBF by singlet oxygen was assessed from the decrease in the absorbance of DPBF at 410 nm. All experiments were conducted at least in triplicate.

### Protein Carbonyl Quantification

*S. aureus* cell suspensions were treated with 2× MIC of HTI **1**, HTI **6**, or HTI **7**, or 1% DMSO and irradiated for 10 min with 455 nm light at 65 mW cm^−2^ (39 J cm^−2^), as previously described. After irradiation, cell suspensions were collected by centrifugation and resuspended in PBS containing a cocktail of proteinase inhibitors (Halt^TM^ protease inhibitor cocktail, Thermo Fisher Scientific, MA, USA) and 0.005% butylated hydroxytoluene to prevent further protein degradation and oxidation. Cells were lysed using a pulse sonicator (Misonix S 4000, Newtown, CT), after which the supernatant was harvested by centrifugation (12 000× *g*, 10 min). Following the determination of protein concentration using the Pierce assay (Pierce BCA Protein Assay Kit, Thermo Fisher Scientific, MA, USA), the protein concentration was adjusted to 10 µg mL^−1^. Protein carbonylation levels were then quantified using a protein carbonyl ELISA kit (Abcam, no. ab238536, Abcam, Cambridge, MA)^[^ ^99^ ^]^ according to the manufacturer's instructions. All experiments were conducted at least in triplicate.

### Influence of Growth in the Presence of ROS Scavengers on Susceptibility to HTI‐Induced Killing

To test the influence of growth in the presence of scavengers on sensitivity to visible light‐activated HTI, *S. aureus* was grown overnight in LB (37 °C, 220 rpm) as previously described, harvested by centrifugation, resuspended in 50 mL of fresh LB medium to OD_600_ ≈ 0.1 and further grown aerobically at 37 °C until mid‐log phase (OD_600_ ≈ 0.5), at which point freshly prepared ROS scavengers NAC (1 × 10^−3^ m), NaN_3_ (2.5 × 10^−3^ m), or TU (100 × 10^−3^ m), or iron scavenger DP (0.5 × 10^−3^ m), were added to the cell culture.^[^ ^53^, ^100^ ^]^ Controls were treated with 1% DMSO. Following a 3 h incubation at 37 °C, cells were collected by centrifugation, and cell suspensions were prepared as described above and treated with 1× MIC of different HTI. The cells were then irradiated with 455 nm at 65 mW cm^−2^ for different time periods and processed as described for time‐kill experiments for CFU determination.

For growth curve acquisition, overnight cell cultures of *S. aureus* were diluted 1000‐fold in fresh LB medium. NAC (1 × 10^−3^ m), NaN_3_ (2.5 × 10^−3^ m), TU (100 × 10^−3^ m), or DP (0.5 × 10^−3^ m) were then added to the cell culture. Controls were treated with 1% DMSO. Cell suspensions were then transferred to a 96‐well plate, and mineral oil was added on top to prevent evaporation during incubation. Growth curves at 37 °C were acquired by monitoring the OD_600_ every 10 min in a microplate reader (BioTek Instruments Inc., Winooski, VT).

The effect of growth in the presence of scavengers on biomolecule oxidation was tested by growing *S. aureus* in the presence of NAC (1 × 10^−3^ m), TU (100 × 10^−3^ m), DP (0.5 × 10^−3^ m), or 1% DMSO. Cells grown under different conditions were then irradiated with 2× MIC of HTI **3** (the HTI for which growth with and without scavenger had the most pronounced effect on time‐kill curves). After irradiation with 455 nm light at 65 mW cm^−2^ (39 J cm^−2^) for 10 min, the cells were collected, and the protein carbonyl levels were quantified as described above. All experiments were conducted at least in triplicate.

### Photochemical Stability of HTI

To evaluate the photostability of the most potent HTI (HTI **1**, **6**, and **7**), HTI solutions (300 × 10^−6^ m) were prepared in DMSO‐d6. Each solution was then placed in an NMR tube, and their respective ^1^H NMR spectra were recorded at 600 MHz. The sample was then emptied into a new 20 mL scintillation vial. The open vial was placed under the light source (LED Light, Prizmatix, UHP‐F‐455) and irradiated with 455 nm at 65 mW cm^−2^ for 10 min, corresponding to a light dose of 39 J cm^−2^. The sample was then returned to the original NMR tube, and the NMR spectrum of the irradiated sample was recorded under the same conditions as described previously. The NMR spectra before and after irradiation were then visually inspected to assess photodecomposition.

### HTI‐Mediated Potentiation of MRSA Killing by Antibiotics

MRSA cell suspensions were prepared as described above and treated with 0.5× MIC of the most potent HTI, i.e., the ones with the lowest MIC in MRSA (HTI **1**, **6**, and **7**), as described above for time‐kill experiments. Following 10 min of irradiation with 455 nm light at 65 mW cm^−2^ (39 J cm^−2^), cells were collected and challenged with 0.5× MIC of the antibiotics ciprofloxacin or vancomycin (Table S2, Supporting Information). Aliquots were collected every 5 min for up to 40 min of treatment time, diluted, and plated as described above for CFU determination. HTI controls consisted of cells treated with 0.5× MIC of HTI and irradiated for up to 40 min, without subsequent antibiotic treatment. Antibiotic controls consisted of cells treated with 0.5× MIC of ciprofloxacin or vancomycin for up to 40 min in the dark. All experiments were conducted at least in triplicate.

### Toxicity Profiling and Therapeutic Index Calculation

The biocompatibility of HTI with mammalian cells was assessed in two mammalian cell lines: NHDF and HEK‐293. Cells were treated with increasing concentrations (0–256 × 10^−6^ m) of the most potent HTI (HTI **1**, **6**, and **7**) and irradiated for 10 min with 455 nm light at 65 mW cm^−2^ (39 J cm^−2^), the same irradiation conditions used for the determination of bacterial MIC. The viability of irradiated cells was assessed using the CellTiter‐Glo Luminescent Cell Viability Assay (Promega, Madison, WI) according to the manufacturer's instructions, and HTI concentrations that reduced cell viability by 50% (IC50) were identified. All experiments were conducted at least in triplicate.

### Animal Infection Model

Animal studies were carried out in a burn wound model of the invertebrate *Galleria mellonella*.^[^ ^101^ ^]^ Work in *Galleria mellonella* was reviewed and approved by the Office of Sponsored Projects and Research Compliance (SPARC) of Rice University. Larvae were purchased from a commercial source (https://www.rainbowmealworms.net/) at a stage in their life cycle that does not require feeding. Larvae were sterilized with 70% ethanol, after which a burn wound was applied to the middle section of the back of the larvae using a soldering iron (WE1010 ESD‐Safe Digital 70‐Watt Soldering Station – 120 V, PA, USA) heated to 100 °C and applied for a fixed time of 5 s. This resulted in a consistent burn area of 2 mm^2^. Immediately after the generation of the burn, the wound was infected with MRSA (10 µL of 1:10 dilution of an overnight culture). Larvae showing signs of distress or hemolymph leakage following the burn generation were immediately euthanized by incubating at −20 °C for 20 min to minimize suffering. The larvae were incubated overnight at 37 °C to establish infection, after which 10 µL of 1% DMSO, HTI, and/or antibiotic was applied to the wound surface. The following treatments were considered: 1) HTI (**1**, **6**, or **7**) at 2× MIC with and without irradiation; 2) ciprofloxacin or vancomycin at 2× MIC; 3) 1% DMSO with and without irradiation; and 4) HTI (**1**, **6**, or **7**) at 2× MIC followed by treatment with ciprofloxacin or vancomycin at 2× MIC. A schematic representation of the experimental setup used to assess the anti‐infective potential of HTI alone and in combination with antibiotics is shown in Figure 8C. The light regimen of the irradiated samples was always the same: 10 min of irradiation with 455 nm at 65 mW cm^−2^ (39 J cm^−2^). Ten worms from three independent batches were used for each treatment (i.e., 30 individuals per treatment). The survival of the worms was then monitored for up to seven days. Mortality was defined by complete melanization of the larval body and/or absence of stimuli‐induced motility.^[^ ^102^ ^]^ A parallel set of larvae treated exactly as described to monitor survival was used to assess the effects of different treatments on the bacterial load on day 3 post‐treatment. Four worms from three independent batches were used for each treatment (i.e., 12 individuals per treatment). Only healthy larvae that did not show melanization spots and were responsive to stimuli were used to determine the bacterial load. After determining their weight, the worms were rapidly killed by freezing^[^ ^103^ ^]^ and then homogenized in PBS using a tissue grinder (Fisherbrand, Fisher Scientific, Pittsburgh, PA) on day 3 after treatment. Tenfold serial dilutions of the homogenate were prepared and plated onto TSA plates. After overnight incubation at 37 °C, CFU were counted and normalized to the weight of the larvae.

### Statistical Analysis

Unless otherwise noted, the arithmetic mean (average) and standard error of the mean across at least three biological replicates were used as measures of center and spread. In all the cases, no data points were excluded as outliers. When necessary, the data was Min‐Max normalized. Depending on the sample size, the normality of the data was assessed using an Anderson–Darling normality test, a D'Agostino–Pearson omnibus normality test, a Shapiro–Wilk normality test, or a Kolmogorov–Smirnov normality test using the Dallal–Wilkinson–Lilliefors test for *p*‐value. Comparisons between two groups were performed with a *t*‐test for parametric data or a Mann–Whitney U test for nonparametric data. Comparisons between multiple groups were performed using ANOVA or a Kruskal–Wallis test with Dunnett's multiple comparisons test. A Mantel–Cox test was used to determine statistical significance in *G. mellonella* survival experiments. The sample size for each statistical test is included in the respective legend of each figure. A value of *p* < 0.05 was considered statistically significant. GraphPad Prism 8.0 (San Diego, CA) was used for all statistical analysis and generation of graphs.

## Conflict of Interest

Rice University owns intellectual property on the use of electromagnetic (light) activation of molecular machines for the killing of cells. This intellectual property has been licensed to a company in which J.M.T. is a stockholder, although he is not an officer or director of that company. Conflicts of interest are mitigated by regularly submitting information to the Rice University Office of Sponsored Projects and Research Compliance.

## Supporting information

Supporting Information

## Data Availability

The data that support the findings of this study are available from the corresponding author upon reasonable request.
