# Supplementary material for: Hemithioindigo‐Based Visible Light‐Activated Molecular Machines Kill Bacteria by Oxidative Damage
Source: Adv Sci (Weinh). 2022 Aug 24;9(30):2203242. doi: 10.1002/advs.202203242 (PMC9596824; doi:10.1002/advs.202203242)
Supplement: Supplementary file 1 — Supporting Information [file ADVS-9-2203242-s001.pdf]

ADVANCED SCIENCE
Open Access

Supporting Information

for *Adv. Sci.*, DOI 10.1002/advs.202203242

Hemithioindigo-Based Visible Light-Activated Molecular Machines Kill Bacteria by Oxidative Damage

Ana L. Santos, Alexis van Venrooy, Anna K. Reed, Aaron M. Wyderka, Víctor García-López, Lawrence B. Alemany, Antonio Oliver, George P. Tegos and James M. Tour**

SUPPORTING INFORMATION

Hemithioindigo-Based Visible Light-Activated Molecular Machines Kill Bacteria By Oxidative Damage

Ana L. Santos,[1,2]† * Alexis van Venrooy, [1]† Anna K. Reed,[1] Aaron M. Wyderka,[1] Víctor García-López,[1] Lawrence B. Alemany,[1,3] Antonio Oliver,[2,4] George P. Tegos,[5] James M. Tour[1,6,7,8*]

[1]Department of Chemistry, Rice University, Houston, Texas 77005, USA.
[2]IdISBA - Fundación de Investigación Sanitaria de las Islas Baleares, Palma, Spain
[3]Shared Equipment Authority, Rice University, Houston, Texas 77005, USA.
[4]Servicio de Microbiologia, Hospital Universitari Son Espases, Palma, Spain
[5]Office of Research, Reading Hospital, Tower Health, 420 S. Fifth Avenue, West Reading, Pennsylvania 19611, USA.
[6]Smalley-Curl Institute, Rice University, Houston, Texas 77005, USA.
[7]Department of Materials Science and Nanoengineering, Rice University, Houston, Texas 77005, USA.
[8]NanoCarbon Center and the Welch Institute for Advanced Materials, Rice University, Houston, Texas 77005, USA.

†These authors contributed equally to this work
*Lead corresponding author: tour@rice.edu
Correspondence: alsantos@ua.pt (ORCID ID: 0000-0002-5450-9414), tour@rice.edu

Table of Contents

Supporting Results 3

Supporting Tables 3

Supporting Figures 7

Supporting Materials and Methods 14

A. General synthetic schemes 14

B. Detailed synthetic procedures 16

C. NMR spectra of synthetic intermediates 31

D. Additional characterization of *E* and *Z* isomers of 2 58

Additional Comments on the NMR Analysis of HTI Motors 75

Supporting Results

Supporting Tables

Table S1. List of bacterial strains and mammalian cell lines used in this study, their source, and growth conditions.

**Strains**					
**Bacteria**	**Source**	**Identifier**	**Gram**	**Media**	**Temp.**
Bacillus megaterium	ATCC	ATCC® 14581™	Positive	Nutrient Broth/Agar	30 °C
Bacillus subtilis	ATCC	ATCC® 6051™	Positive	Nutrient Broth/Agar	30 °C
Enterococcus faecalis	ATCC	ATCC® 29212™	Positive	Brain Heart Infusion Broth/Agar	37 °C
Enterococcus faecium	ATCC	ATCC® 19434™	Positive	Brain Heart Infusion Broth/Agar	37 °C
Staphylococcus aureus	ATCC	ATCC® 700698	Positive	Brain Heart Infusion Broth/Agar	37 °C
Staphylococcus epidermidis	ATCC	ATCC® 12228™	Positive	Nutrient Broth/Agar	37 °C
MRSA	ATCC	ATCC® BAA-1680	Positive	Tryptic Soy Broth/Agar	37 °C
Stenotrophomonas maltophilia	ATCC	ATCC® 13637™)	Negative	Nutrient Broth/Agar	30 °C
Acinetobacter baumannii	ATCC	ATCC® BAA-1605™	Negative	Tryptic Soy Broth/Agar	37 °C
Burkholderia cepacia	ATCC	ATCC® 25416™	Negative	Nutrient Broth/Agar	30 °C
Enterobacter cloacae.	ATCC	ATCC® 13047™	Negative	Nutrient Broth/Agar	30 °C
Escherichia coli	G. Bennett (RU)	BW25113	Negative	Nutrient Broth/Agar	37 °C
Pseudomonas aeruginosa	DSMZ	DSM 22644 (PAO1)	Negative	Tryptic Soy Broth/Agar	30 °C
**Mammalian Cell Lines**	**Source**	**Identifier**		**Media**	**Temp.**
Human Embryonic Kidney cells HEK-293	ATCC	ATCC® CRL-1573™	-	Eagle's Minimum Essential Medium (ATCC® 30-2003™)	37 °C
Primary normal human dermal fibroblasts (NHDFs)	ATCC	ATCC® PCS-201-012™	-	Fibroblast Basal Medium (ATCC® PCS-201-030™)	37°C

Table S2. Antibiotic MIC (in µg per mL) of different Gram-positive strains examined in this study. Results are the mean of at least three biological replicates determined using the broth microdilution method.

**Antibiotic**	**Bacterial strain**
	B. megaterium
Ciprofloxacin	0.25
Gentamicin	0.25
	B. subtilis
Ciprofloxacin	0.03
Gentamicin	0.125
	E. faecium
Ciprofloxacin	2
Gentamicin	4
	E. faecalis
Ciprofloxacin	1
Gentamicin	8
	S. epidermidis
Ciprofloxacin	0.125
Gentamicin	0.5
	S. aureus
Ciprofloxacin	1
Gentamicin	2
	MRSA
Ciprofloxacin	8
Vancomycin	0.5

Table S3. Susceptibility (assessed as the MIC) of antibiotic-resistant *B. subtilis* and *S. aureus* to visible light-activated HTI. During serial passaging experiments used to track the development of antibiotic resistance, cells growing at half the MIC for each antibiotic were collected in each passage and stored at -80 °C for further testing of their HTI MIC by irradiating cell suspensions amended with a range of HTI concentrations with 455 nm light at 65 mW cm^{-2} for 10 min, followed by inoculation into MHB and overnight growth, as described in Materials and Methods. The results are the mean of at least three biological replicates.

	MIC (μM)											
	B. subtilis						*S. aureus*					
	Ciprofloxacin^R			Gentamycin^R			Ciprofloxacin^R			Gentamycin^R		
Passage number	HTI **1**	HTI **6**	HTI **7**	HTI **1**	HTI **6**	HTI **7**	HTI **1**	HTI **6**	HTI **7**	HTI **1**	HTI **6**	HTI **7**
1	10	1.25	0.3125	10	1.25	0.3125	10	5	5	10	5	5
2	10	1.25	0.3125	10	1.25	0.3125	10	5	5	10	5	5
3	10	1.25	0.3125	10	1.25	0.3125	10	5	5	10	5	5
4	10	1.25	0.3125	10	1.25	0.3125	10	5	5	10	5	5
5	10	1.25	0.3125	10	1.25	0.3125	10	5	5	10	5	5
6	10	1.25	0.3125	10	1.25	0.3125	10	5	5	10	5	5
7	10	1.25	0.3125	10	1.25	0.3125	10	5	5	10	5	5
8	10	1.25	0.3125	10	1.25	0.3125	10	5	5	10	5	5
9	10	1.25	0.3125	10	1.25	0.3125	10	5	5	10	5	5
10	10	1.25	0.3125	10	1.25	0.3125	10	5	5	10	5	5
11	10	1.25	0.3125	10	1.25	0.3125	10	5	5	10	5	5
12	10	1.25	0.3125	10	1.25	0.3125	10	5	5	10	5	5
13	10	1.25	0.3125	10	1.25	0.3125	10	5	5	10	5	5
14	10	1.25	0.3125	10	1.25	0.3125	10	5	5	10	5	5
15	10	1.25	0.3125	10	1.25	0.3125	10	5	5	10	5	5
16	10	1.25	0.3125	10	1.25	0.3125	10	5	5	10	5	5
17	10	1.25	0.3125	10	1.25	0.3125	10	5	5	10	5	5
18	10	1.25	0.3125	10	1.25	0.3125	10	5	5	10	5	5
19	10	1.25	0.3125	10	1.25	0.3125	10	5	5	10	5	5
20	10	1.25	0.3125	10	1.25	0.3125	10	5	5	10	5	5

Table S4. Statistical significance (p-value and summary of p-value in brackets) of the difference between the survival curves of *G. mellonella* infected with MRSA and treated with a combination of HTI and antibiotic (2x MIC of HTI **1**, **6,** or **7** followed by 10 min of irradiation with 455 nm light at 65 mW cm^{-2} and subsequently treated with 2x MIC of antibiotics ciprofloxacin and vancomycin), HTI alone or antibiotic alone. Statistical analysis of survival curves was performed in GraphPad Prism 8 using the log-rank test (Mantel-Cox). Each survival curve was generated based on the results obtained from three biological replicates, each comprised of ten individuals, totaling 30 data points per treatment. * p <0.05, ** p <0.01, *** p <0.001, **** p <0.0001. ns: not significant.

	**HTI 1 + Ciprofloxacin**	**HTI 6 + Ciprofloxacin**	**HTI 7+ Ciprofloxacin**
**Ciprofloxacin alone**	0.0078 (**)	0.0128 (*)	0.0123 (*)
**HTI 1 alone**	0.0019 (**)	-	-
**HTI 6 alone**	-	0.0029 (**)	-
**HTI 7 alone**	-	-	0.0344 (*)
	**HTI 1 + Vancomycin**	**HTI 6 + Vancomycin**	**HTI 7 + Vancomycin**
**Vancomycin alone**	0.168 (ns)	0.1109 (ns)	0.4264 (ns)
**HTI 1 alone**	0.1886 (ns)	-	-
**HTI 6 alone**	-	0.1009 (ns)	-
**HTI 7 alone**	-	-	0.2417 (ns)

Supporting Figures

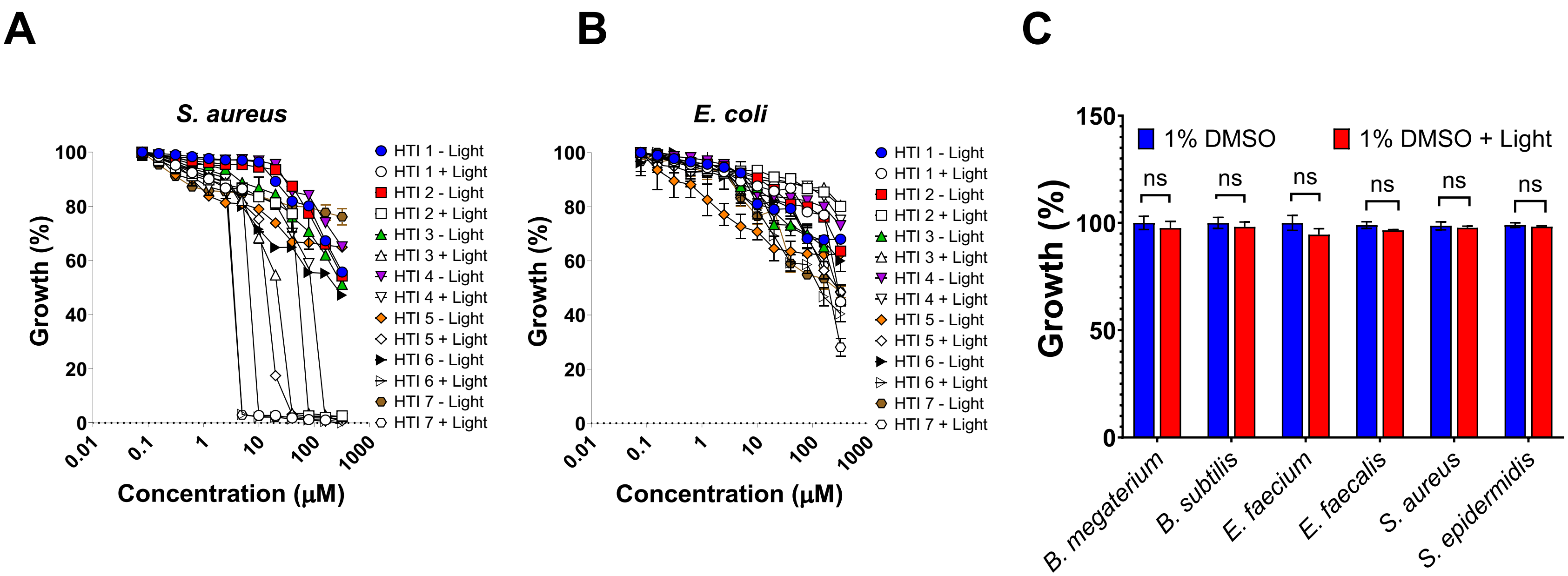

Figure S1. Antibacterial activity of visible light-activated HTI. Light-dependent growth inhibition by increasing concentrations of different HTI in the Gram-positive *S. aureus* (**A**) but not in the Gram-negative *E. coli* (**B**). (**C**) Percentage of growth reduction in different Gram-positive bacterial strains treated with 1% DMSO or 1% DMSO plus light (10 min of irradiation with 455 nm light at 65 mW cm^{-2} corresponding to a light dose of 39 J cm^{-2}). Growth was measured as OD_{600} after inoculation of HTI-treated cells in Mueller-Hinton broth (MHB) and overnight incubation at the appropriate temperature (**Table S1**). Results are expressed as the average of at least three biological replicates ± standard error of the mean.

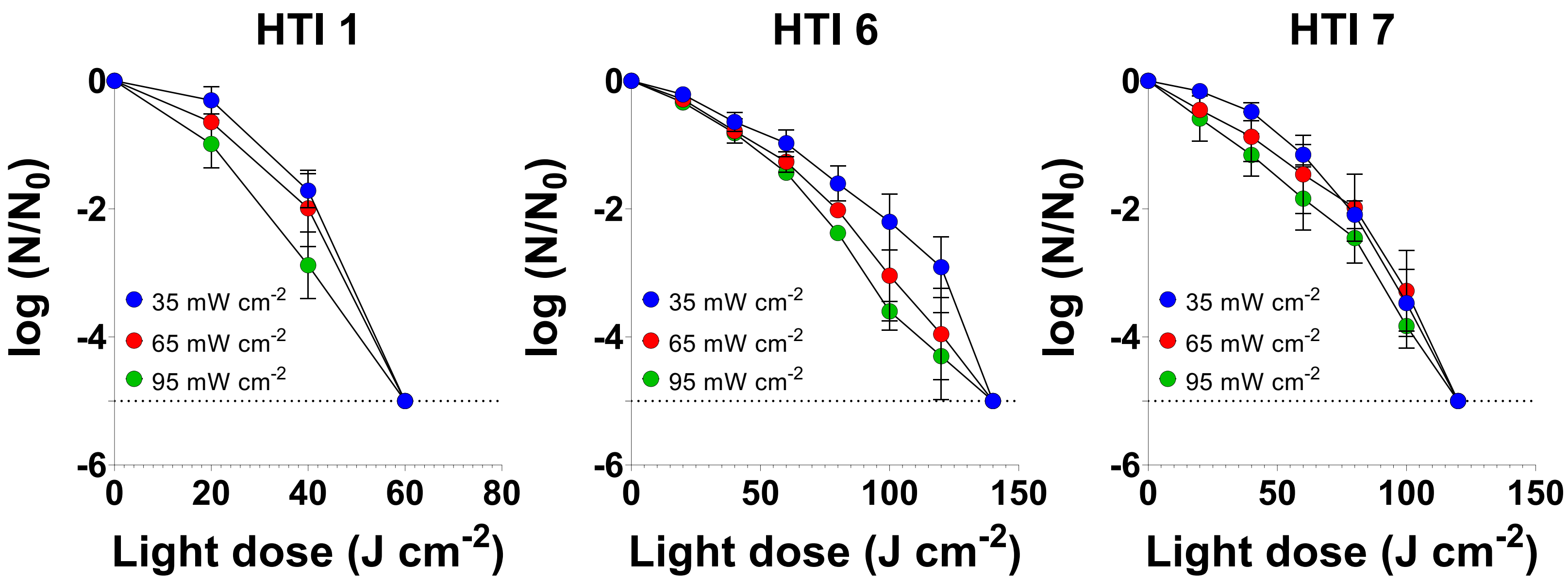

Figure S2. Influence of light dose (fluence) and fluence rate on the killing of exponentially growing *S. aureus* by HTI **1**, **6,** and **7**. Survival is expressed as the logarithm of base 10 of the ratio between the CFU at every sample collection point and the CFU at the beginning of irradiation. The results are shown as the mean of three biological replicates ± standard error of the mean. The dashed line denotes the limit of detection of the method. Further experimental details can be found in the main document.

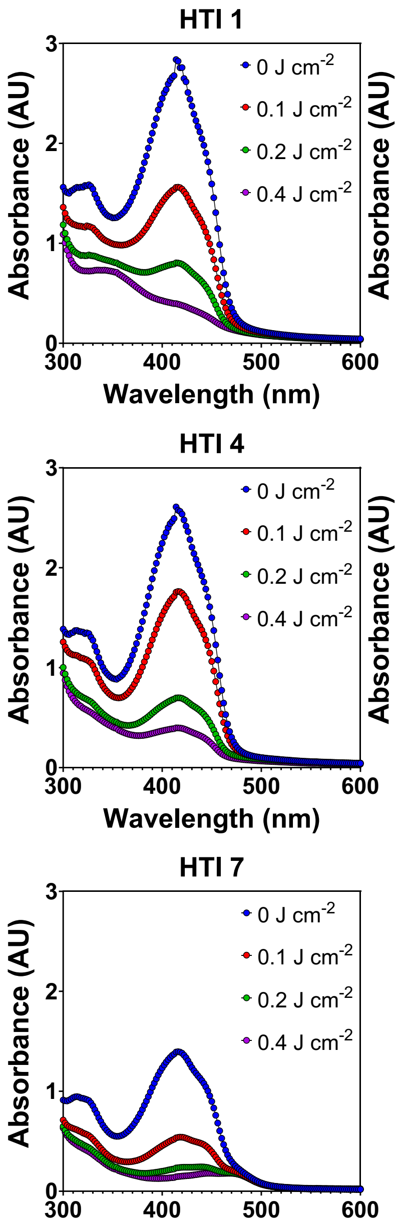

Figure S3. UV-vis spectra of the singlet oxygen trap DPBF in the presence of 0.8 mM of different HTI and increasing doses of 455 nm light. Each curve represents the average of three technical replicates ± standard error of the mean.

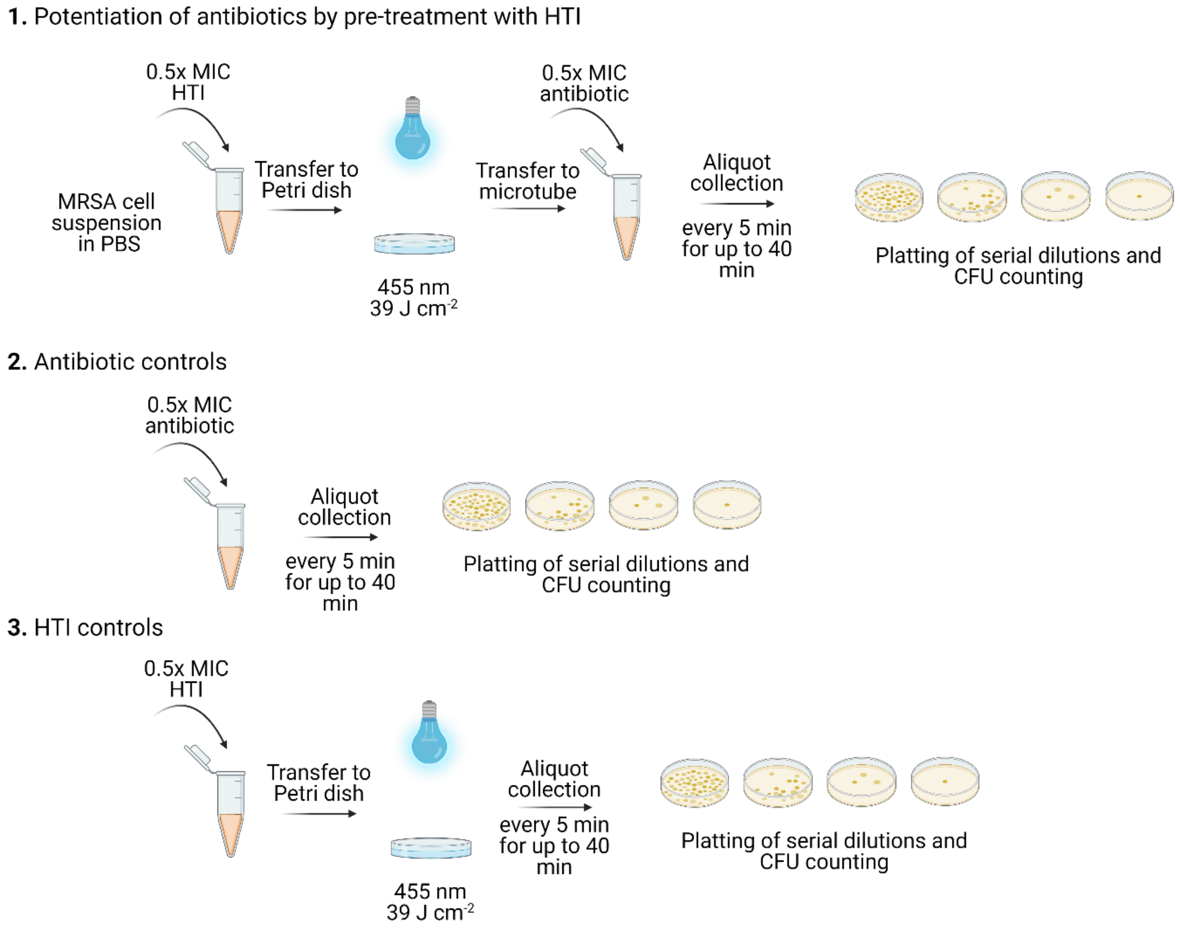

Figure S4. Schematic representation of the experimental setup used to assess the potentiation of antibiotic activity by HTI against MRSA *in vitro*. (*1*) MRSA cell suspensions were challenged with 0.5x MIC of the most potent HTI molecules (HTI **1**, **6**, and **7**), followed by 10 min of irradiation with 455 nm light at 65 mW cm-2 (39 J cm^{-2}). The cells were then treated with 0.5x the MIC of the antibiotics ciprofloxacin or vancomycin. The controls consisted of cells treated with 0.5x MIC of each antibiotic alone (*2*) or 0.5x MIC of HTI alone (*3*). Further experimental details are provided in the main document.

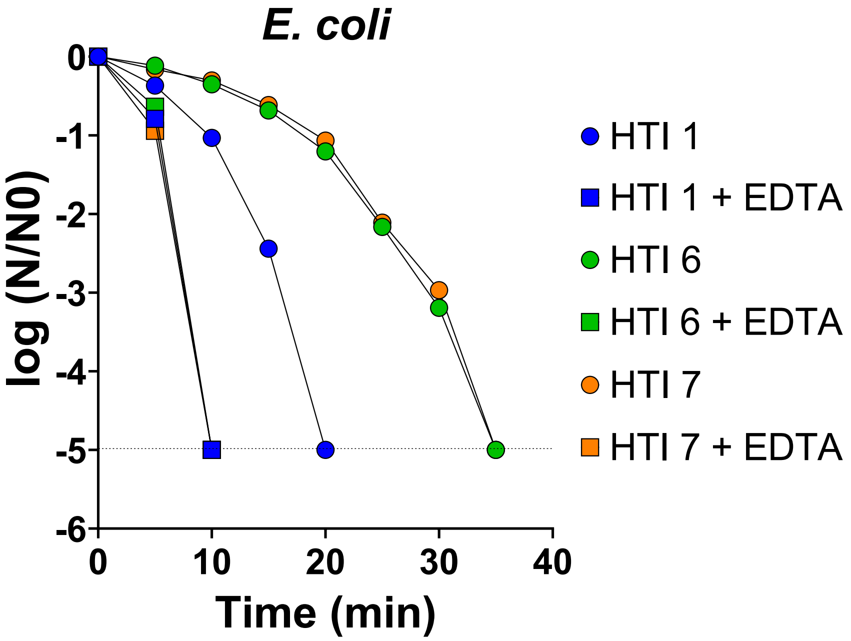

Figure S5. Time-dependent reduction in colony-forming units (expressed as the logarithm of the ratio between the cell number at each time point and the cell number at time zero) of *E. coli* that were untreated or pre-treated with Tris-EDTA (100 mM and 10 mM, respectively, pH 7.2, 60 min) [1] and then challenged with 1 x MIC of each HTI and 455 nm light at 65 mW cm^{-2}. The dashed line denotes the detection limit of the method. All results are expressed as the mean of at least three biological replicates ± standard error of the mean.

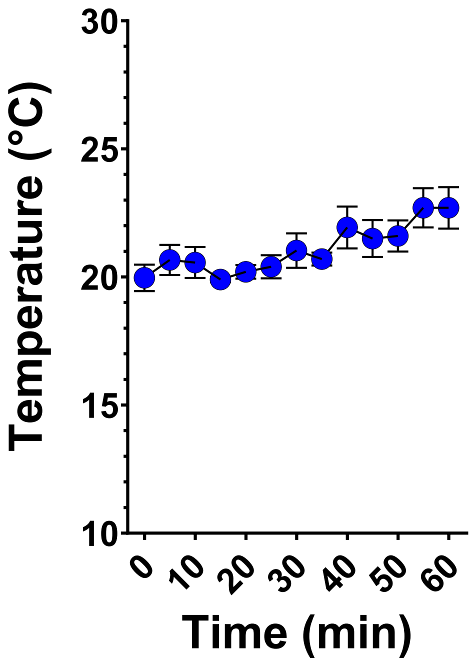

Figure S6. Temperature (°C) during irradiation of samples treated with 320 μM of a representative HTI (HTI **1**) assessed using a temperature probe (Model SC-TT-K-30-36-PP; Omega Engineering, Inc.). The results are the average of three independent experimental measurements. The error bars are the standard error of the mean.

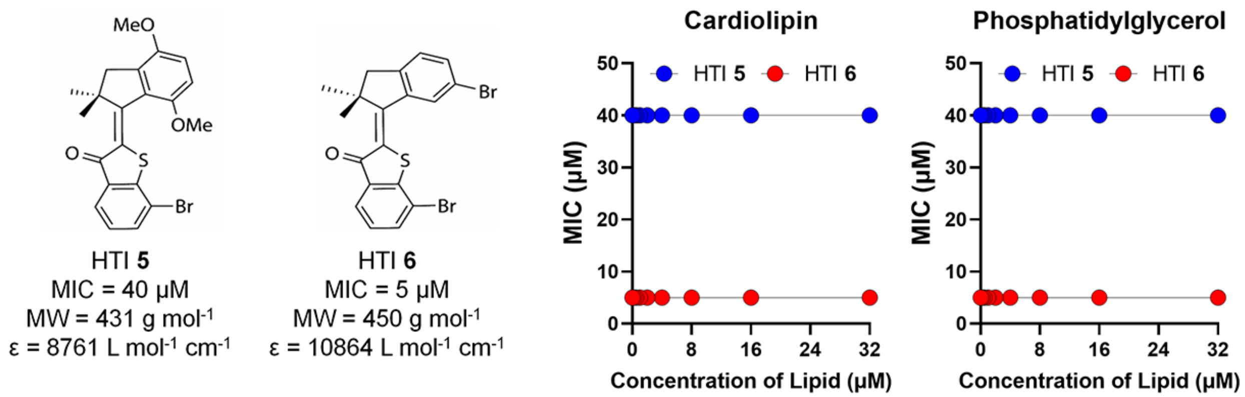

Figure S7. HTI **5** and HTI **6** do not bind to the major membrane phospholipids of *S. aureus*. Binding assays with exogenous phospholipids[2] in *S. aureus* were used to determine whether differences in HTI-bacteria interactions were responsible for the different antibacterial activities of HTI. HTI **5** and HTI **6** were selected because, although HTI **5** (MIC = 40 μM) contains two methoxy groups that should increase its affinity for lipid bilayers, it showed much lower antibacterial activity than HTI **6** (MIC = 5 μM), in which the methoxy groups are not present. The phospholipids cardiolipin and phosphatidylglycerol were used for the competition experiments because they are considered the major membrane phospholipids of *S. aureus*.[3] In these experiments, exogenous phospholipids were added to a cell suspension of *S. aureus* along with HTI before irradiation. If HTI bind the exogenous phospholipids, more HTI is required to kill the cells, which is reflected by an increase in MIC compared with samples without the exogenous compounds. However, the addition of increasing concentrations of phospholipids to the samples did not affect the MIC of HTI, indicating that neither HTI **5** nor HTI **6** physically bind to the membrane of *S. aureus*.

Supporting Materials and Methods

A. General synthetic schemes

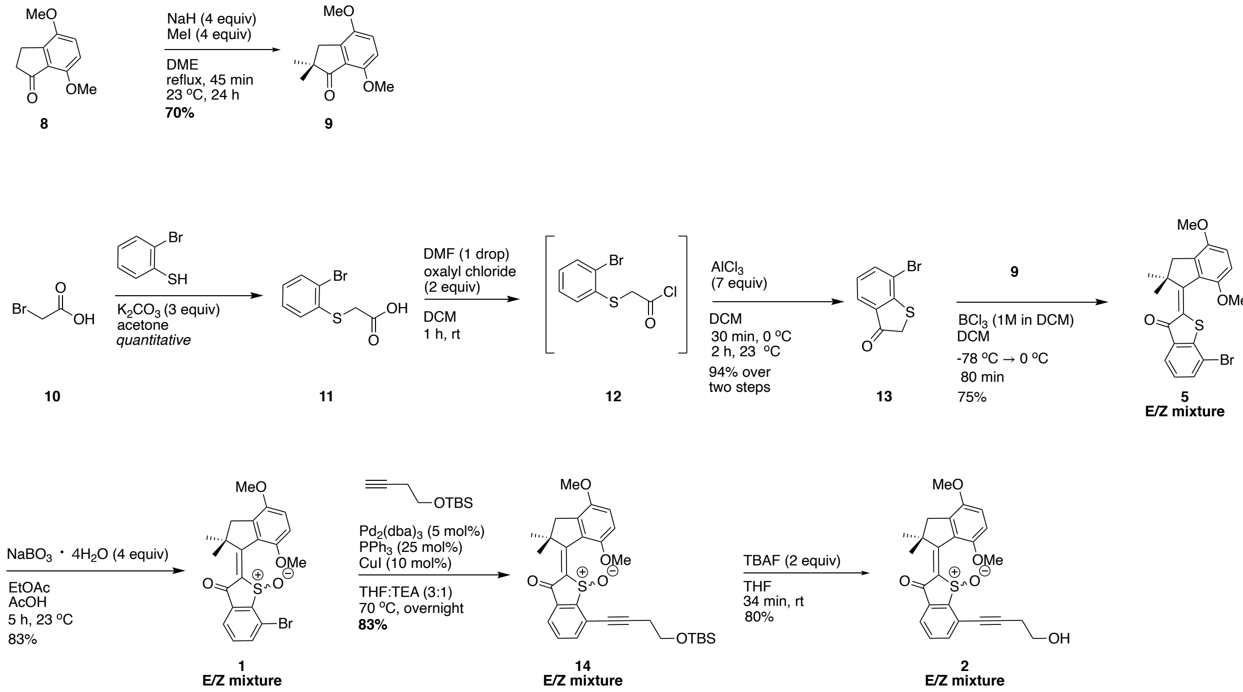

Scheme S1: Synthesis of HTI **1**, **2,** and **5**.

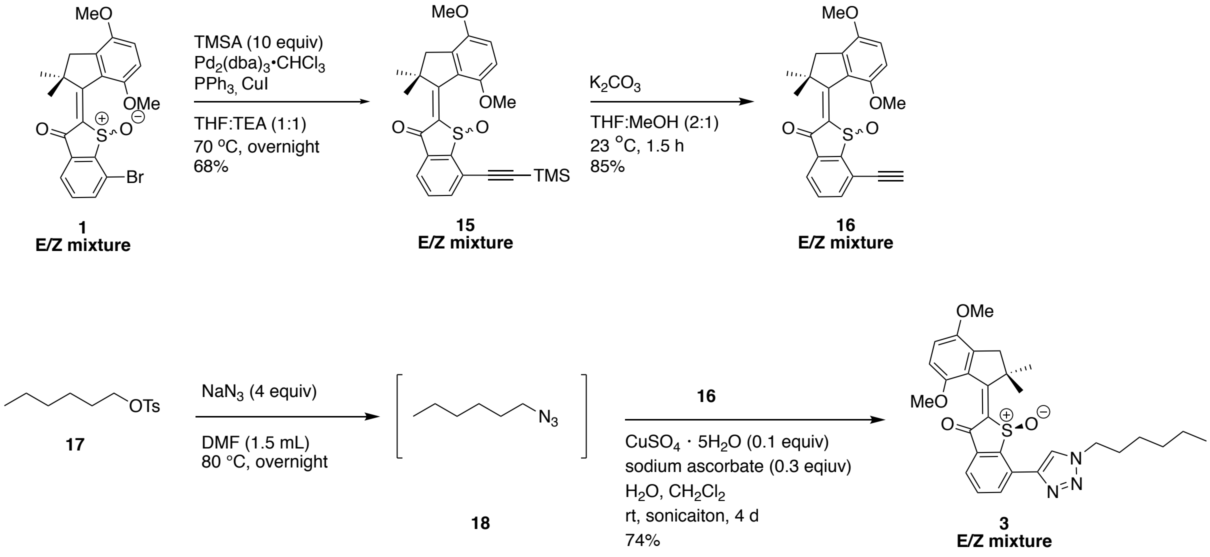

Scheme S2: Synthesis of HTI **3**.

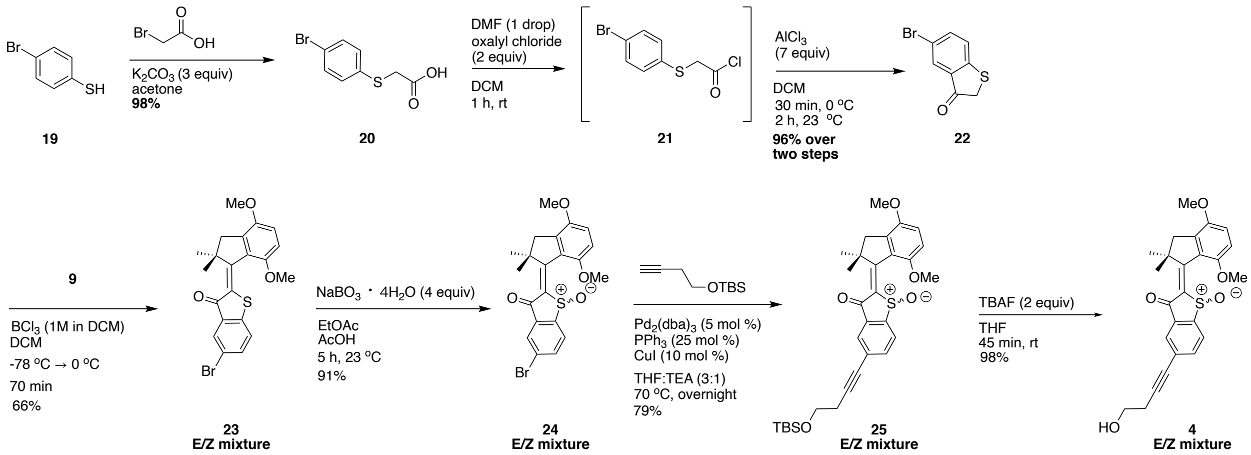

Scheme S3: Synthesis of the HTI **4**.

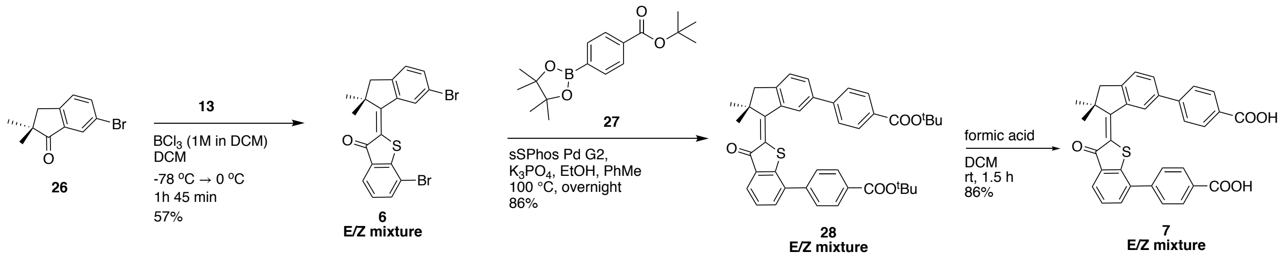

Scheme S4: Synthesis of HTI **6** and **7**.

B. Detailed synthetic procedures

General Synthetic Procedures: All glassware was oven-dried prior to use. Reagent grade tetrahydrofuran (THF) was distilled from sodium benzophenone ketyl, and reagent grade triethylamine (Et_3N) was distilled from calcium hydride. All other chemicals were purchased from commercial suppliers and used without further purification. Flash column chromatography was performed using 230-400 mesh silica gel. Thin-layer chromatography was performed using glass plates pre-coated with silica gel F_{254} 0.25 mm layer thickness purchased from EM Science (Gibbstown, NJ, USA). ^{1}H NMR and ^{13}C NMR were recorded at either 500 MHz and 126 MHz or 600 MHz and 151 MHz. Chemical shifts (δ) are recorded in ppm from trimethylsilane (TMS). All products, except compound **2**, were left as mixtures of *E*/*Z* isomers. For **2**, the isomers were separated with column chromatography in the absence of light and characterized individually. Compounds **9**, **13**, **17**, and **22** are reported compounds and were synthesized *via* previously described methods.[4–6]

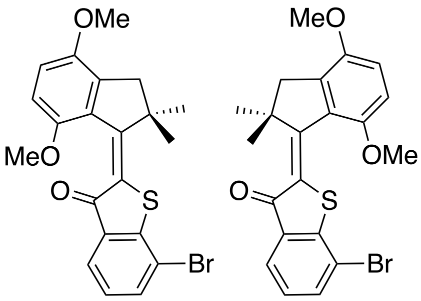

Compound 5. Under an argon atmosphere, compound **13** (117 mg, 0.51 mmol) was dissolved in freshly distilled anhydrous DCM (3 mL). The solution was cooled to -78 °C, and BCl_3 (510 µL, 1.0M in DCM) was added. The solution was quickly cannulated into a second solution, precooled to 0 °C, of compound **9** (70 mg, 0.036 mmol) in 0.8 mL anhydrous DCM. The reaction was stirred

at 0 °C for 80 min. At this point, the reaction was quenched with the addition of saturated aqueous $NaHCO_3$, and the mixture was extracted with DCM three times. After purification with column chromatography (12% EtOAc in hexanes), compound **5** was isolated as a yellow-orange solid (103 mg, 73%). ^{1}H NMR (500 MHz, $CDCl_3$) 7.84 (dd, J_1 = 7.6 Hz, J_2 = 0.9 Hz, 0.82H), 7.74 (dd, J_1 = 7.6 Hz, J_2 = 0.9 Hz, 0.16H), 7.67 (dd, J_1 = 7.6 Hz, J_2 = 0.9 Hz, 0.80H), 7.64 (dd, J_1 = 7.6 Hz, J_2 = 0.9 Hz, 0.18H), 7.16 (app t, J = 7.6 Hz, 0.82H), 7.10 (app t, J = 7.6 Hz, 0.18H), 6.93 (d, J = 8.8 Hz, 0.15H), 6.88 (d, J = 9.1 Hz, 0.83H), 6.78 (d, J = 8.8 Hz, 0.16H), 6.72 (d, J = 9.0 Hz, 0.83H), 3.96 (s, 0.44H), 3.82 (s, 0.48H), 3.81 (s, 2.30H), 3.79 (s, 2.55H), 3.02 (d, J = 16.2 Hz, 0.80H), 2.93 (s, 0.36H), 2.87 (d, J = 15.7 Hz, 0.77H) 1.73 (s, 2.33H), 1.56 (s, 0.95H), 1.27 (s, 2.39H). ^{13}C NMR (126 MHz, $CDCl_3$) δ 188.63, 186.55, 164.19, 158.41, 152.84, 150.51, 149.84, 149.57, 147.25, 145.26, 137.91, 136.58, 136.54, 136.09, 134.36, 134.21, 129.33, 128.87, 128.71, 125.99, 125.87, 125.60, 124.60, 124.39, 117.38, 116.77, 115.01, 114.28, 110.10, 109.57, 56.07, 55.91, 55.39, 54.95, 51.61, 50.80, 47.73, 45.90, 26.63, 26.49, 26.22. HRMS (ESI) *m/z* calculated for [M+H$^+$] $C_{21}H_{19}BrO_3S$ 431.0311; found 431.0291.

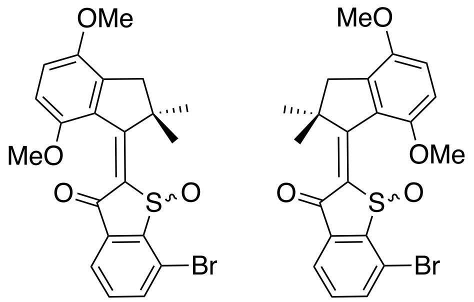

Compound 1. Compound **5** (494 mg, 1.15 mmol) was dissolved in 115 mL AcOH and 9 mL of EtOAc. Sodium perborate tetrahydrate (763 mg, 4.99 mmol) was added, and the reaction was stirred at room temperature for 5 h. $NaHCO_3$ was added, and the aqueous layer was extracted with

EtOAc three times. The combined organic layers were dried over Na_2SO_4, and the solvent was removed under vacuum. The product was purified with column chromatography (2.5% Acetone in DCM) to give **1** as yellow-orange solid (430 mg, 83%). ^{1}H NMR (500 MHz, $CDCl_3$) δ 7.96 (d, J = 7.9 Hz, 0.32H), 7.92 (dd, J_1 = 7.6 Hz, J_2 = 1.1 Hz, 0.69 H), 7.90 (dd, J_1 = 7.8 Hz, J_2 = 1.0 Hz, 0.29H), 7.88 (dd J_1 = 7.9 Hz, J_2 = 1.0 Hz, 0.64H), 7.57 (app t, J = 7.7 Hz, 0.34H) 7.52 (appt t, J = 7.7 Hz, 0.64H), 7.01 (d, J_1 = 8.9 Hz, 0.66 H), 6.97 (d, J = 8.9 Hz, 0.32H), 6.81 (d, J = 8.8 Hz, 0.66 H), 6.73 (d, J = 8.8 Hz, 0.31H), 4.08 (s, 1.98H), 3.82 (s, 0.98H), 3.82 (s, 1.88H), 3.75 (s, 0.89H), 3.07 (d, J = 15.2 Hz, 0.30H), 3.02 (d, J = 16.6 Hz, 0.69H), 2.93 (set of overlapping doublets, measured one set J = 16.2 Hz, other set unable to measure J val, 0.92H total), 1.97 (s, 0.95H), 1.56 (s, 3.99H), 1.44 (s, 0.97H). ^{13}C NMR (151 MHz, $CDCl_3$) δ 183.97, 181.80, 172.72, 171.41, 153.35, 151.44, 149.88, 149.60, 147.93, 146.74, 141.38, 139.12, 138.79, 138.36, 138.32, 138.23, 137.84, 133.84, 133.18, 128.81, 126.47, 124.08, 123.71, 122.73, 122.23, 116.74, 116.20, 109.72, 109.37, 56.05, 55.95, 55.35, 55.07, 52.91, 52.40, 47.27, 46.46, 28.47, 28.25, 26.21, 25.36. HRMS (ESI) *m/z* calculated for [M+H$^+$] $C_{21}H_{19}BrO_4S$ 447.0260, found, 447.0248

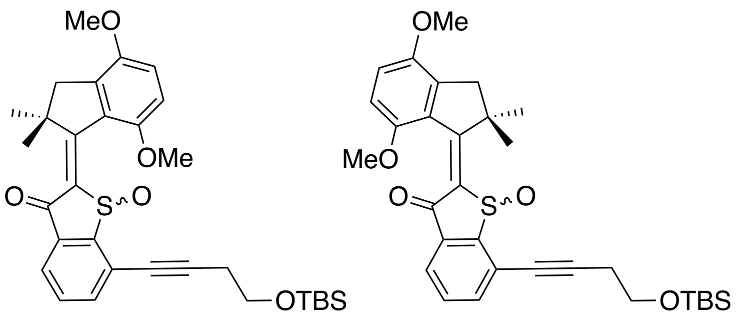

Compound 14. An oven-dried screw cap tube was charged with Compound **1** (158 mg, 0.35 mmol). Under Ar atmosphere, $Pd_2(dba)_3$•$CHCl_3$ (16 mg, 0.02 mmol, 5 mol%), PPh_3 (23 mg, 0.09 mmol, 25 mol%), and CuI (7 mg, 0.04 mmol, 10 mol%) were added. The tube was evacuated and

purged with Ar three times. A 3:1 mixture of dry THF:Et_3N, degassed by bubbling with Ar for 30 min, was then added to the reaction tube. 4-(*tert*-butyldimethylsilyloxy)-1-butyne (186 μL, 4.44 mmol) was added, and the reaction was stirred overnight at 70 °C. The reaction was quenched with a saturated solution of NH_4Cl and extracted with CH_2Cl_2 three times. The product was purified with column chromatography (40% EtOAc in hexanes) to afford **14** as a yellow solid (164 mg, mixture of isomers, 85%). ^{1}H NMR (500 MHz, $CDCl_3$) 7.89 (d, J = 7.4 Hz, 0.36H), 7.85 (dd, J_1 = 8.1 Hz, J_2 = 0.7 Hz, 0.63H), 7.76 (dd, J_1 = 7.8 Hz, J_2 = 0.9 Hz, 0.34H), 7.73 (dd, J_1 = 7.5 Hz, J_2 = 0.8 Hz, 0.60H), 7.62 (app t, J = 7.9 Hz, 0.36H), 7.57 (app t, J = 7.9 Hz, 0.61H), 7.00 (d, J = 9.1 Hz, 0.59H), 6.96 (d, J = 8.7 Hz, 0.38H), 6.82 (d, J = 9.1 Hz, 0.66H), 6.72 (d, J = 8.7 Hz, 0.43H), 4.07 (s, 1.71H), 3.91 (td, J_1 = 7.0 Hz, J_2 = 1.3 Hz, 0.67H), 3.86 (t, J = 7.9 Hz, 1.13H), 3.83 (s, 0.98H), 3.82 (s, 1.64H), 3.75 (s, 0.93H), 3.07 (d, J = 15.0 Hz, 0.33H), 3.00 (d, J = 16.6 Hz, 0.66H), 2.91 (set of overlapping doublets, measured one set J = 16.6 Hz, other set unable to measure J val, 0.97H total), 2.80 (t, J = 6.9 Hz, 0.72H) 2.73 (t, J = 6.7 Hz, 1.24H), 1.97 (s, 0.96H), 1.55 (s, 4.06H), 1.44 (s, 1.06H), 0.93 (s, 3.04H), 0.91 (s, 5.71H), 0.12 (s, 1.88H), 0.09 (s, 3.85) ppm. ^{13}C NMR (151 MHz, $CDCl_3$) δ 184.76, 182.63, 171.61, 170.18, 153.26, 151.37, 150.23, 149.88, 149.61, 148.64, 142.30, 138.90, 138.69, 138.50, 138.16, 137.62, 136.41, 136.20, 132.25, 131.63, 128.91, 126.74, 124.27, 123.91, 123.87, 123.55, 116.44, 115.85, 109.73, 109.48, 97.24, 96.76, 76.34, 76.20, 61.65, 61.60, 56.05, 55.98, 55.36, 54.96, 52.72, 52.35, 47.21, 46.41, 28.44, 28.26, 25.91, 25.89, 25.39, 24.23, 24.17, 18.36, 18.32, -5.22. HRMS (ESI) *m/z* calculated for [M+H$^+$] $C_{31}H_{38}O_5SSi$ 551.2282; found, 551.2261

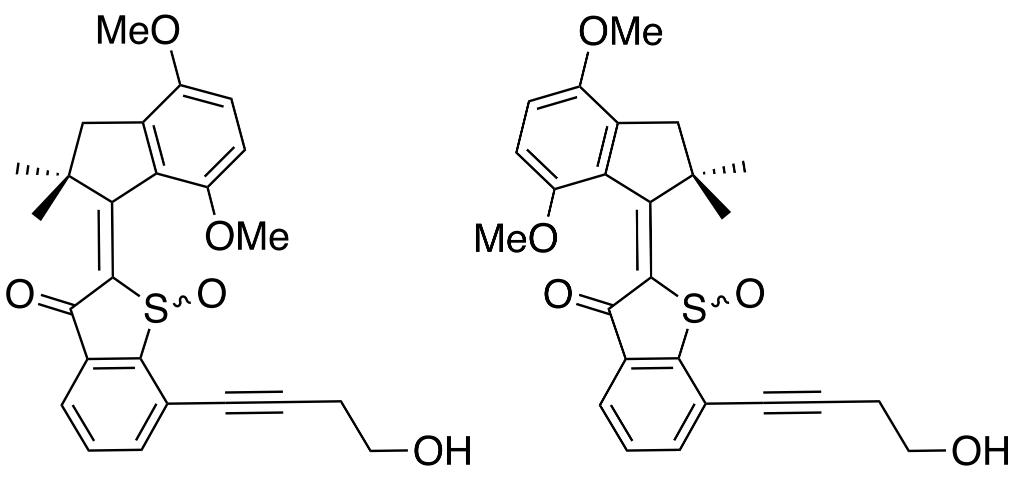

Compound 2. Compound **14** (164 mg, 0.30 mmol) was dissolved in 4 mL THF. Tetrabutylammonium fluoride (632 µL, 1.0 M in THF) was added, and the reaction was stirred for 34 min at room temperature. The reaction was then quenched with the addition of water and extracted with ethyl acetate. The product was purified with column chromatography (5%-10% acetone in CH_2Cl_2) to afford **2** as a golden yellow solid (104 mg, mixture of isomers, 80%). HRMS (ESI) *m/z* calculated for [M+H$^+$] $C_{25}H_{34}O_5S$ 437.1417; found, 437.1399. For characterization purposes, the *E* and *Z* isomers were separated with column chromatography in the absence of light. See page **S56** for structure assignments and absorption of the *E* and *Z* isomers.

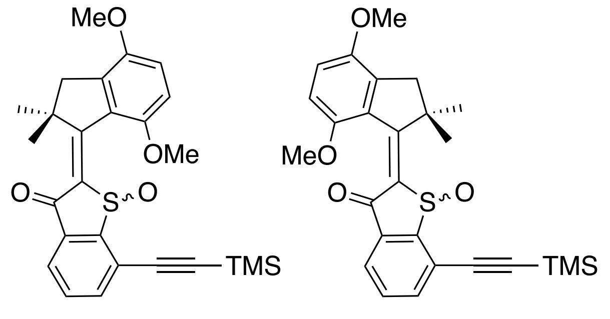

Compound 15. An oven-dried screw cap tube was charged with Compound **1** (50 mg, 0.11 mmol). Under Ar atmosphere, $Pd_2(dba)_3$•$CHCl_3$ (3 mg, 0.003 mmol, 2.5 mol %) PPh_3 (4 mg, 0.015 mmol, 14 mol %) and CuI (1 mg, 0.005 mmol, 5 mol %) were added. The tube was evacuated and purged

with Ar three times. A 1:1 mixture of dry THF:Et_3N, degassed by bubbling with Ar for 30 min, was then added to the reaction tube. Trimethylsilylacetylene (170 μL, 1.20 mmol) was added, the tube was sealed, and the reaction was stirred overnight at 70 °C. The reaction was quenched with a saturated solution of NH_4Cl and extracted with CH_2Cl_2 three times. The product was purified with column chromatography (10%-33% ethyl acetate in hexanes) to afford **15** (35 mg, mixture of isomers, 68%). ^{1}H NMR (500 MHz, $CDCl_3$) 7.92 (dd, J_1 = 7.7 Hz, J_2 = 1.2 Hz, 0.27H), 7.88 (dd, J_1 = 7.7 Hz, J_2 = 1.2 Hz, 0.76H), 7.81 (dd, J_1 = 7.5 Hz, J_2 = 1.2 Hz, 0.26H), 7.78 (dd, J_1 = 7.5 Hz, J_2 = 1.2 Hz, 0.73H), 7.62 (app t, J = 7.7 Hz, 0.31H), 7.58 (app t, J = 7.6 Hz, 0.74H), 7.01 (d, J = 8.8 Hz, 0.77H), 6.95 (d, J = 8.8 Hz, 0.26H), 6.83 (d, J = 8.8 Hz, 0.76H), 6.72 (d, J = 8.8 Hz, 0.24H), 4.08 (s, 2.40H), 3.82 (s, 0.97H), 3.81 (s, 2.10H), 3.72 (s, 0.68H), 3.06 (d, J = 15.0 Hz, 0.23H), 2.98 (d, J = 16.1 Hz, 0.80H), 2.91 (set of overlapping doublets, measured one set J = 16.7 Hz, other set unable to measure J val, 1.01H total), 1.96 (br s, 0.68H), 1.56 (s, 4.80H), 1.45 (s, 0.73H), 0.33 (s, 2.30H), 0.28 (s, 7.14H) ppm. ^{13}C NMR (151 MHz, $CDCl_3$) δ 185.10, 182.96, 172.25, 170.63, 153.67, 151.78, 151.44, 150.28, 149.99, 142.50, 139.37, 138.63, 138.49, 137.98, 136.83, 136.54, 132.54, 131.98, 129.25, 128.58, 126.99, 124.97, 124.60, 123.50, 116.93, 116.20, 110.14, 109.88, 105.18, 104.96, 99.23, 99.12, 56.41, 56.34, 55.76, 55.03, 53.09, 52.55, 47.54, 46.73, 28.88, 28.55, 25.81, 25.77, 0.35, 0.10. HRMS (ESI) *m/z* calculated for [M+H$^+$] $C_{26}H_{28}O_4SSi$ 465.1550; found, 465.1540

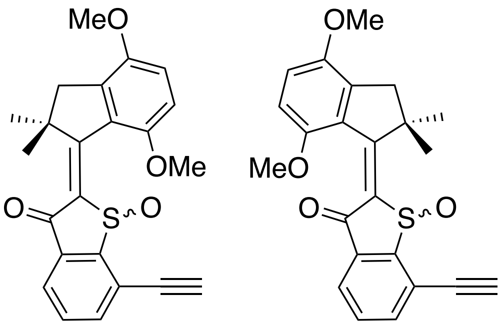

Compound 16. K_2CO_3 (1 mg, 0.01 mmol) was added to a solution of compound **15** (35 mg, 0.08 mmol) in MeOH:THF (2:1, 3 mL). The reaction was stirred at room temperature for 2 hours and subsequently quenched with a saturated solution of $NaHCO_3$ (50 mL). The mixture was then extracted with ethyl acetate (3x 25mL), dried over sodium sulfate, concentrated under vacuum, and purified with column chromatography (10% Acetone in CH_2Cl_2) to afford **16** as an orange solid (25 mg, 85% yield). ^{1}H NMR (500 MHz, $CDCl_3$) 7.98 (dd, J_1 = 7.7 Hz, J_2 = 1.0 Hz, 0.16 H), 7.93 (dd, J_1 = 7.7 Hz, J_2 = 1.0 Hz, 0.86H), 7.88- 7.84 (m, 1H, set of overlapping signals), 7.66 (app t, *J*= 7.5 Hz, 0.15 H), 7.62 (app t, *J*= 7.5 Hz, 0.85 H) 7.00 (d, *J* = 9.0 Hz, 0.85H), 6.96 (d, *J* = 9.0 Hz, 0.14H), 6.81 (d, *J* = 9.0 Hz, 0.88H), 6.72 (d, *J* = 9.0 Hz, 0.11H), 4.07 (s, 2.61H), 3.82 (s, 0.51H), 3.82 (s, 2.46H), 3.75 (s, 0.33H), 3.66 (s, 0.14H), 3.58 (s, 0.81H), 3.08 (d, *J* = 14.7 Hz, 0.17H), 3.02 (d, *J* = 16.5 Hz, 0.89H), 2.91 (set of overlapping doublets, measured one set *J* = 16.4 Hz, other set unable to measure *J* val, 0.96H total), 1.98 (br s, 0.40H), 1.56 (set of two overlapping singlets, 5.35H), 1.44 (br s, 0.37H). ^{13}C NMR (151 MHz, $CDCl_3$) δ 184.28, 182.18, 172.02, 170.79, 153.24, 151.41, 150.97, 149.83, 149.59, 141.82, 139.06, 138.94, 138.48, 137.73, 136.47, 136.33, 132.34, 131.69, 128.79, 126.52, 125.10, 124.71, 122.37, 122.04, 116.53, 116.02, 109.70, 109.36, 86.27, 86.07, 77.85, 77.82, 56.04, 55.94, 55.34, 55.06, 52.80, 52.34, 47.22, 46.45, 28.42, 28.34, 26.16, 25.32. HRMS (ESI) *m/z* calculated for [M+H$^+$] $C_{23}H_{20}O_4S$ 393.1155; found 393.1148.

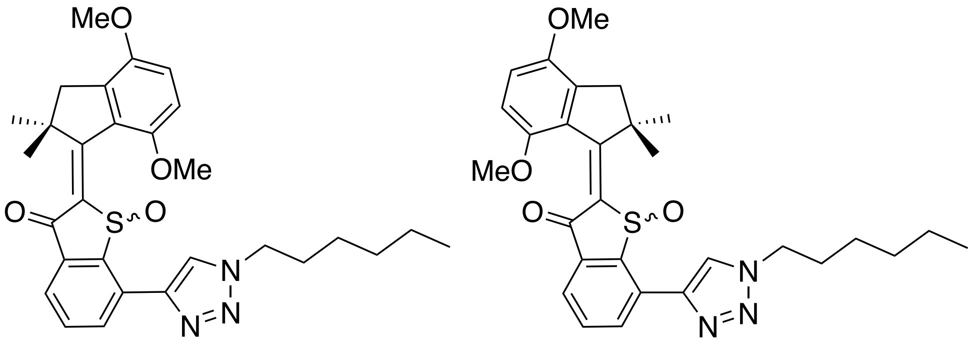

Compound 3. Hexyl 4-methylbenzenesulfonate (208 mg, 0.8 mmol) and sodium azide (202 mg, 3.1 mmol) were added to a screw cap tube. DMF (6 mL) was then added, the tube was sealed, and the reaction was left to stir at 80 ºC overnight. The reaction was subsequently cooled to room temperature, diluted with ether, and washed with 10% HCl (3x 50 mL). The organic layer was concentrated under vacuum, and crude **18** was used directly in the subsequent step. **18** was added into a screw cap tube loaded with motor **16** (99 mg, 0.25 mmol), $CuSO_4$ (3 mg, 0.02 mmol), and sodium ascorbate (12 mg, 0.06 mmol). The tube was evacuated and filled with Ar three times. Water (0.6 mL) and CH_2Cl_2 (0.6 mL) were added into the tube. The reaction was sealed and sonicated at room temperature for 4 days. The reaction was diluted with brine and extracted three times with CH_2Cl_2. The material was purified with column chromatography (gradient 100% CH_2Cl_2 – 5% Acetone 95% CH_2Cl_2) to afford **3** as a yellow-orange solid (95.4 mg, mixture of isomers, 74%). ^{1}H NMR (600 MHz, $CDCl_3$) δ 8.71 (s, 0.88H), 8.59 (dd, J_1 = 7.8 Hz, J_2 = 1.0 Hz, overlaps with signal at 8.58 ppm, 0.92H total), 8.58 (br s, overlaps with signal at 8.59 ppm, 0.92H total), 8.01 (d, J = 7.3 Hz, 0.27H), 7.99 (dd, J_1 = 7.5 Hz, J_2 = 1.1 Hz, 0.66H), 7.81 (app t, J= 7.6 Hz, overlaps with signal at 7.78 ppm, 0.98H total), 7.78 (app t, J= 7.6 Hz, overlaps with signal at 7.81 ppm, 0.98H total) 7.03 (d, J = 9.1 Hz, 0.64H), 6.98 (d, J = 9.1 Hz, 0.31H), 6.83 (d, J = 9.1 Hz, 0.66H), 6.75 (d, J = 9.1 Hz, 0.30H), 4.53- 4.45 (m, 0.64H), 4.45-4.38 (m, 1.28H), 3.99 (s, 1.96H), 3.83 (s overlaps with additional s, total 2.86H), 3.79 (s, 0.84H), 3.09-3.04 (m, 0.96H),

2.97-2.92 (m, 0.99H), 2.06-1.87 (m, 2.93H, set of overlapping signals), 1.63 (s, 1.95H), 1.59 (s, 1.96H),1.46 (br s, 0.77H), 1.39-1.28 (m, 6.38H), 0.91-0.86 (m, 3.31H, set of overlapping signals) ppm. ^{13}C NMR (151 MHz, $CDCl_3$) δ 184.41, 182.37, 172.18, 170.45, 153.49, 151.19, 150.03, 149.60, 144.85, 143.39, 142.61, 142.45, 140.48, 139.20, 137.63, 136.71, 136.59, 136.36, 134.80, 134.33, 132.99, 132.44, 131.95, 128.88, 126.71, 125.87, 125.28, 124.71, 124.36, 116.70, 116.12, 109.76, 109.44, 56.07, 56.01, 55.37, 54.84, 52.79, 52.71, 50.64, 50.54, 47.31, 46.38, 31.18, 30.41, 30.39, 28.52, 28.20, 26.18, 26.11, 25.36, 22.47, 22.41, 14.14, 13.97. HRMS (ESI) *m/z* calculated for [M+H$^+$] $C_{29}H_{33}N_3O_4S$ 520.2265; found 520.2245.

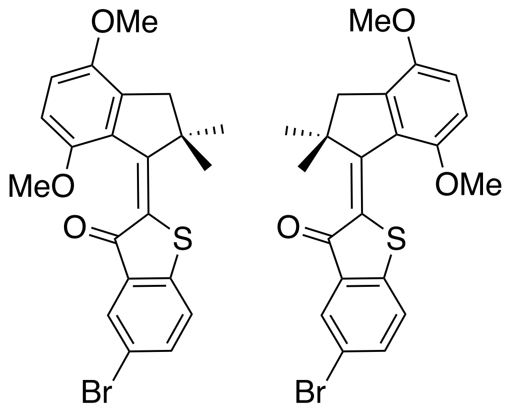

Compound 23. Under argon atmosphere, compound **22** (146 mg, 0.64 mmol) was dissolved in freshly distilled anhydrous DCM (3.8 mL). The solution was cooled to -78 °C, and BCl_3 (640 µL, 1.0 M in DCM) was added. The solution was quickly cannulated into a second solution, precooled to 0 °C, of compound **9** (110 mg, 0.50 mmol) in 0.8 mL anhydrous DCM. The reaction was stirred at 0 °C for 1 h 20 min. At this point, the reaction was quenched with the addition of saturated aqueous $NaHCO_3$, and the mixture was extracted with DCM three times. After purification with column chromatography (25%-50% DCM in hexanes), compound **23** was isolated as a yellow-orange solid (143 mg, mixture of isomers, 66%).^{1}H NMR (500 MHz, $CDCl_3$) δ 7.99 (d, J = 2.0 Hz, 0.62H), 7.89 (d, J = 2.0 Hz, 0.36H), 7.58 (dd, J_1 = 8.4 Hz, J_2 = 1.7 Hz, 0.61H), 7.55 (dd, J_1 =

8.1 Hz, J_2 = 2.0 Hz, 0.38H), 7.32 (d, J = 8.4 Hz, 0.63H), 7.23 (d, J = 8.4 Hz, 0.36H), 6.92 (d, J = 9.0 Hz, 0.36H), 6.88 (d, J = 9.0 Hz, 0.61 H), 6.74, 6.72 (d, d, set of overlapping signals, J = 9.0 Hz for both sets, 0.99H total) 3.90 (s, 1.06H), 3.81, 3.81, 3.80 (s, s, s, set of overlapping signals, 4.83H total), 2.99 (d, J=15.5 Hz, 0.60H), 2.93 (s, 0.73H), 2.86 (d, J = 15.6 Hz, 0.59H), 1.67 (s, 2.03H), 1.55 (s, 2.18H), 1.23 (s, 1.82H) ppm. ^{13}C NMR (126 MHz, $CDCl_3$) δ 187.66, 185.44, 163.91, 158.08, 152.82, 150.35, 149.87, 149.56, 144.87, 142.47, 137.91, 136.61, 136.45, 136.22, 133.88, 133.81, 129.26, 128.84, 128.77, 128.52, 128.29, 125.32, 124.64, 124.20, 118.40, 117.86, 114.93, 114.27, 110.03, 109.55, 56.05, 55.89, 55.35, 54.99, 51.59, 50.70, 47.74, 45.85, 26.50, 26.22. HRMS (ESI) *m/z* calculated for [M+H$^+$] $C_{21}H_{19}BrO_3S$ 431.0311; found 431.0303.

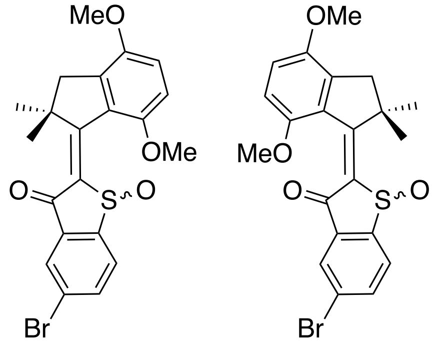

Compound 24. Compound **23** (116 mg, 0.27 mmol) was dissolved in 27 mL AcOH and 2 mL of EtOAc. Sodium perborate tetrahydrate (154 mg, 1.0 mmol) was added, and the reaction was stirred at room temperature for 5 h. $NaHCO_3$ was added, and the aqueous layer was extracted with EtOAc three times. The combined organic layers were dried over Na_2SO_4, and the solvent was removed under vacuum. The product was purified with column chromatography (gradient 20-60% EtOAc in hexanes) to give **24** as yellow-orange solid (109 mg, mixture of isomers, 91%). *E* isomer: ^{1}H NMR (500 MHz, $CDCl_3$) δ 8.13 (d, J = 1.7 Hz, 1.03H), 7.95- 7.91 (m, 2.04H), 6.97 (d, J = 8.9 Hz, 1.03H), 6.73 (d, J = 8.6 Hz, 0.99H), 3.83 (s, 3.08H), 3.76 (s, 3.03H), 3.09 (d, J = 14.9 Hz, 1.03H),

2.93 (d, $J = 14.9$ Hz, 1.02H), 1.96 (s, 3.03H), 1.43 (s, 2.78H) ppm. ^{13}C NMR (126 MHz, $CDCl_3$) δ 181.28, 171.19, 153.23, 149.62, 147.38, 139.07, 137.78, 137.52, 137.21, 128.67, 128.37, 127.92, 127.29, 116.22, 109.70, 56.07, 55.34, 52.71, 46.44, 28.64, 26.07. HRMS (ESI) *m/z* calculated for [M+H$^+$] $C_{21}H_{19}BrO_4S$ 447.0260; found 447.0254.

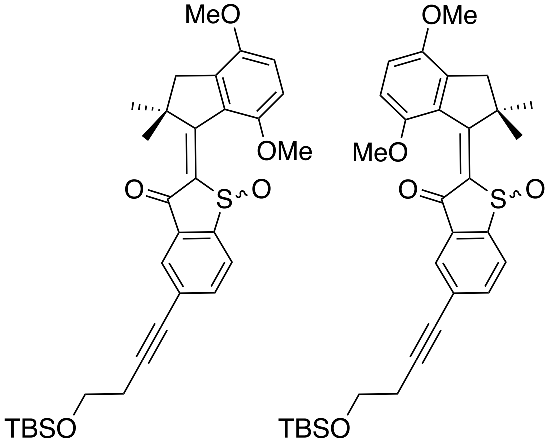

Compound 25. An oven-dried screw cap tube was charged with Compound **24** (64 mg, 0.14 mmol). Under Ar atmosphere, $Pd_2(dba)_3$•$CHCl_3$ (9 mg, 0.01 mmol, 5 mol%), PPh_3 (12 mg, 0.05 mmol, 25 mol%), and CuI (4 mg, 0.02 mmol, 10 mol%) were added. The tube was evacuated and purged with Ar three times. A 3:1 mixture of dry THF:Et_3N (3 mL), degassed by bubbling with Ar for 30 min, was then added to the reaction tube. 4-(*tert*-butyldimethylsilyloxy)-1-butyne (80 µL, 0.48 mmol) was added, and the reaction was stirred overnight at 70 °C. The reaction was quenched with a saturated solution of NH_4Cl and extracted with CH_2Cl_2 three times. The product was purified with two rounds of column chromatography (first: gradient 20-60% EtOAc in hexanes; second: 50% DCM in hexanes, followed by 5% acetone in DCM) to afford **25** as a yellow solid (62 mg, mixture of isomers, 79%). *Z* isomer: ^{1}H NMR (500 MHz, $CDCl_3$) δ 7.93 (d, $J = 0.91$ Hz, 0.98H), 7.91 (dd, $J_1 = 8.1$ Hz, $J_2 = 0.7$ Hz, 1.04H), 7.76 (dd, $J_1 = 7.8$ Hz, $J_2 = 1.2$ Hz, 1.07H),

7.00 (d, J = 9.0 Hz, 1.04H), 6.80 (d, J = 9.0 Hz, 1.01H), 4.04 (s, 3.12H), 3.84 (t, J = 6.7 Hz, 2.18H), 3.81 (s, 3.06H), 2.99 (d, J = 16.4 Hz, 1.00H), 2.90 (d, J = 16.4 Hz, 1.09H), 2.66 (t, J = 7.1 Hz, 2.19H), 1.56 (s, 2.89H), 1.53 (s, 3.24H), 0.92 (s, 9.74H), 0.11 (s, 6.38H) ppm. ^{13}C NMR (126 MHz, $CDCl_3$) δ 184.14, 171.25, 151.36, 149.96, 148.84, 142.50, 138.96, 137.82, 135.78, 127.81, 127.22, 126.24, 126.20, 116.58, 109.35, 91.36, 80.14, 61.61, 55.95, 54.96, 52.18, 47.19, 28.41, 25.89, 25.24, 23.90, 18.36. HRMS (ESI) *m/z* calculated for [M+H$^+$] $C_{31}H_{38}O_5SSi$ 551.2282; found 551.2275.

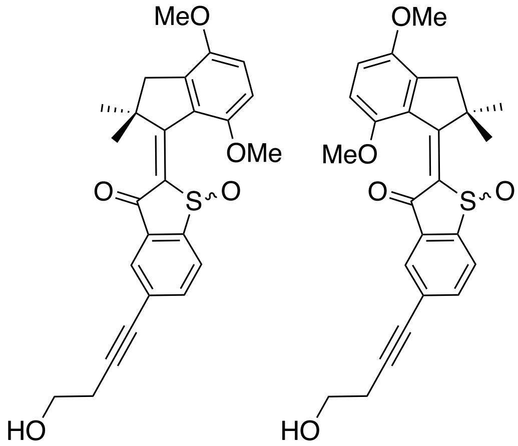

Compound 4. Compound **25** (50 mg, 0.09 mmol) was dissolved in 1 mL THF. Tetrabutylammonium fluoride (233 μL, 1.0 M in THF) was added, and the reaction was stirred for 30 minutes at room temperature. The reaction was then quenched with the addition of water and extracted with ethyl acetate. The product was purified with column chromatography (5-10% acetone in CH_2Cl_2) to afford **4** as a golden yellow solid (39 mg, 98%). ^{1}H NMR (500 MHz, $CDCl_3$) δ 8.00 (set of overlapping d, unable to measure J, 0.57H), 7.96 (d, J = 0.7 Hz, 0.62H), 7.93 (d, J = 7.8 Hz, 0.68H), 7.80 (dd, J_1 = 7.8 Hz, J_2 = 1.1 Hz, 0.33H), 7.78 (dd, J_1 = 7.8 Hz, J_2 = 1.4 Hz, 0.66H), 7.02 (d, J = 9.0 Hz, 0.69 H), 6.96 (d, J = 9.0 Hz, 0.32H), 6.82 (d, J = 8.8 Hz, 0.69 H), 6.72 (d, J = 8.8 Hz, 0.32H), 4.05 (s, 2.08H), 3.86-3.81 (m, 5.07H, set of overlapping signals), 3.75 (s,

0.91H), 3.08 (d, J = 15.3 Hz, 0.35H), 3.00 (d, J = 16.9 Hz, 0.74H), 2.91 (d, J = 14.7 Hz; overlaps with d, J = 16.8 Hz; total 1.03H), 2.75-2.72 (m, 2.03H), 1.95 (s, 1.19H), 1.57 (s, 2.12H), 1.53 (s, 2.15H), 1.43 (s, 0.90H) ppm. ^{13}C NMR (126 MHz, $CDCl_3$) δ 184.07, 182.07, 171.47, 170.52, 153.16, 151.35, 149.97, 149.59, 148.95, 147.50, 142.31, 139.01, 137.87, 137.65, 137.53, 135.77, 135.70, 128.72, 128.03, 127.77, 127.45, 127.39, 126.75, 126.26, 126.20, 116.64, 116.04, 109.64, 109.35, 90.89, 90.56, 80.74, 80.73, 60.91, 60.88, 56.07, 55.96, 55.26, 54.97, 52.63, 52.23, 47.18, 46.42, 28.58, 28.42, 26.05, 25.23, 23.83, 23.82. HRMS (ESI) *m/z* calculated for $[M+H^+]$ $C_{25}H_{24}O_5S$ 437.1417; found 437.1399.

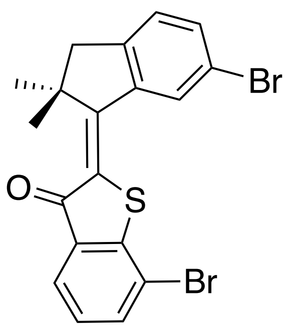

Compound 6. Under argon atmosphere, compound **13** (268 mg, 1.17 mmol) was dissolved in freshly distilled anhydrous DCM (7 mL). The solution was cooled to -78 °C, and BCl_3 (1.2 mL, 1.0 M in DCM) was added. The solution was quickly cannulated into a second solution, precooled to 0 °C, of compound **26** (206 mg, 0.86 mmol) in 2 mL anhydrous DCM. The reaction was stirred at 0 °C for 1 h 45 min. At this point, the reaction was quenched with the addition of saturated aqueous $NaHCO_3$, and the mixture was extracted with DCM three times. After purification with column chromatography (1% ether in hexanes), compound **6** was isolated as a orange solid (220 mg, mixture of isomers, 57%).^1H NMR (500 MHz, $CDCl_3$) 9.23 (dd, J_1 = 1.8 Hz, J_2 = 0.4 Hz, 0.48H), 8.32 (dd, J_1 = 1.7 Hz, J_2 = 0.4 Hz, 0.46H), 7.89 (dd, J_1 = 7.6 Hz, J_2 = 1.1 Hz, 0.43H), 7.83 (dd, J_1 = 7.6 Hz, J_2 = 1.0 Hz, 0.44H), 7.72 (set of two overlapping dd, J_1 = 7.8 Hz, J_2 = 1.1 Hz,

and J_1 = 7.8 Hz, J_2 = 1.14 Hz, 0.90H total), 7.54 (dd, J_1=8.1 Hz, J_2 = 1.7 Hz, 0.45H), 7.51 (dd J_1 = 8.1 Hz, J_2 = 1.9 Hz, 0.44H), 7.22-7.16 (m, set of overlapping signals, 1.81 H), 3.04 (s, 0.99H), 2.97 (s, 0.92H), 1.63 (s, 2.63H), 1.57 (s, 2.99) ppm. ^{13}C NMR (126 MHz, $CDCl_3$) δ 187.32, 187.29, 163.48, 162.86, 148.64, 147.04, 145.99, 145.40, 141.82, 139.72, 137.07, 136.99, 134.57, 134.27, 133.97, 133.77, 131.52, 130.79, 127.93, 126.83, 126.71, 126.56, 126.29, 126.12, 125.59, 125.23, 120.91, 120.07, 117.32, 117.10, 50.86, 49.32, 49.17, 48.85, 26.72, 25.29. HRMS (ESI) *m/z* calculated for [M+H$^+$] $C_{19}H_{14}Br_2OS$ 448.9205; found, 448.9207.

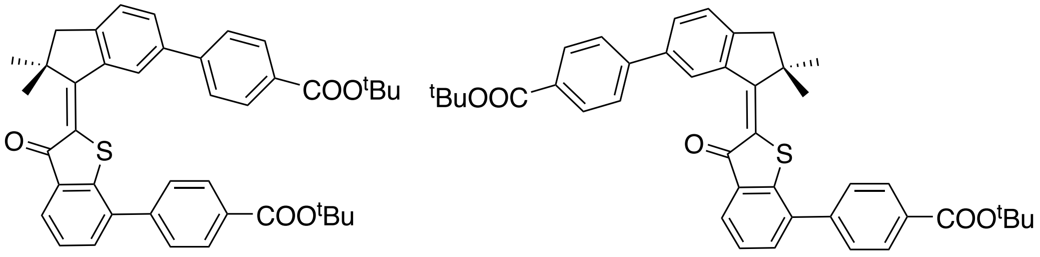

Compound 28. An oven-dried screw-cap tube, charged with compound **6** (60 mg, 0.13 mmol), *tert*-butyl 4-(4,4,5,5-tetramethyl-1,3,2-dioxaborolan-2-yl)benzoate (145 mg, 0.48 mmol), and K_3PO_4 (171 mg, 0.81 mmol), was purged with Ar three times. A mixture of EtOH (360 µL) and water (120 µL) was added, and the solution was bubbled with Ar for 30 min. sSPhos Pd G2 (9 mg, 0.10 mmol) was then added, and the solution was stirred at 100 °C overnight. The reaction was then quenched with a saturated solution of NH_4Cl and extracted with EtOAc three times. The solvent was removed under vacuum, and the crude material was purified with column chromatography (5-10% EtOAc in hexanes, then additional column 50% DCM in hexanes) to afford **28** as an orange solid (74 mg, mixture of isomers, 86%). ^{1}H NMR (500 MHz, $CDCl_3$) 9.38 (d, J = 1.78 Hz, 0.40H), 8.31 (d, J = 1.43 Hz, 0.56H), 8.16-8.06 (m, 3.93H, set of overlapping signals), 7.98 (dd, J_1 = 7.52 Hz, J_2 = 1.19 Hz, 0.42H), 7.92 (dd, J_1 = 7.52 Hz, J_2 = 1.19 Hz, 0.55H), 7.77 (m, 0.84H), 7.72-7.68 (m, set of overlapping signals, 1.92H), 7.67-7.61 (m, set of overlapping

signals, 2.10H), 7.60 (dd, J_1 = 7.5 Hz, J_2 = 1.2 Hz) overlaps with 7.59 (dd, J_1 = 7.5 Hz, J_2 = 1.3 Hz, 0.97H total), 7.42- 7.36 (m, set of overlapping signals, 1.93H), 3.14 (s, 1.08H), 3.04 (s, 0.89H), 1.70 (s, 3.49H), 1.64, 1.64, 1.63, 1.63 (s, s, s, s, overlapping signals, 18.03H), 1.53 (s, 2.33 H) ppm. ^{13}C NMR (126 MHz, $CDCl_3$) δ 187.75, 187.66, 166.54, 165.48, 165.40, 165.30, 163.97, 163.34, 149.71, 148.08, 145.21, 144.68, 142.98, 142.54, 142.36, 142.33, 140.75, 139.49, 138.68, 138.36, 135.98, 135.84, 134.59, 134.55, 133.07, 132.89, 131.91, 131.72, 131.12, 130.71, 130.62, 130.05, 129.99, 128.02, 127.93, 127.70, 127.13, 127.04, 126.91, 126.88, 126.46, 126.09, 126.00, 125.87, 125.72, 125.52, 125.14, 81.32, 81.29, 81.03, 80.93, 51.05, 49.13, 36.63, 28.26, 28.22, 26.77, 25.45, 24.69, 23.34 ppm. HRMS (ESI) *m/z* calculated for [M+H$^+$] $C_{41}H_{40}O_5S$ 645.2685; found 645.2669.

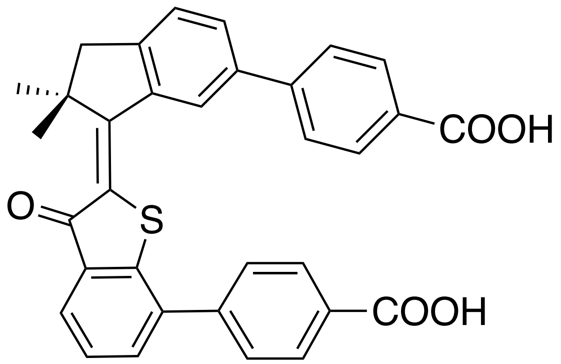

Compound 7. Compound **28** (280 mg, 0.43 mmol) was added to 5 mL CH_2Cl_2 and 40 mL formic acid. After stirring the mixture for 1.5 h at room temperature, the formic acid and CH_2Cl_2 were removed under vacuum. Water was added to the residue, and the aqueous layer was extracted with EtOAc. The combined organic fractions were dried over Na_2SO_4 and concentrated under vacuum. The crude product was rinsed with Et_2O, and no further purification was needed to afford **7** as a yellow solid (*Z* isomer only, 199.8 mg, 86%). ^{1}H NMR (500 MHz, DMSO-d6) 8.30 (d, *J* = 1.3 Hz, 0.97H), 8.11 (m, 1.99H), 8.02 (m, 1.99H), 7.89-7.82 (m, 5.11H), 7.75 (app d, *J* = 8.56 Hz, 2.04H),

7.56-7.53 (m, 2.06H), 3.14 (s, 2.27H), 1.61 (s, 6.00H) ppm. ^{13}C NMR (126 MHz, DMF) δ 187.57, 167.48, 167.34, 163.99, 150.54, 144.60, 142.57, 142.47, 140.67, 139.22, 136.19, 135.38, 132.77, 131.52, 131.23, 130.72, 130.56, 130.41, 128.68, 127.18, 126.96, 126.68, 126.54, 126.23, 125.77, 50.76, 48.97, 25.15 ppm. HRMS (ESI) *m/z* calculated for [M+H$^+$] $C_{33}H_{24}O_5S$ 533.1417; found 533.1428.

C. NMR spectra of synthetic intermediates

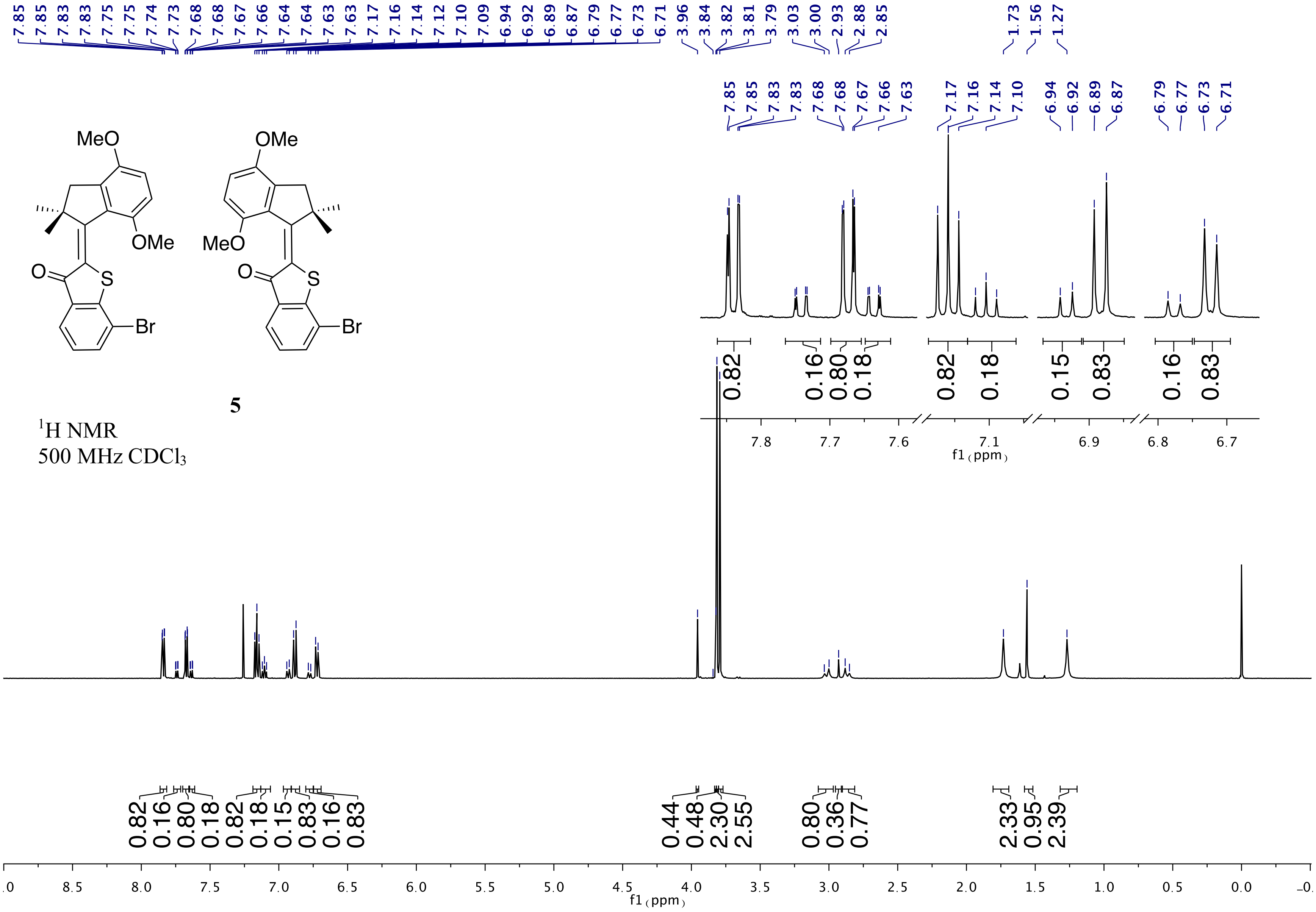

MeO
OMe
OMe
MeO
O
S
Br
5
^{1}H NMR
500 MHz CDCl3
f1 (ppm)

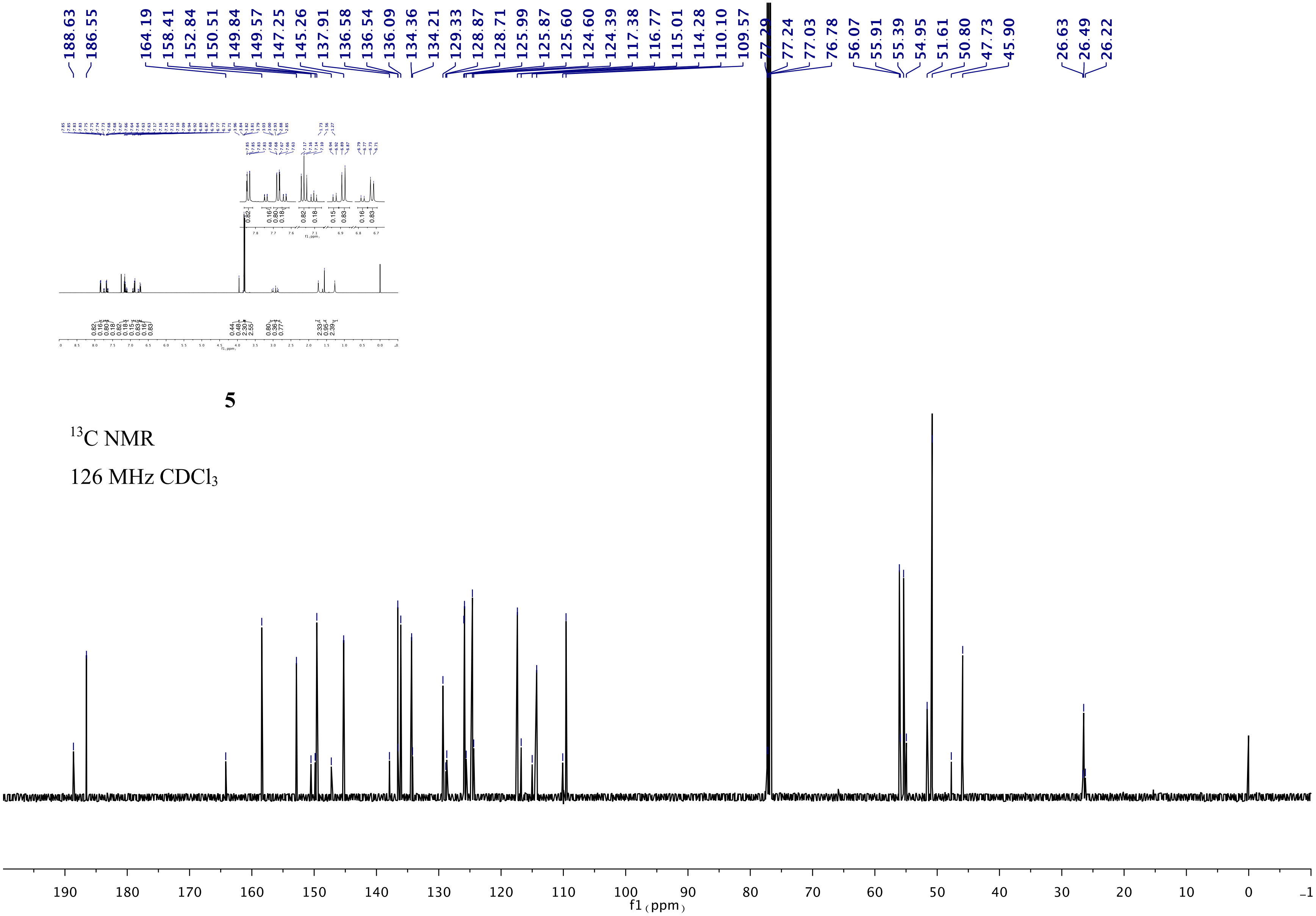

188.63
186.55
164.19
158.41
152.84
150.51
149.84
149.57
147.25
145.26
137.91
136.58
136.54
136.09
134.36
134.21
129.33
128.87
128.71
125.99
125.87
125.60
124.60
124.39
117.38
116.77
115.01
114.28
110.10
109.57
77.24
77.03
76.78
56.07
55.91
55.39
54.95
51.61
50.80
47.73
45.90
26.63
26.49
26.22
5
^{13}C NMR
126 MHz $CDCl_3$
f1 (ppm)

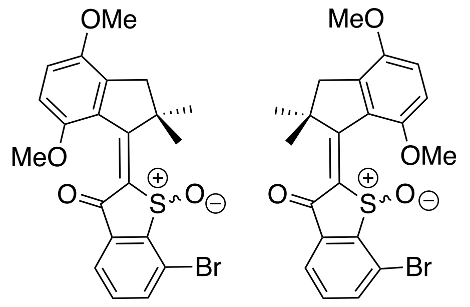

1

^{1}H NMR

500 MHz $CDCl_3$

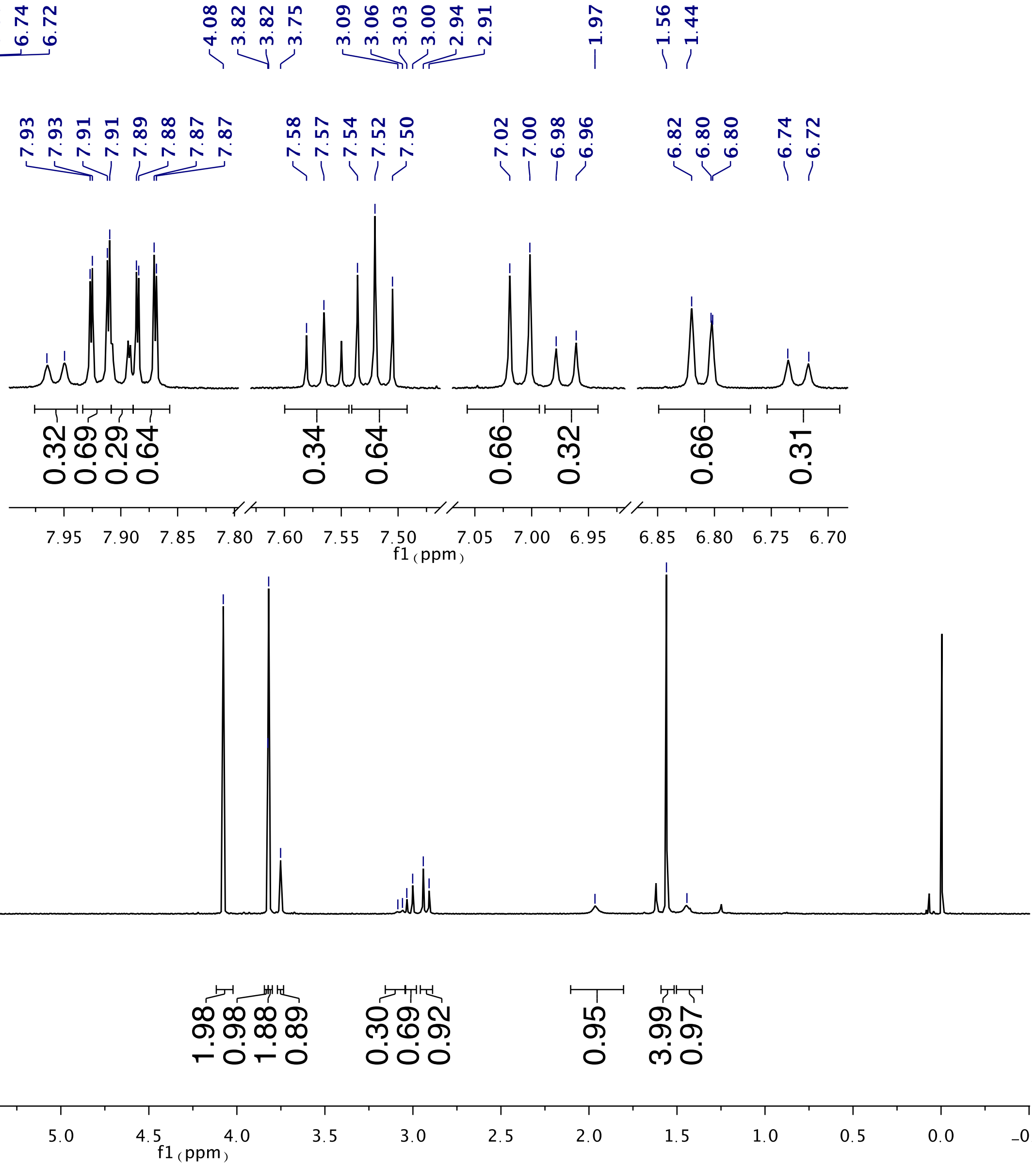

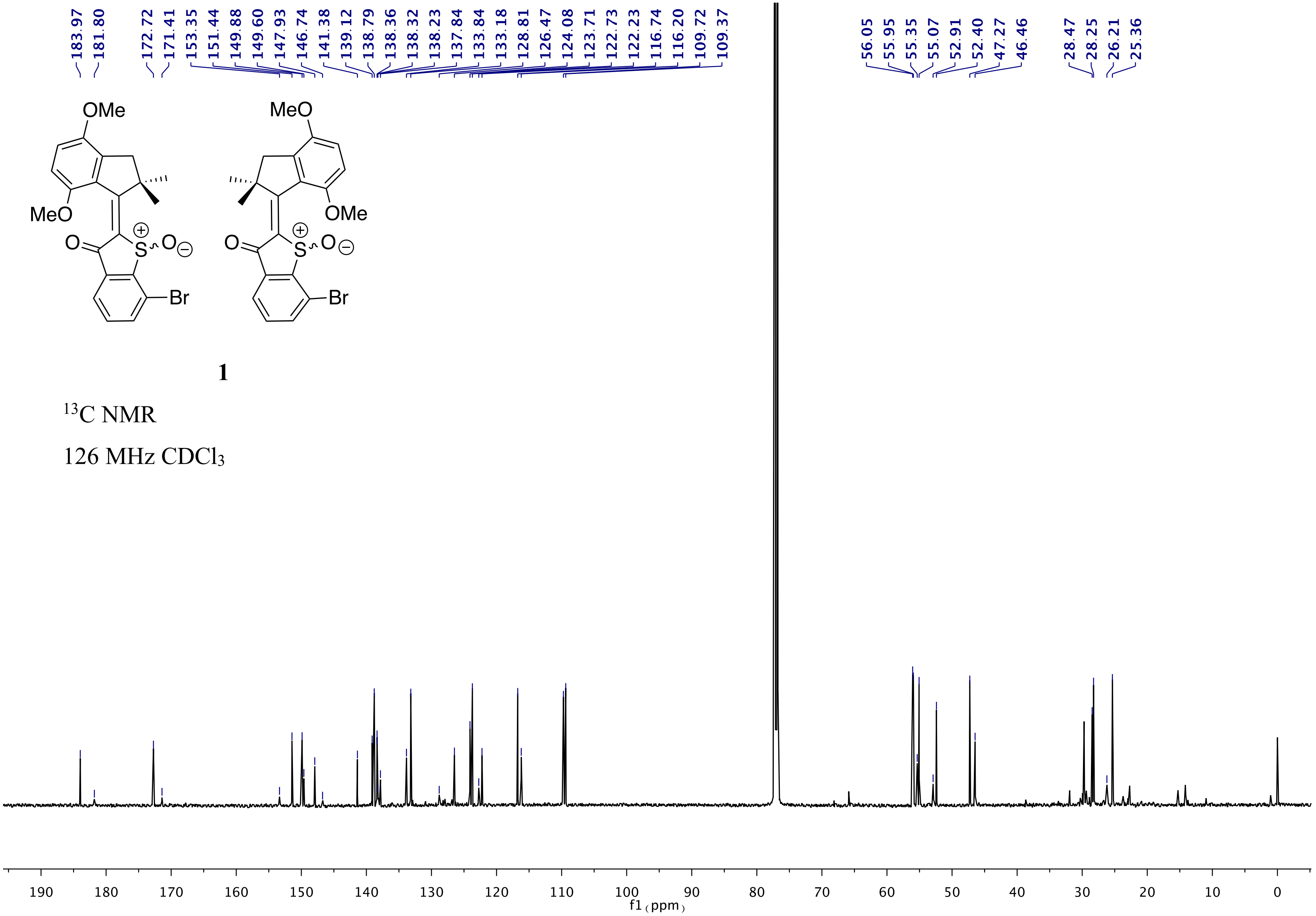
183.97
181.80
172.72
171.41
153.35
151.44
149.88
149.60
147.93
146.74
141.38
139.12
138.79
138.36
138.32
138.23
137.84
133.84
133.18
128.81
126.47
124.08
123.71
122.73
122.23
116.74
116.20
109.72
109.37
56.05
55.95
55.35
55.07
52.91
52.40
47.27
46.46
28.47
28.25
26.21
25.36
OMe
MeO
MeO
OMe
O
S
O
Br
1
^{13}C NMR
126 MHz $CDCl_3$
190 180 170 160 150 140 130 120 110 100 90 80 70 60 50 40 30 20 10 0
f1 (ppm)

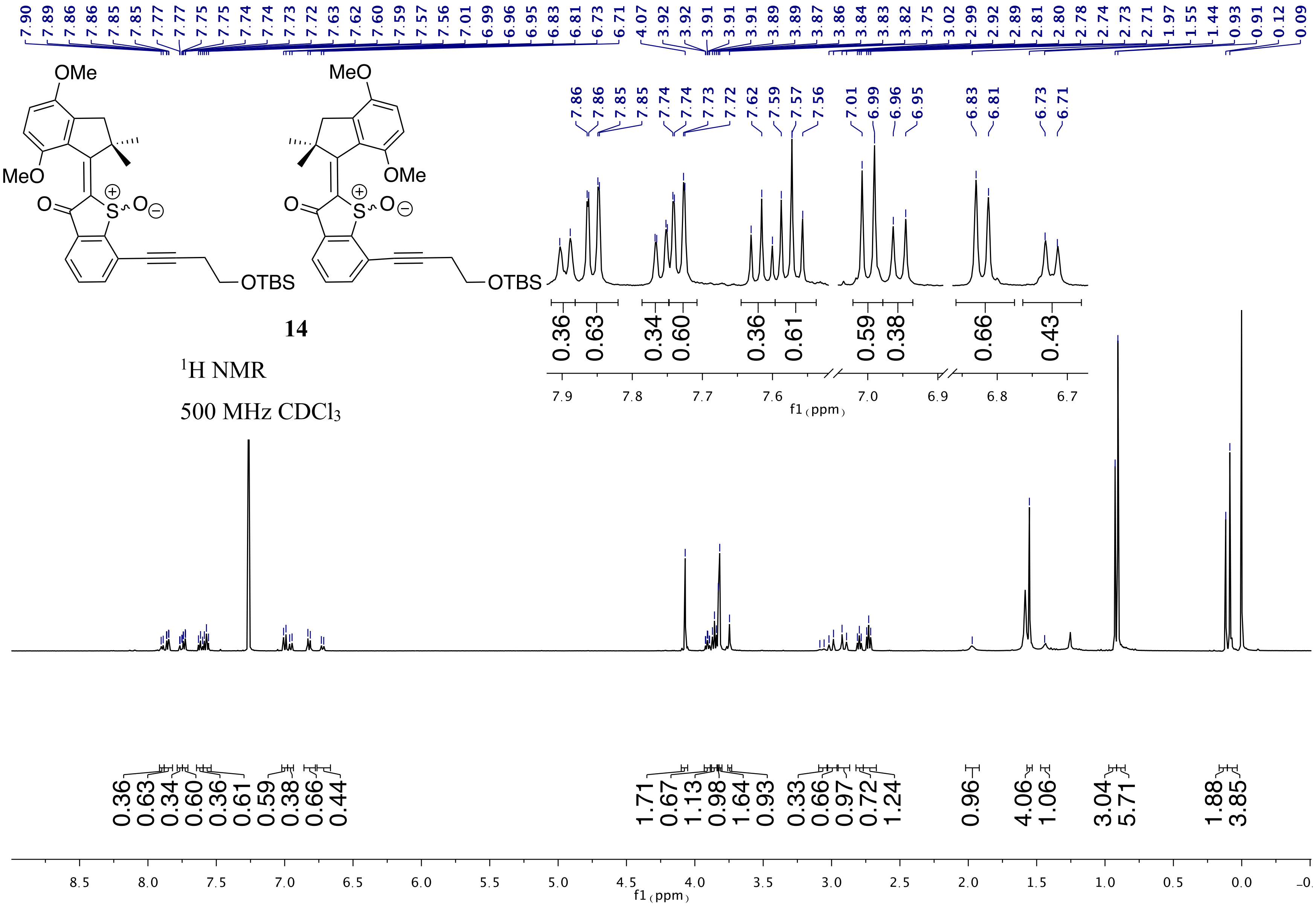
OMe
MeO
OTBS
14
1H NMR
500 MHz CDCl3
f1 (ppm)

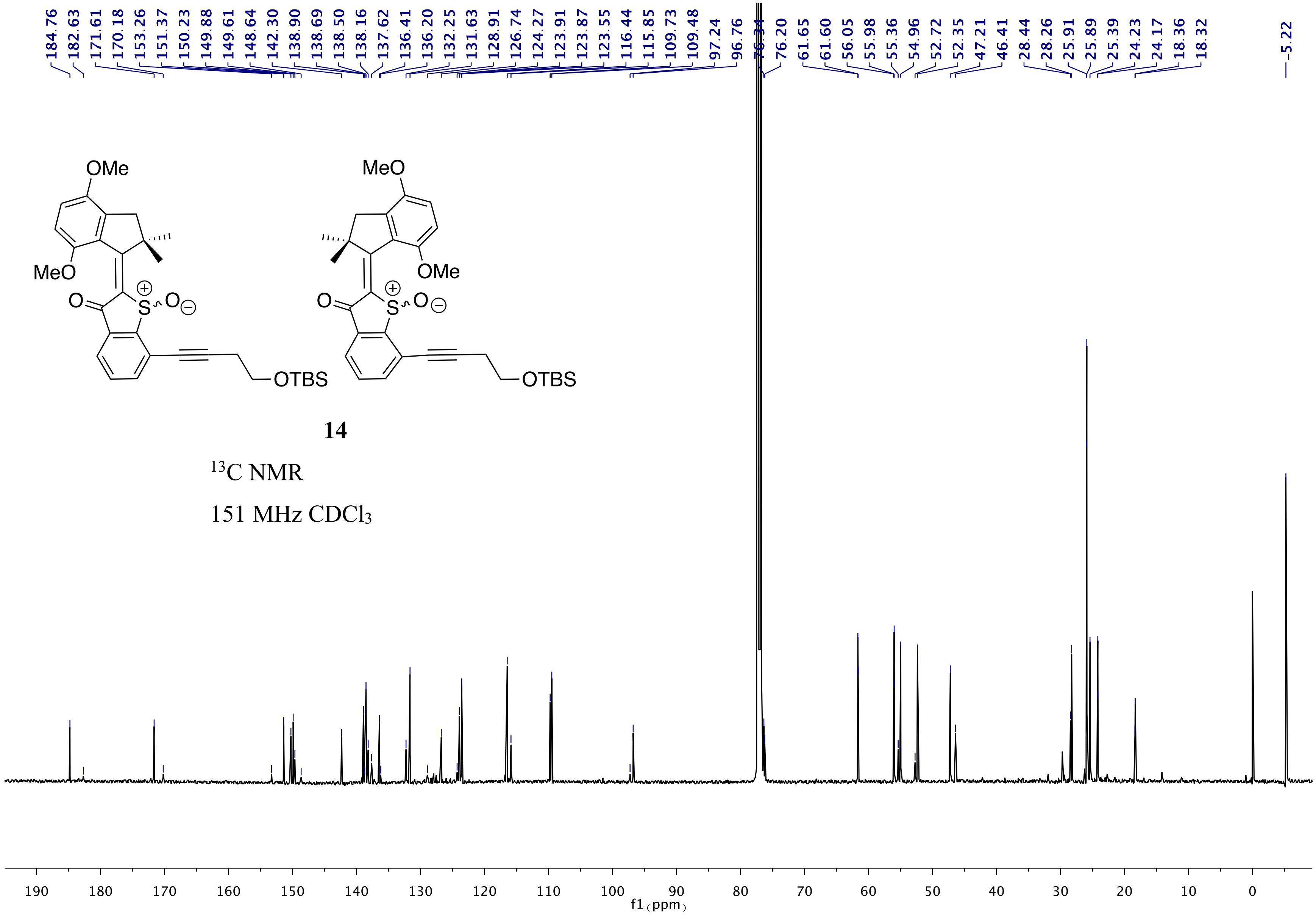

14

^{13}C NMR

151 MHz $CDCl_3$

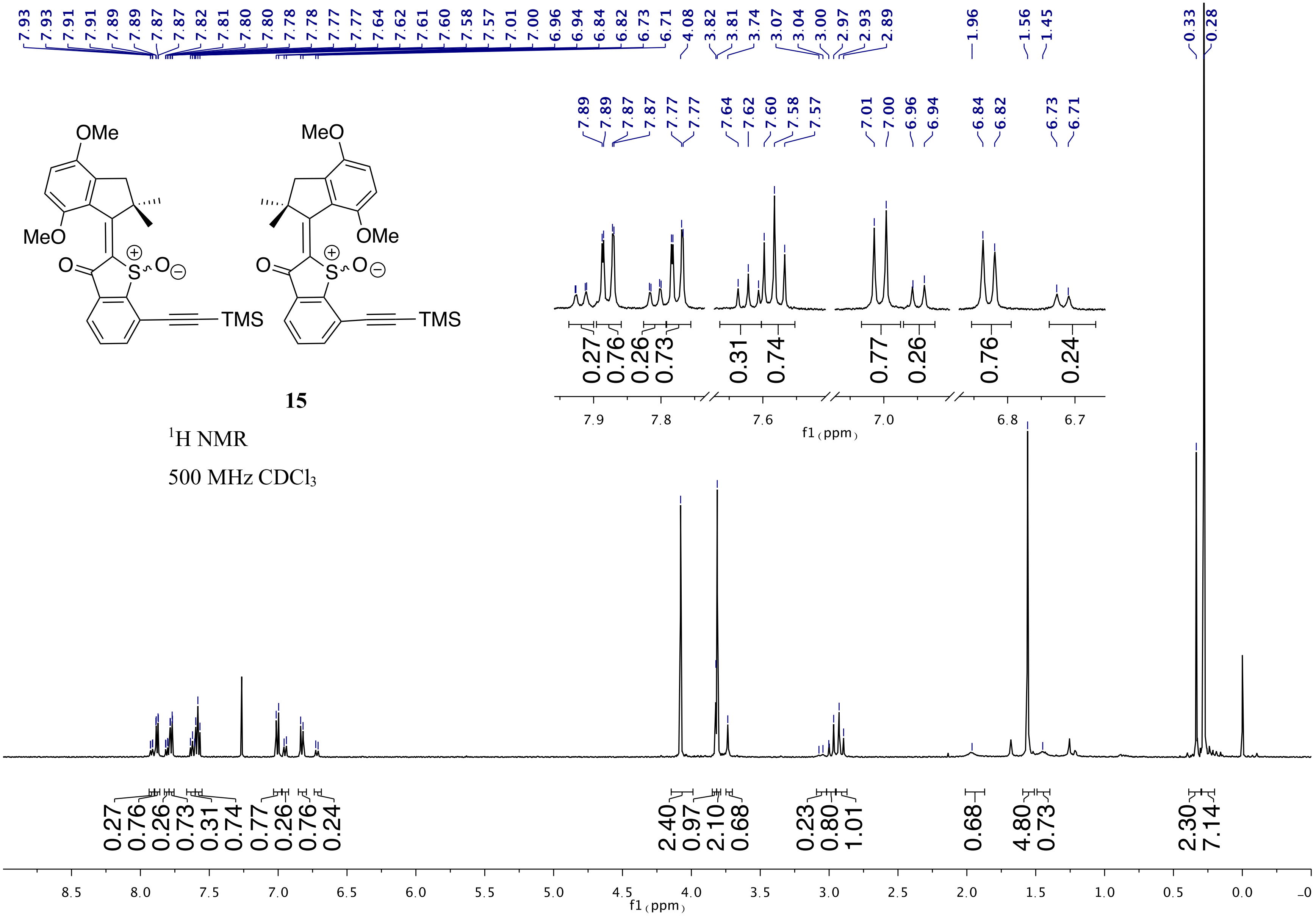
OMe
MeO
O
S
O
TMS
15
^{1}H NMR
500 MHz CDCl3
f1 (ppm)

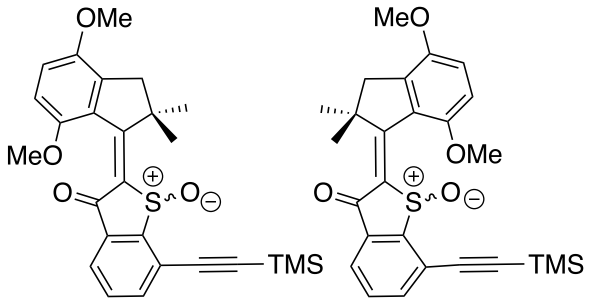

15

^{13}C NMR

151 MHz $CDCl_3$

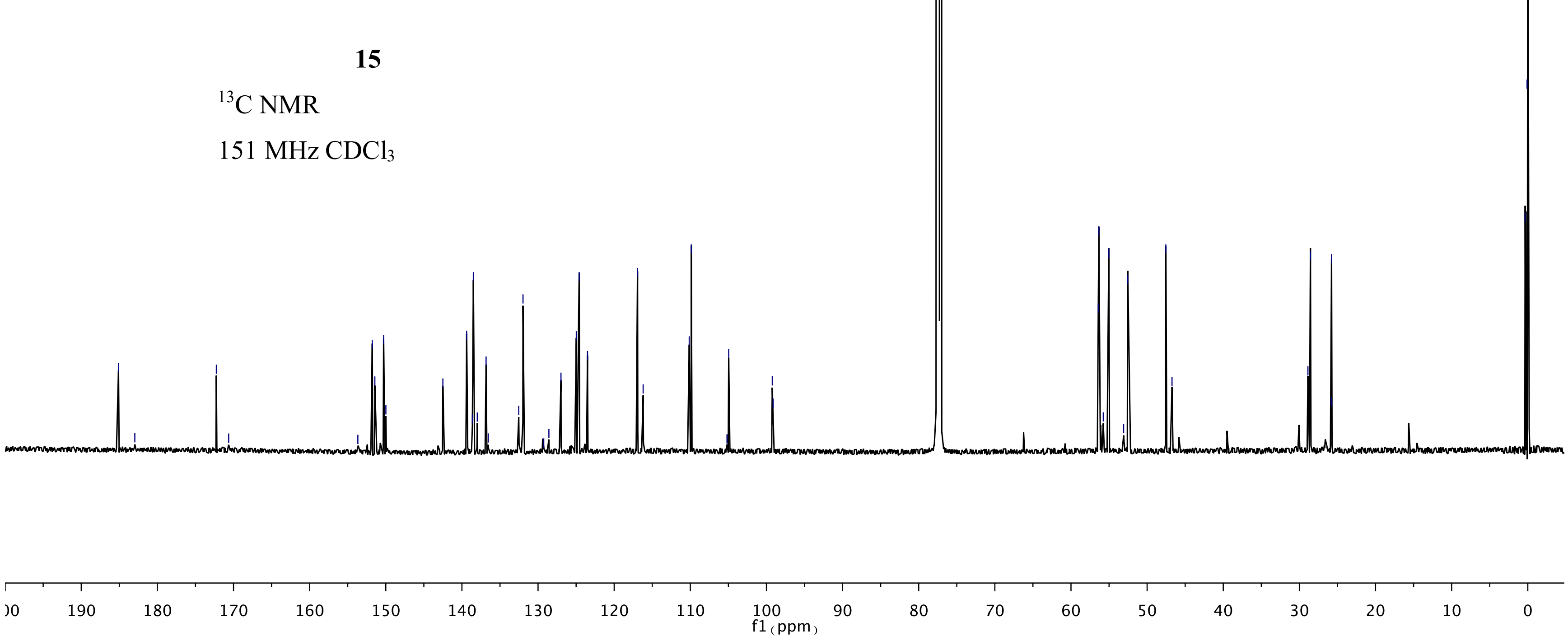

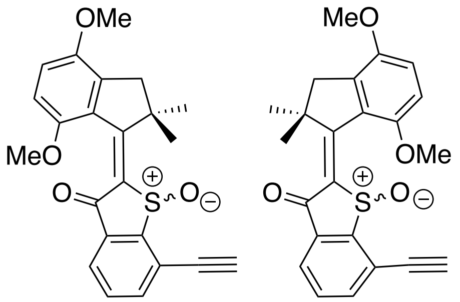

16

^{1}H NMR

500 MHz $CDCl_3$

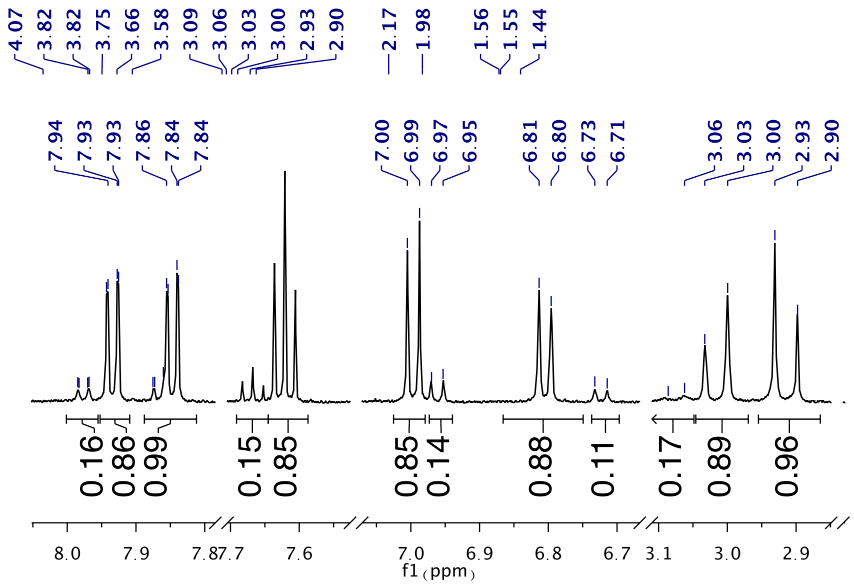

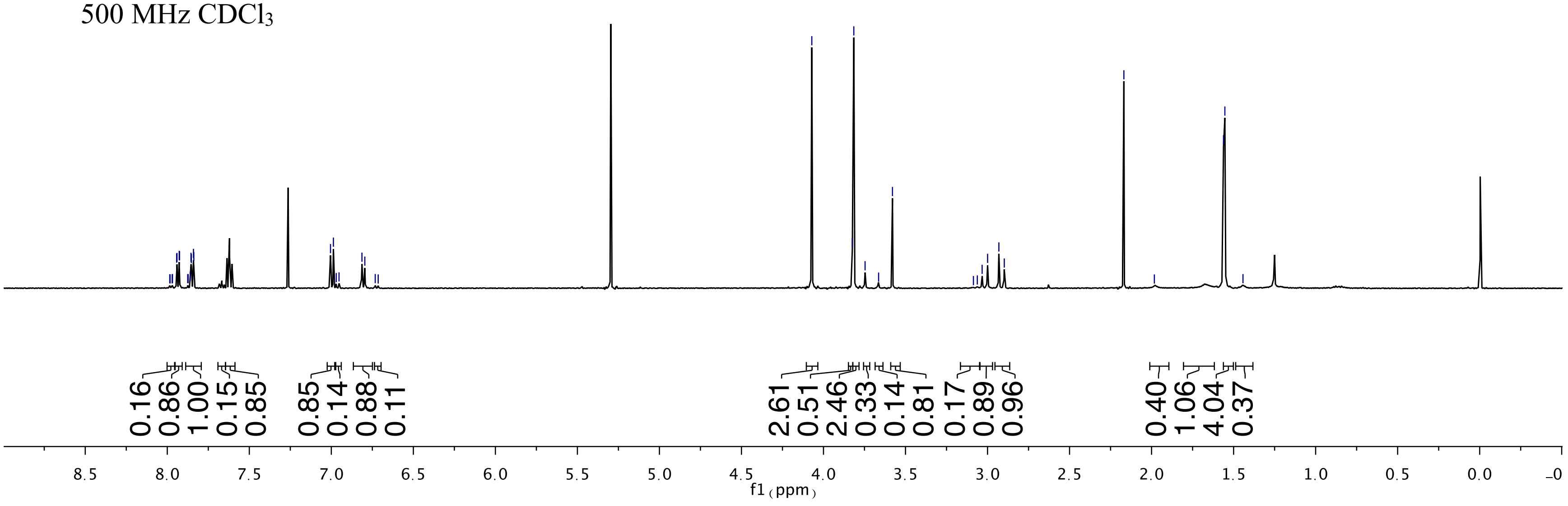

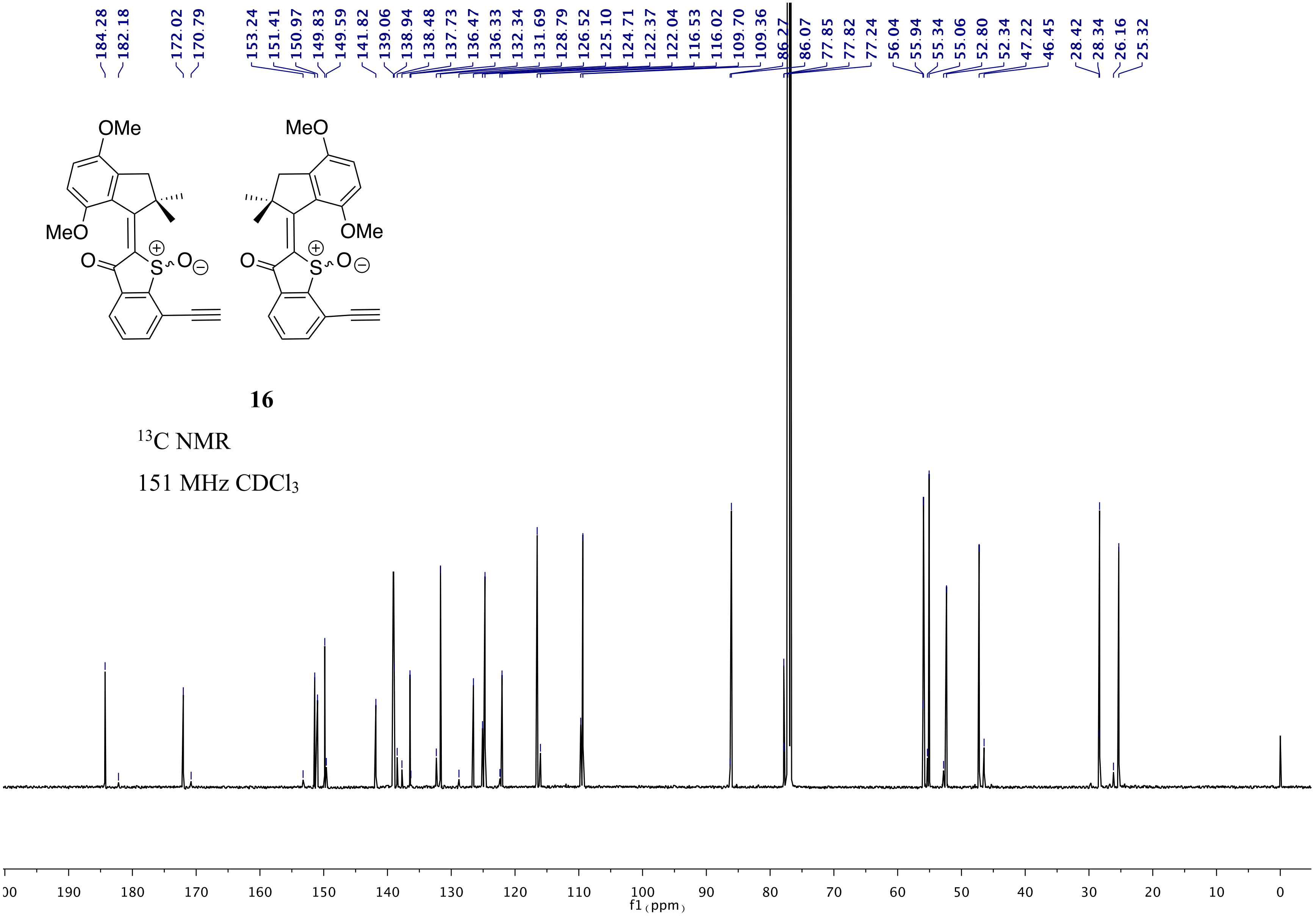

184.28
182.18
172.02
170.79
153.24
151.41
150.97
149.83
149.59
141.82
139.06
138.94
138.48
137.73
136.47
136.33
132.34
131.69
128.79
126.52
125.10
124.71
122.37
122.04
116.53
116.02
109.70
109.36
86.27
86.07
77.85
77.82
77.24
56.04
55.94
55.34
55.06
52.80
52.34
47.22
46.45
28.42
28.34
26.16
25.32
OMe
MeO
O
S
O
MeO
OMe
16
^{13}C NMR
151 MHz $CDCl_3$
f1 (ppm)

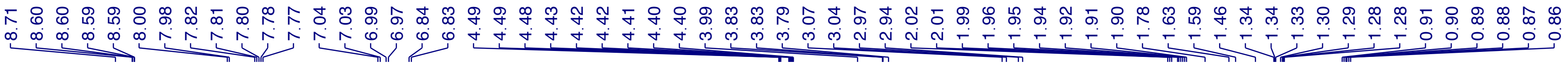

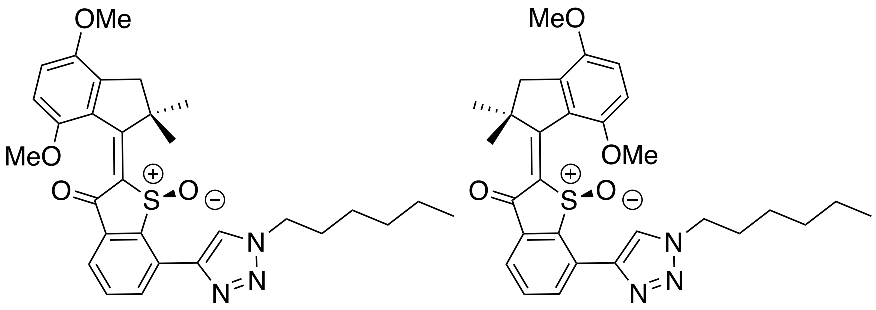

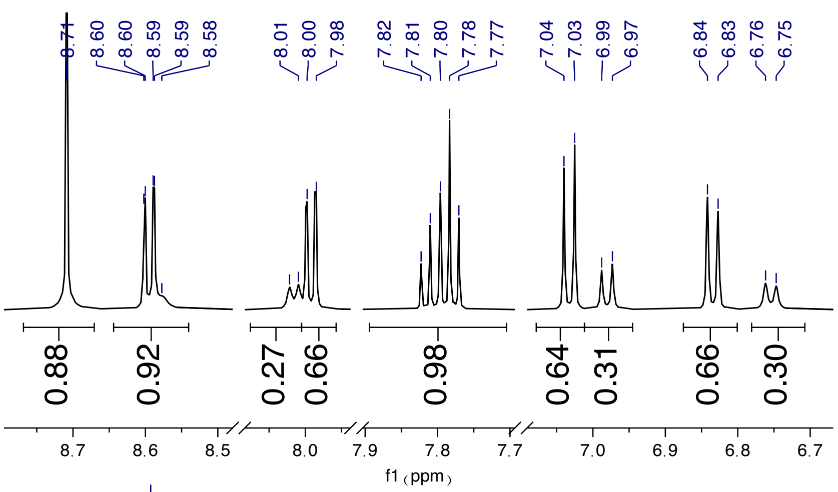

3

^{1}H NMR

600 MHz $CDCl_3$

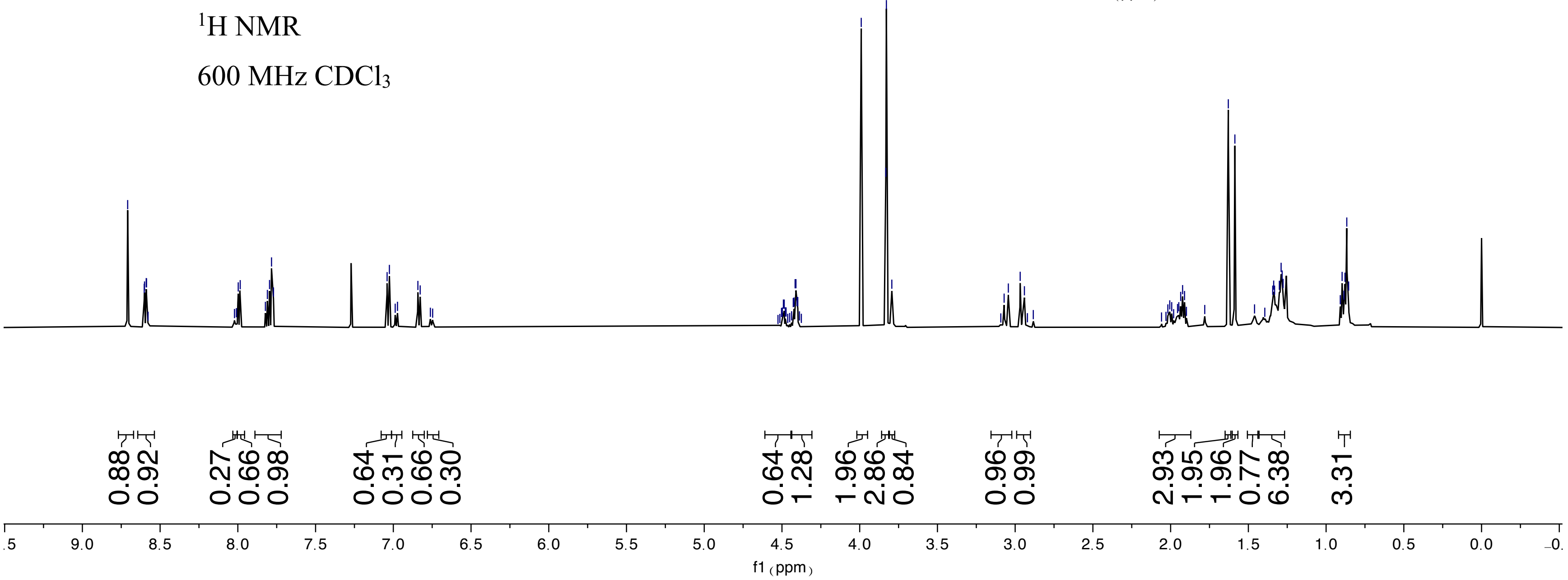

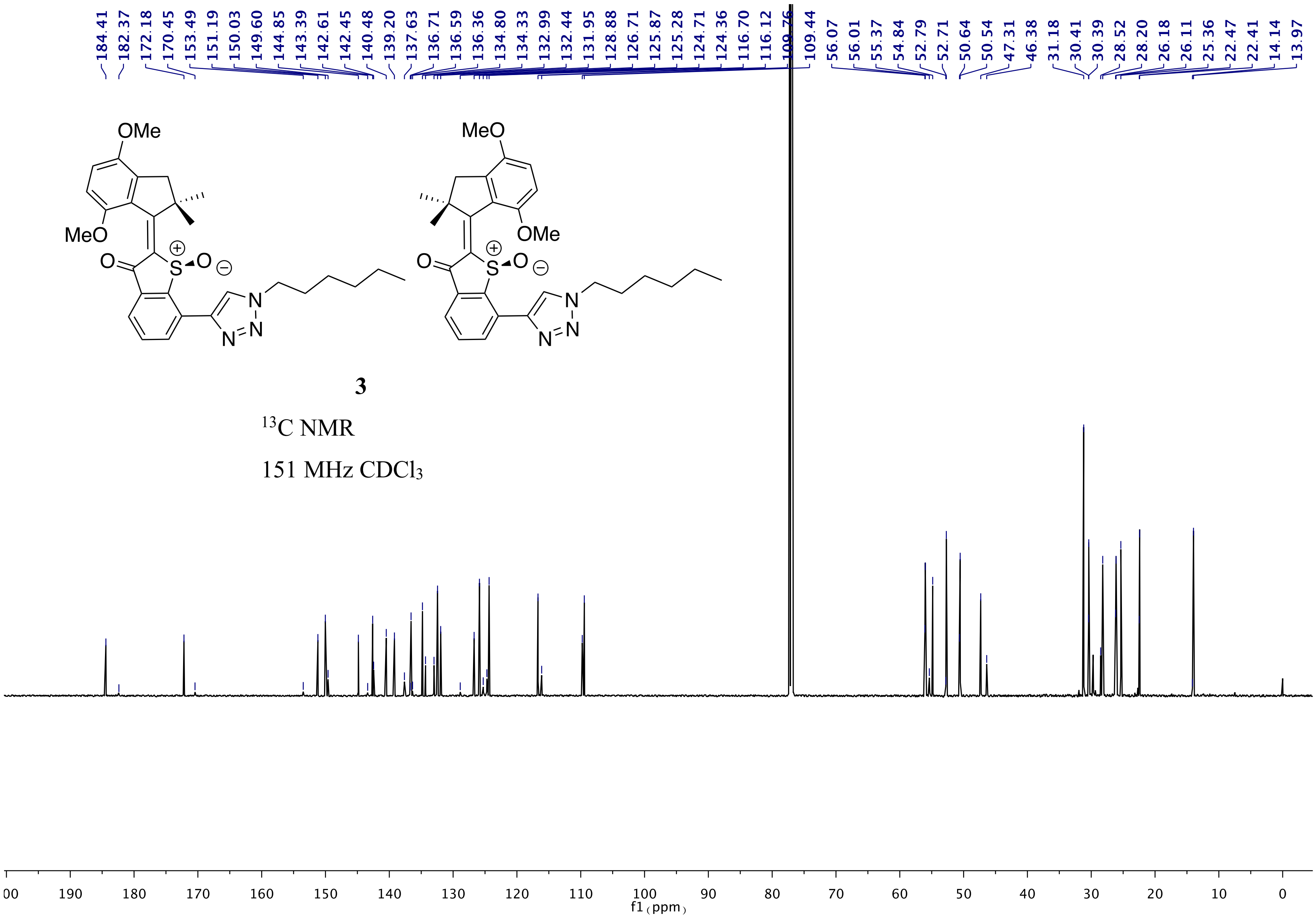
184.41
182.37
172.18
170.45
153.49
151.19
150.03
149.60
144.85
143.39
142.61
142.45
140.48
139.20
137.63
136.71
136.59
136.36
134.80
134.33
132.99
132.44
131.95
128.88
126.71
125.87
125.28
124.71
124.36
116.70
116.12
109.76
109.44
56.07
56.01
55.37
54.84
52.79
52.71
50.64
50.54
47.31
46.38
31.18
30.41
30.39
28.52
28.20
26.18
26.11
25.36
22.47
22.41
14.14
13.97
OMe
MeO
O
S
O
N
N
N
3
^{13}C NMR
151 MHz CDCl$_3$
f1 (ppm)

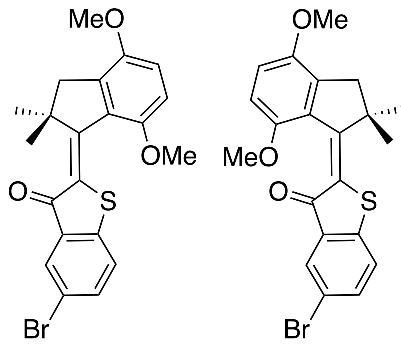

23

^{1}H NMR

500 MHz $CDCl_3$

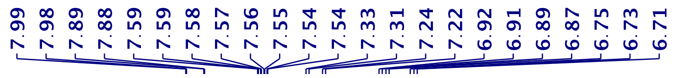

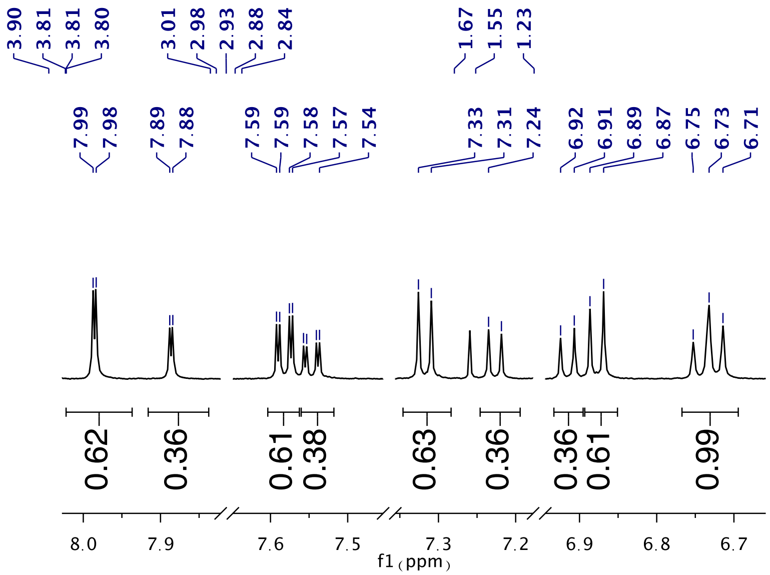

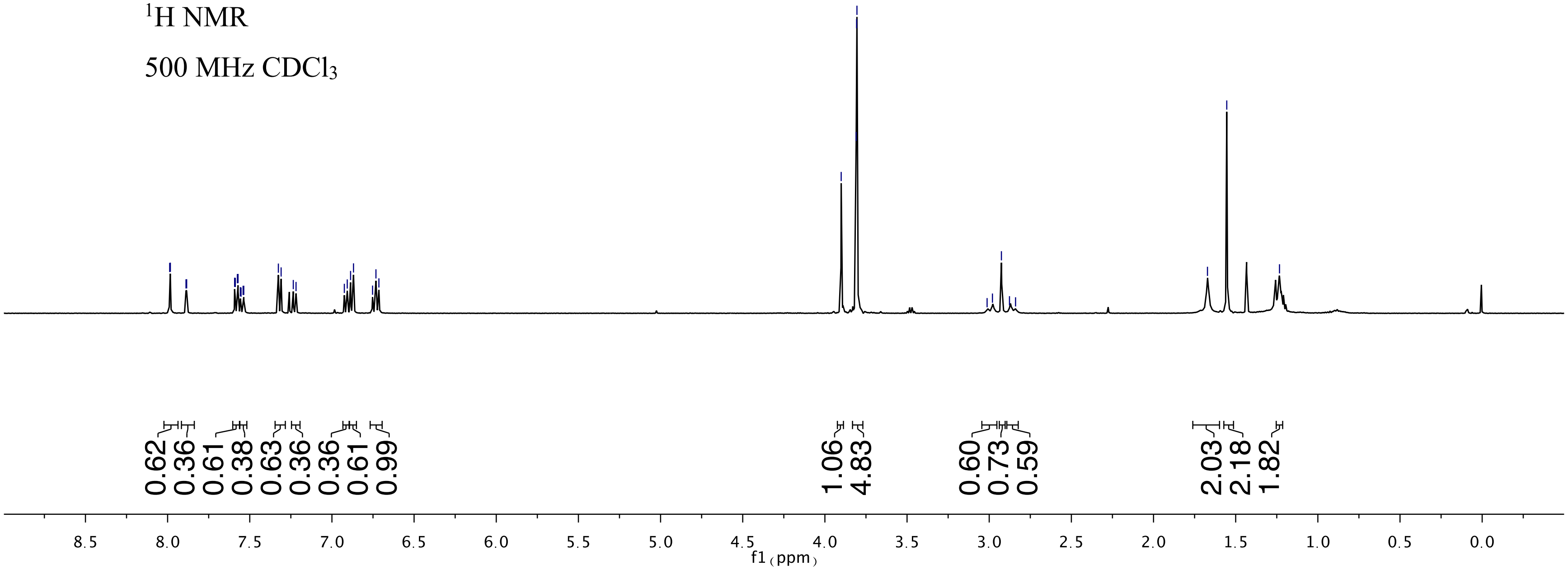

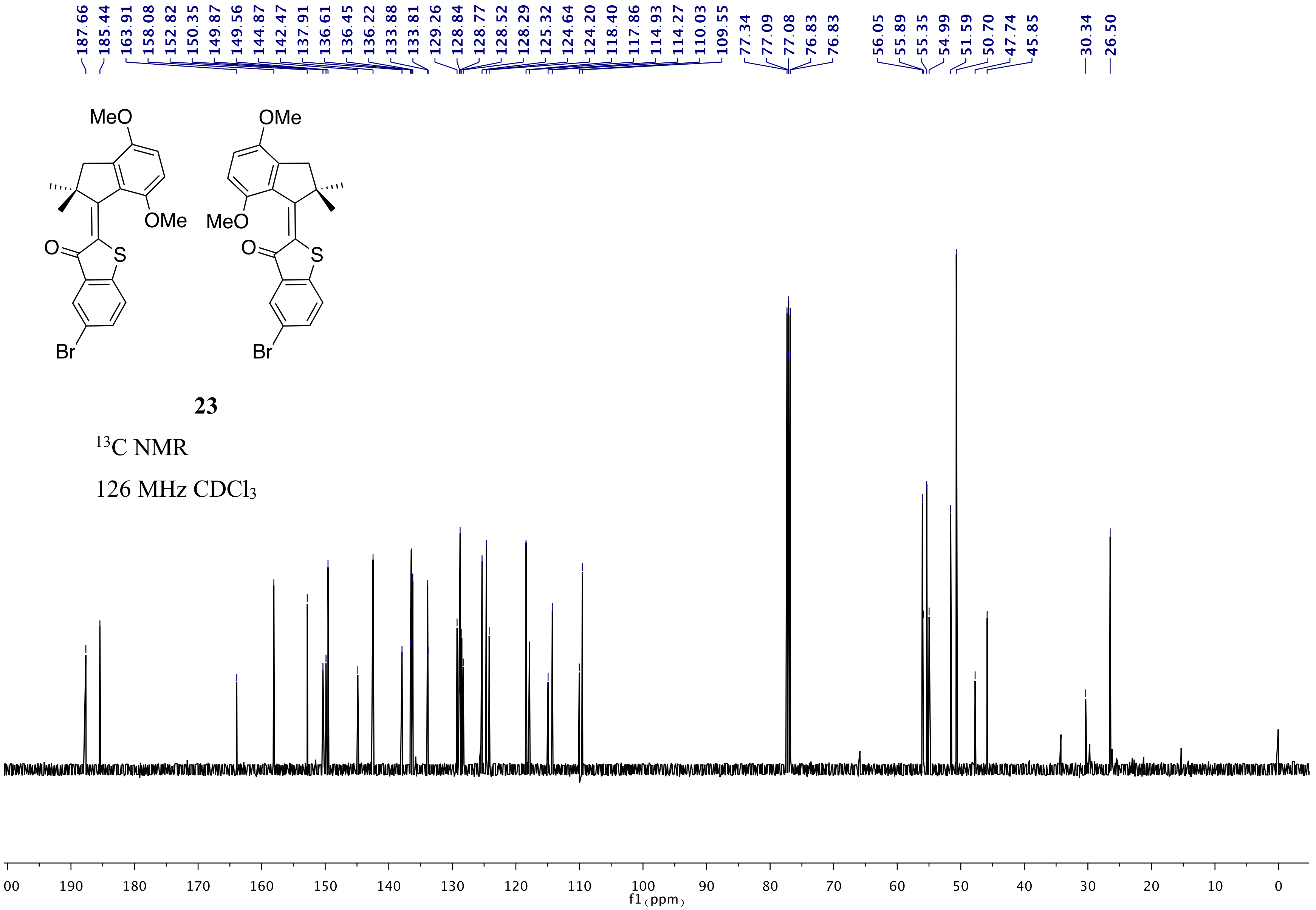

23

^{13}C NMR

126 MHz $CDCl_3$

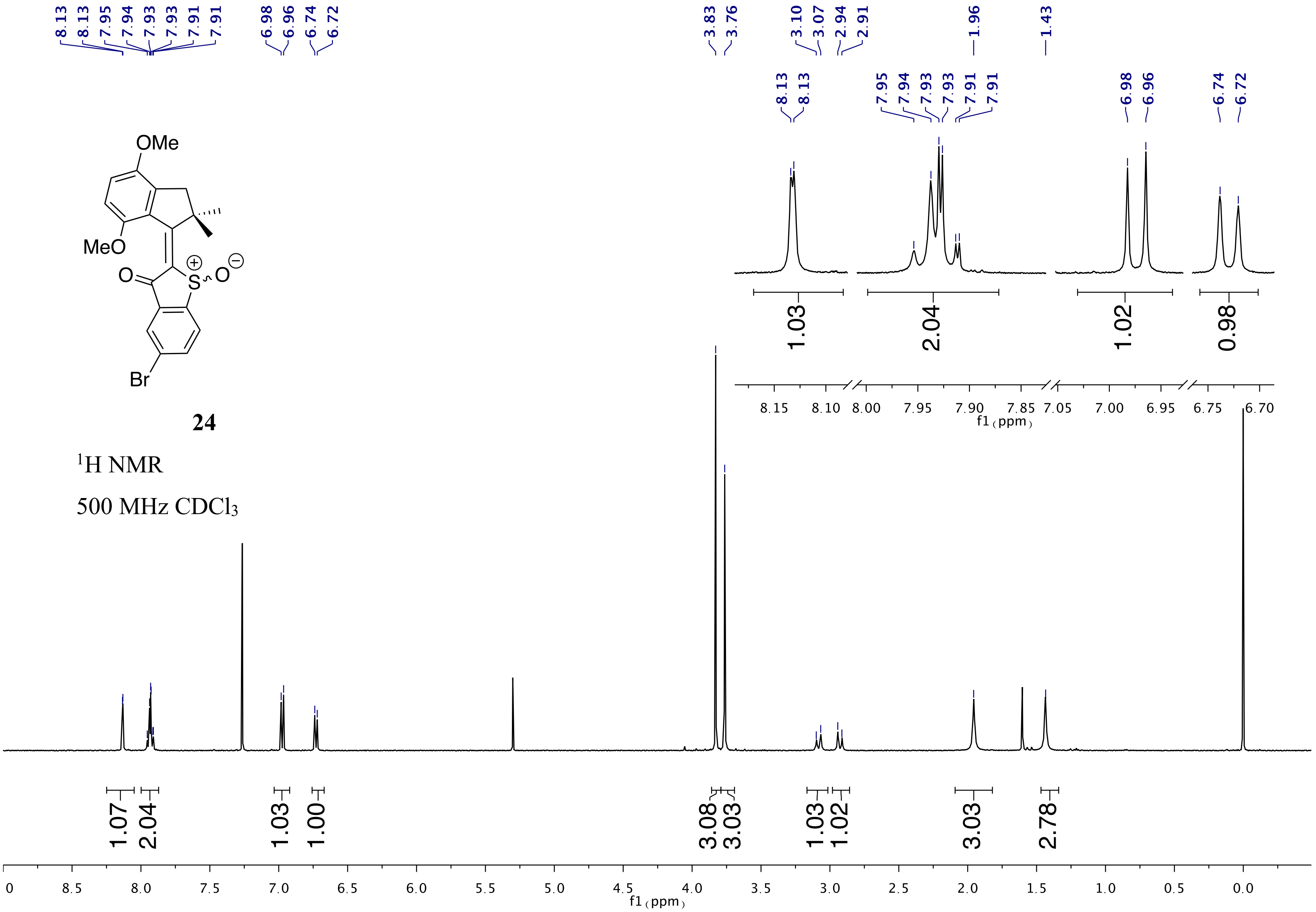
8.13 8.13 7.95 7.94 7.93 7.93 7.91 7.91
6.98 6.96 6.74 6.72
3.83 3.76
3.10 3.07 2.94 2.91
1.96
1.43
OMe
MeO
O
S
O
Br
24
^{1}H NMR
500 MHz $CDCl_3$
1.03 2.04 1.02 0.98
1.07 2.04 1.03 1.00 3.08 3.03 1.03 1.02 3.03 2.78
f1 (ppm)

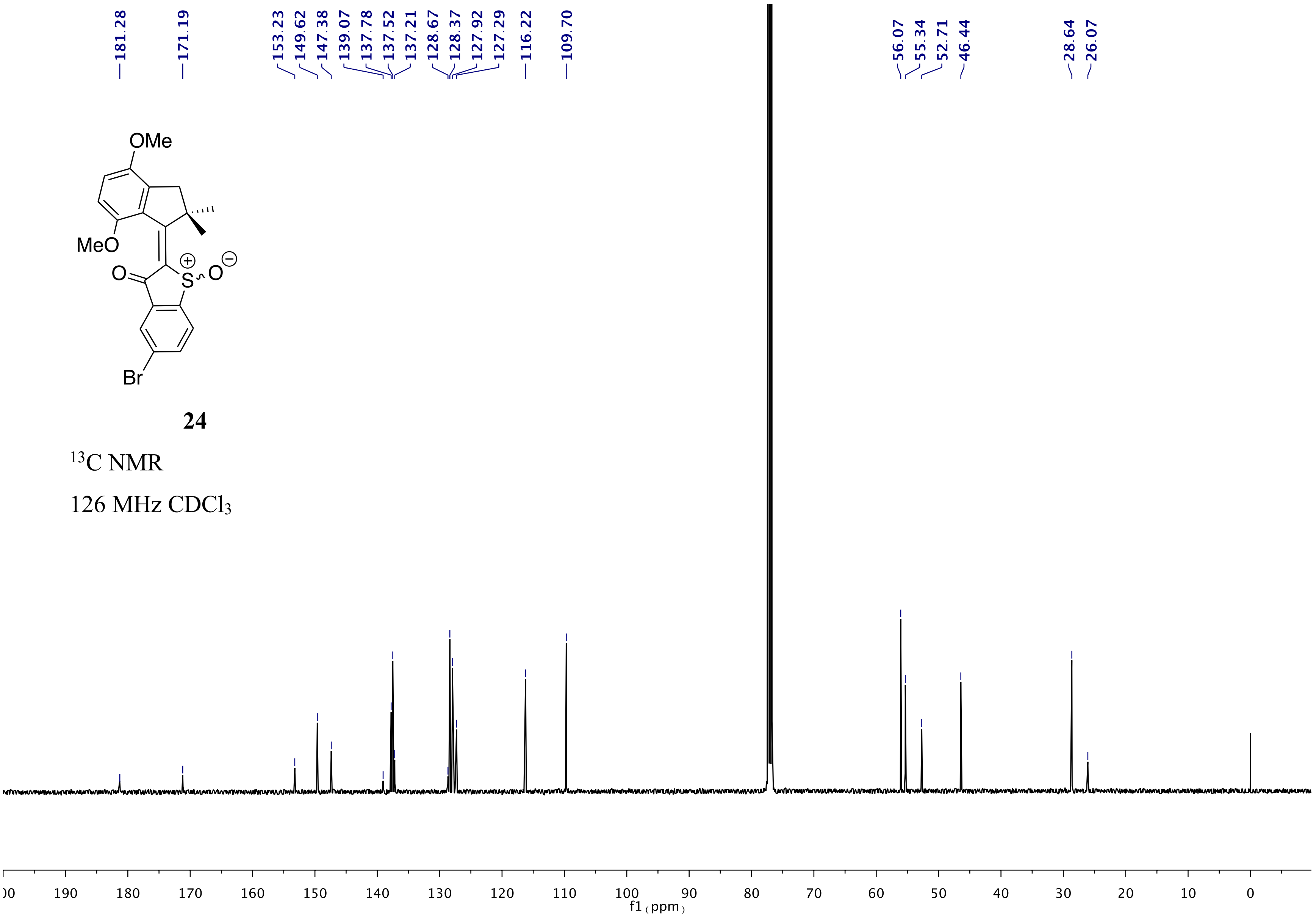

24

^{13}C NMR

126 MHz $CDCl_3$

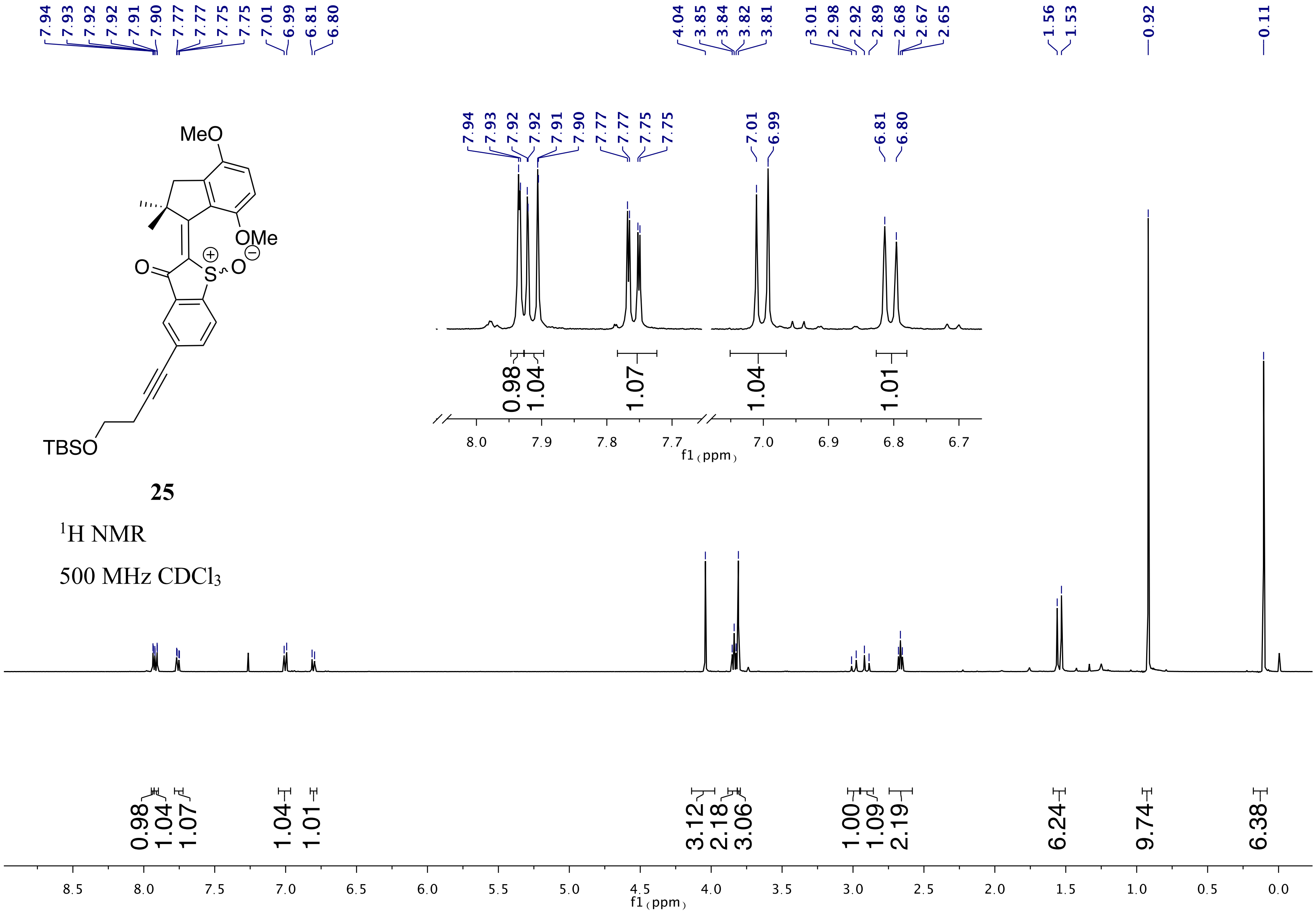

25

^{1}H NMR

500 MHz $CDCl_3$

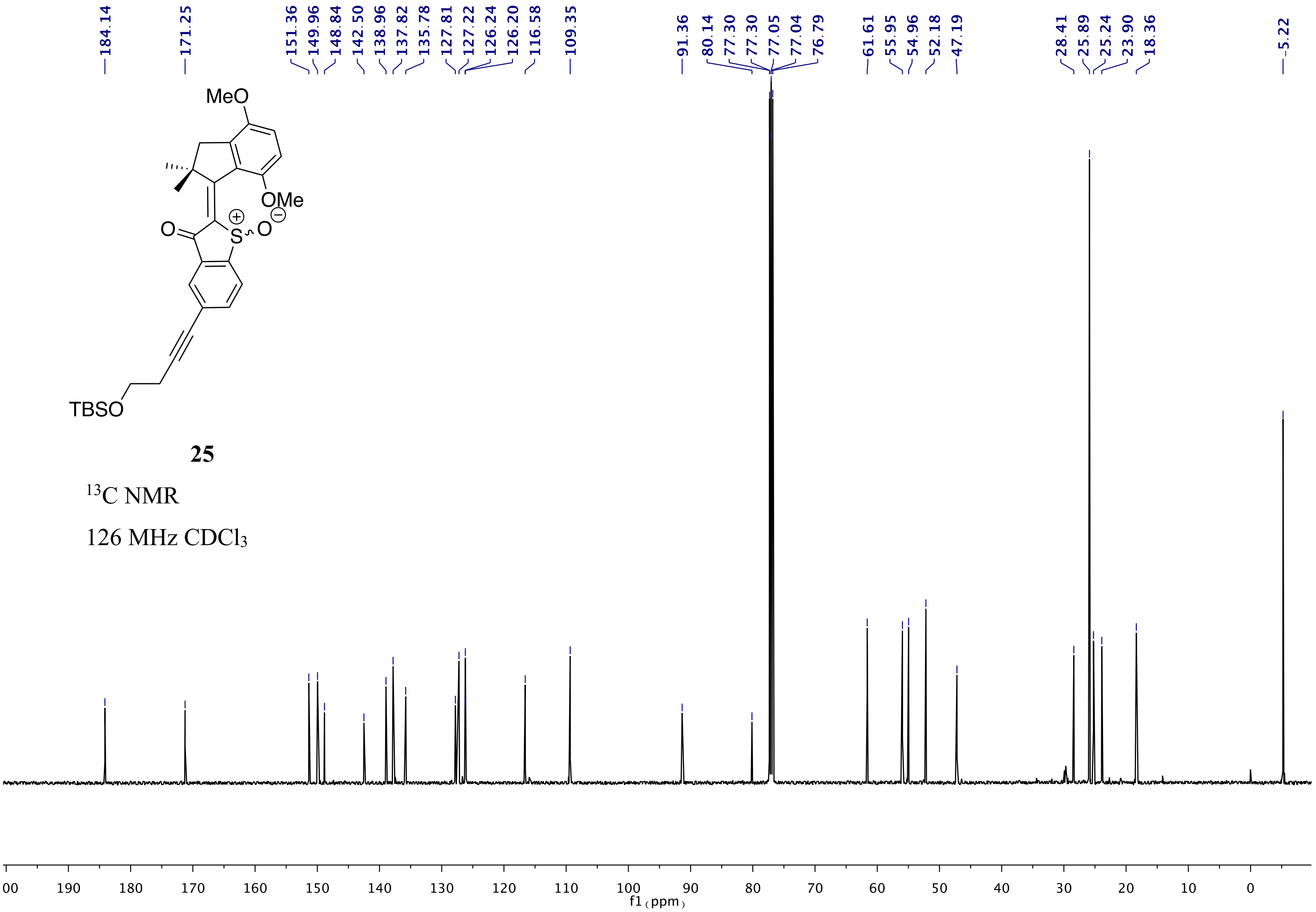

25

^{13}C NMR

126 MHz $CDCl_3$

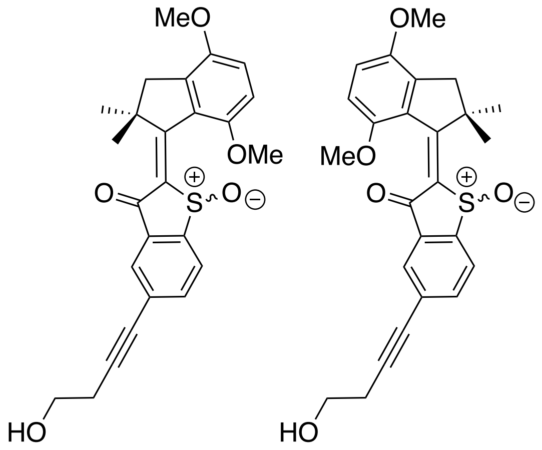

4

^{1}H NMR

600 MHz $CDCl_3$

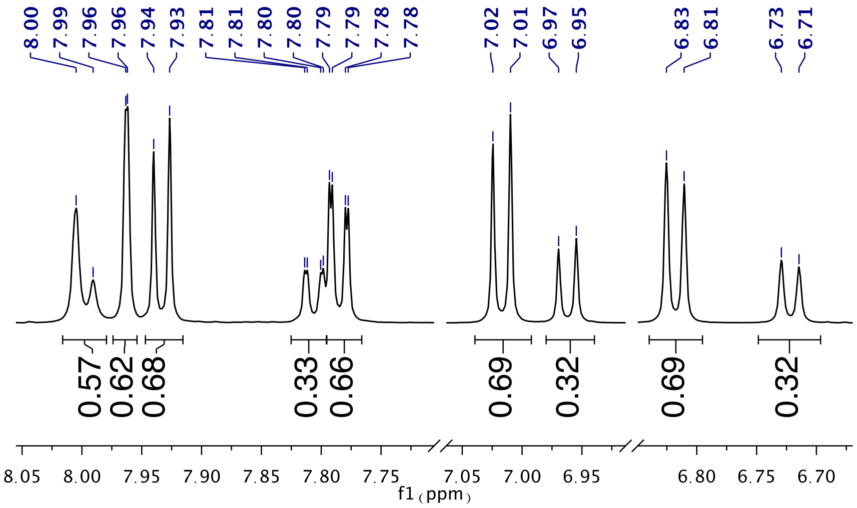

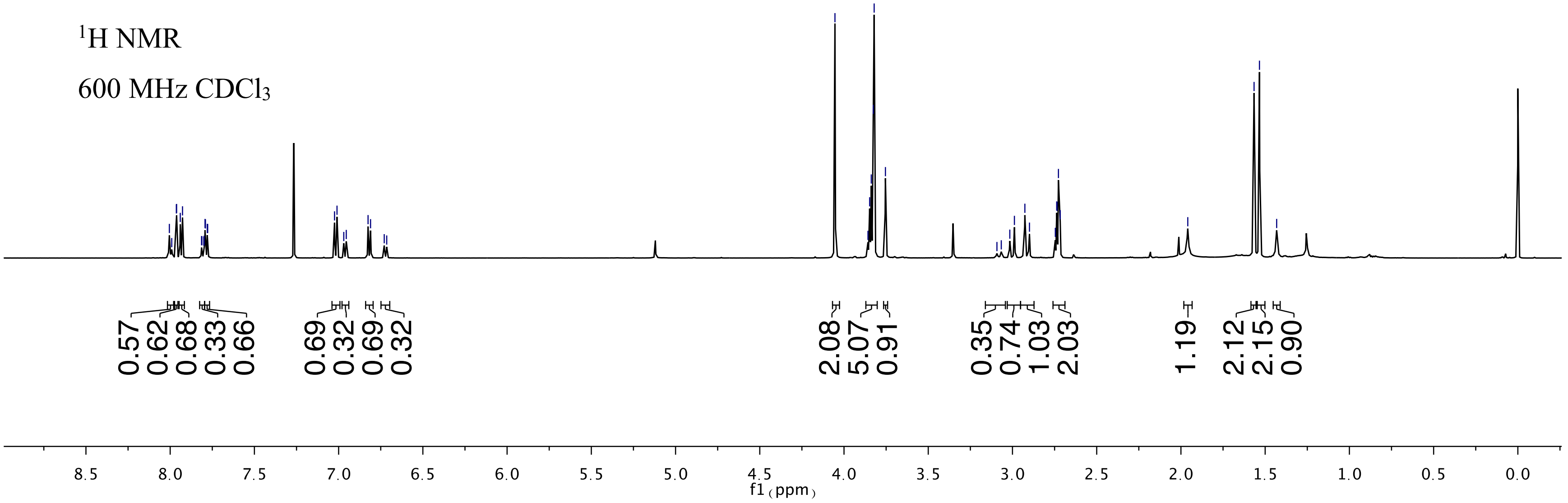

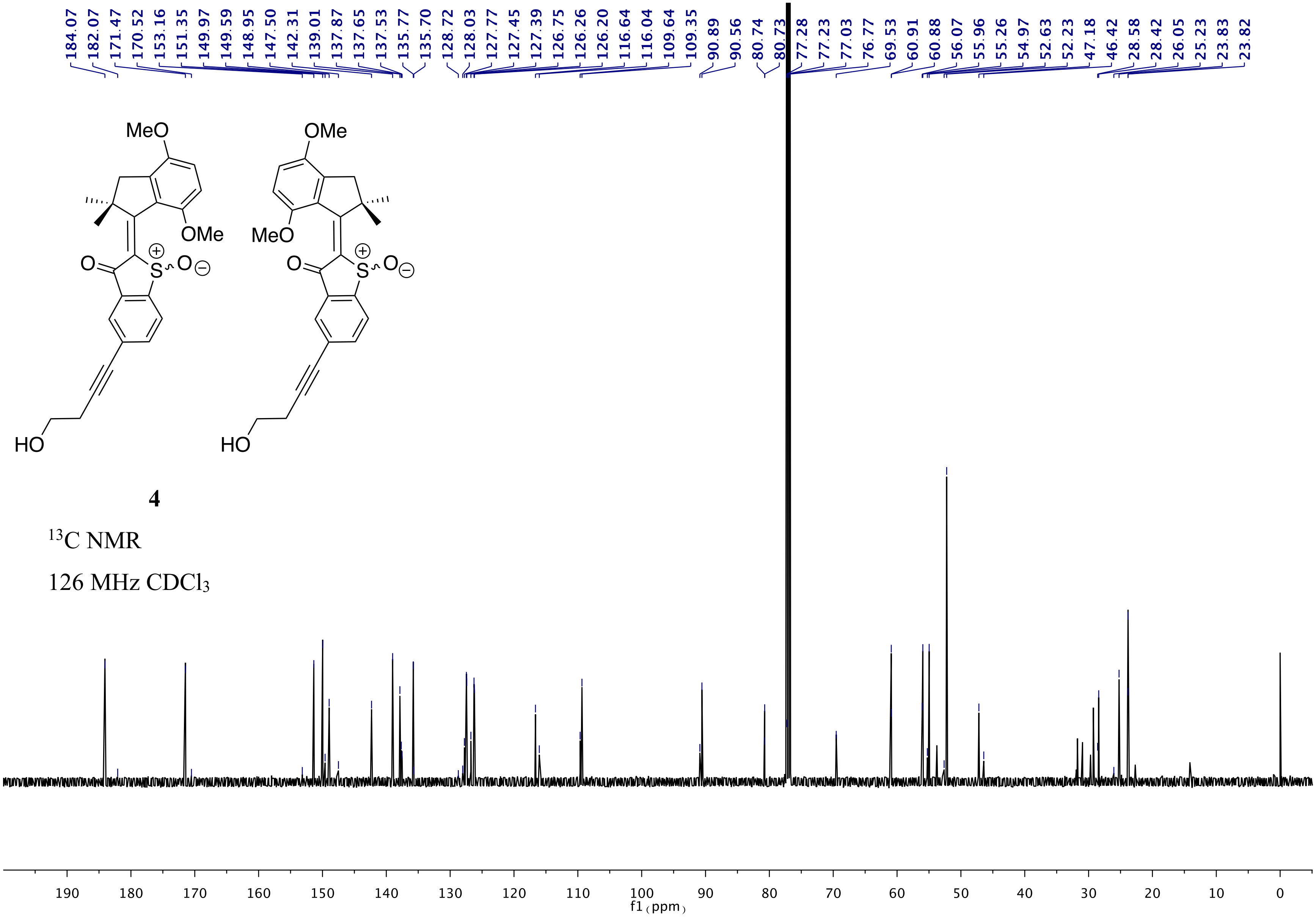

4

^{13}C NMR

126 MHz $CDCl_3$

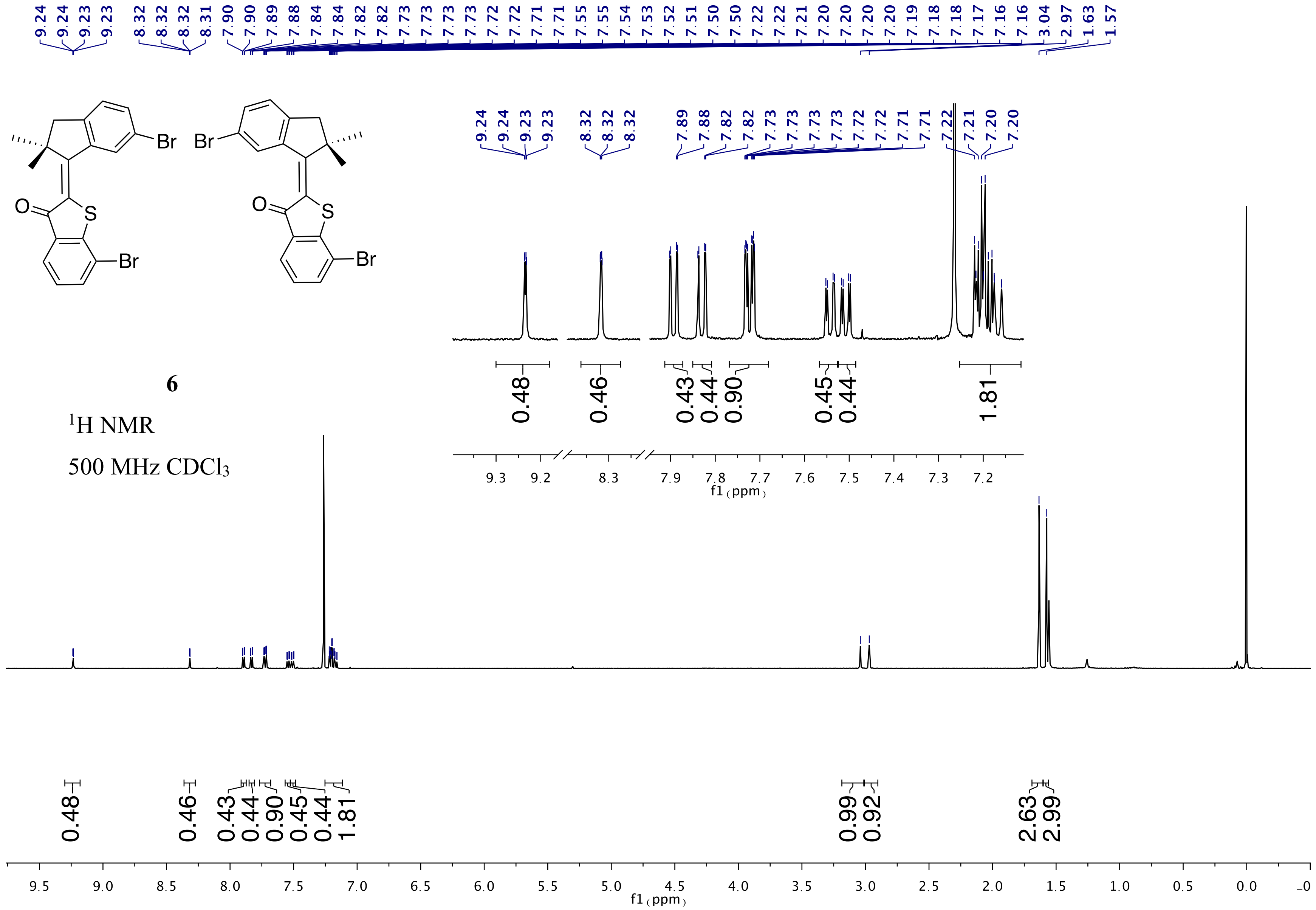
6
1H NMR
500 MHz CDCl3
f1 (ppm)

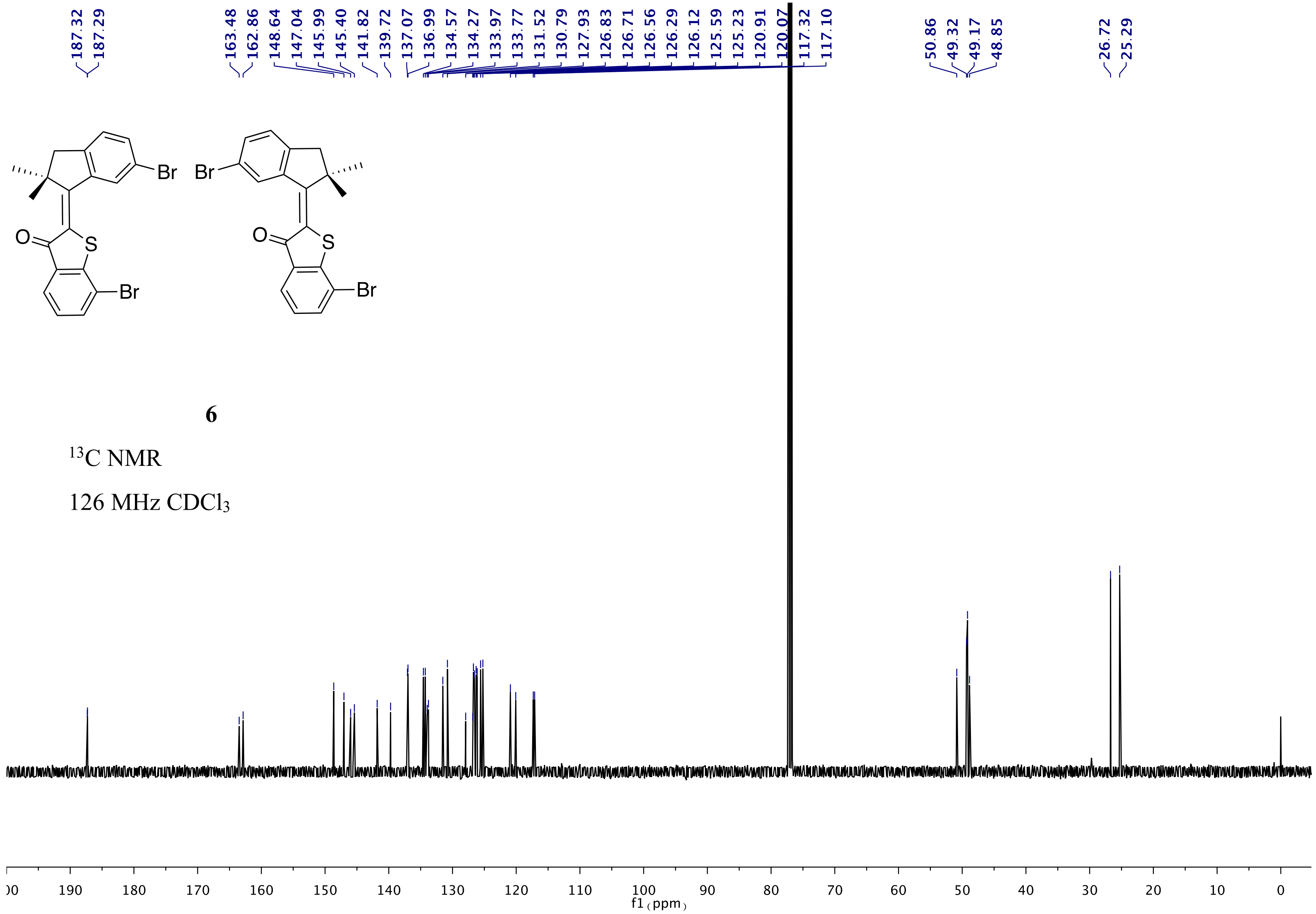

187.32
187.29
163.48
162.86
148.64
147.04
145.99
145.40
141.82
139.72
137.07
136.99
134.57
134.27
133.97
133.77
131.52
130.79
127.93
126.83
126.71
126.56
126.29
126.12
125.59
125.23
120.91
120.07
117.32
117.10
50.86
49.32
49.17
48.85
26.72
25.29
Br
Br
O
S
Br
O
S
Br
6
^{13}C NMR
126 MHz $CDCl_3$
f1 (ppm)

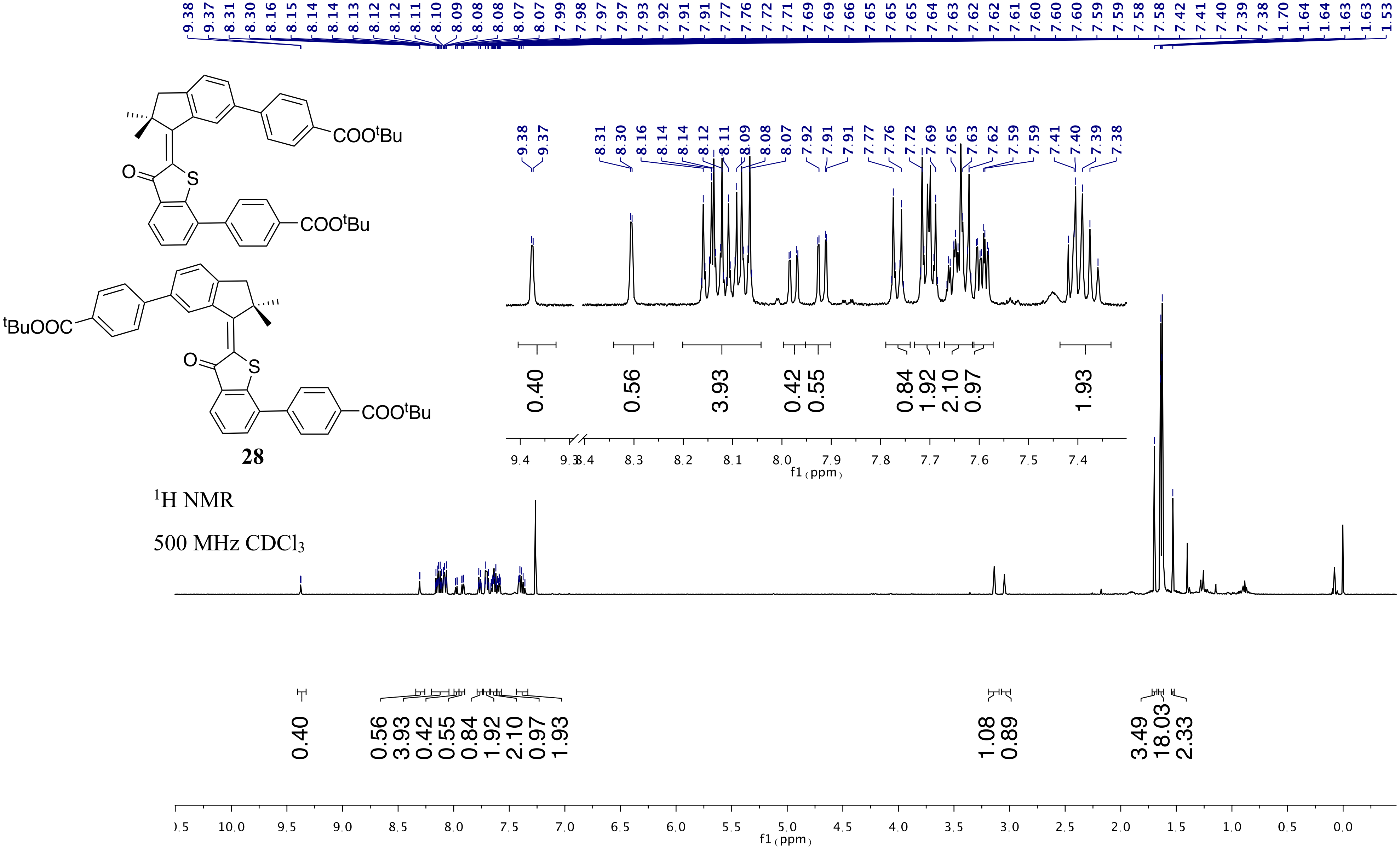

28

^{1}H NMR

500 MHz $CDCl_3$

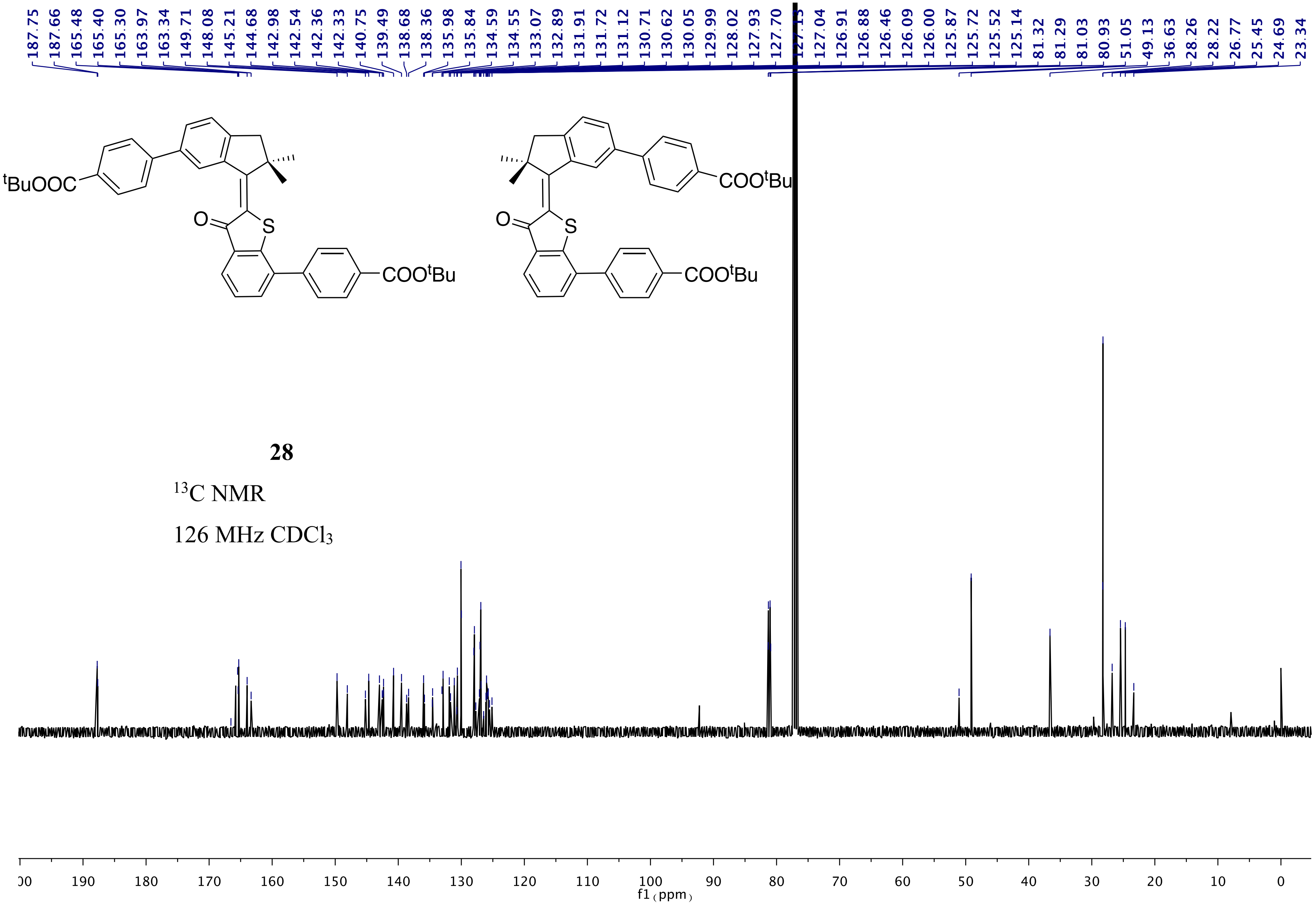
187.75
187.66
165.48
165.40
165.30
163.97
163.34
149.71
148.08
145.21
144.68
142.98
142.54
142.36
142.33
140.75
139.49
138.68
138.36
135.98
135.84
134.59
134.55
133.07
132.89
131.91
131.72
131.12
130.71
130.62
130.05
129.99
128.02
127.93
127.70
127.04
126.91
126.88
126.46
126.09
126.00
125.87
125.72
125.52
125.14
81.32
81.29
81.03
80.93
51.05
49.13
36.63
28.26
28.22
26.77
25.45
24.69
23.34
tBuOOC
O
S
COOtBu
COOtBu
COOtBu
28
13C NMR
126 MHz CDCl3
f1 (ppm)

7

^{1}H NMR

500 MHz DMSO-d6

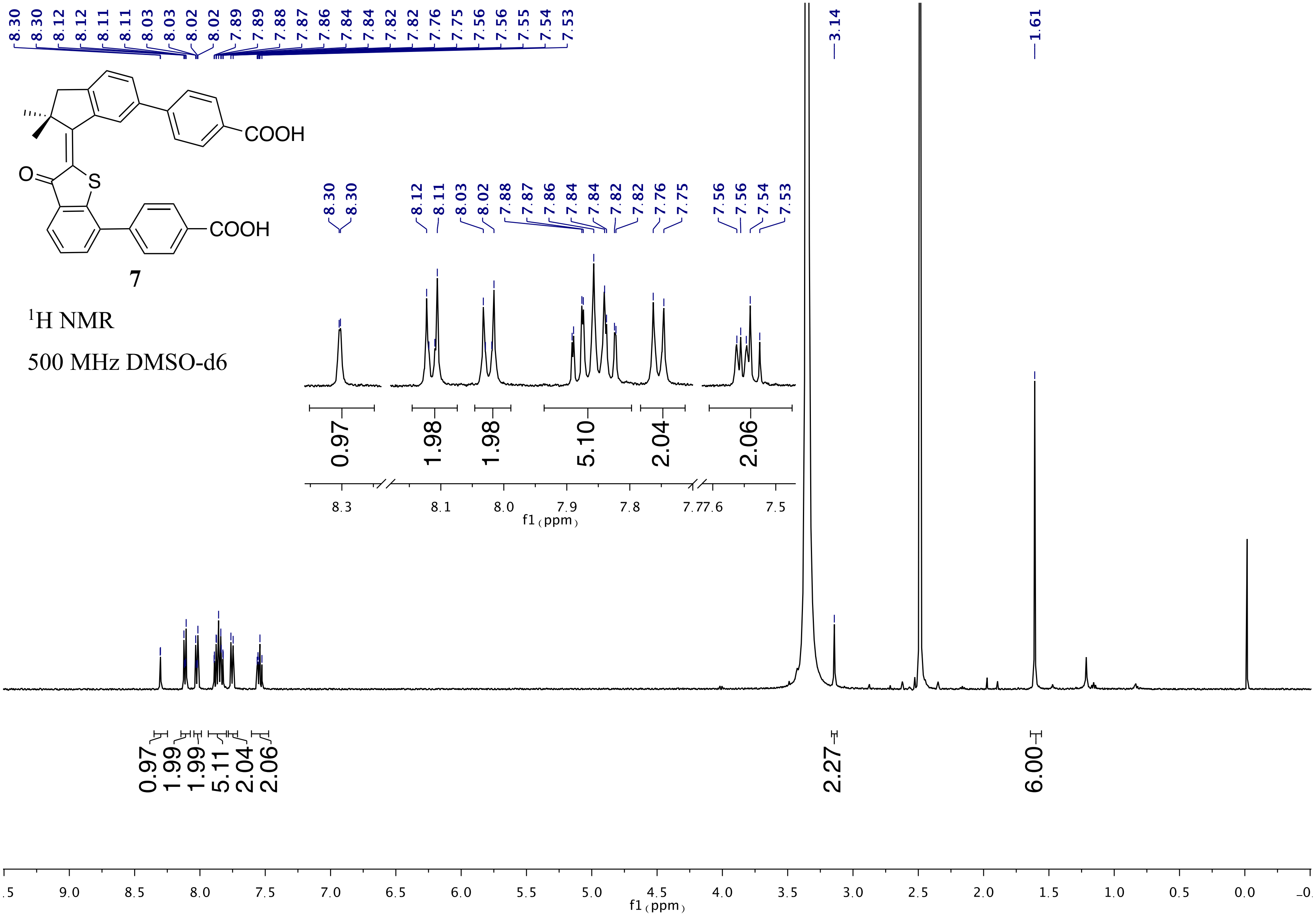

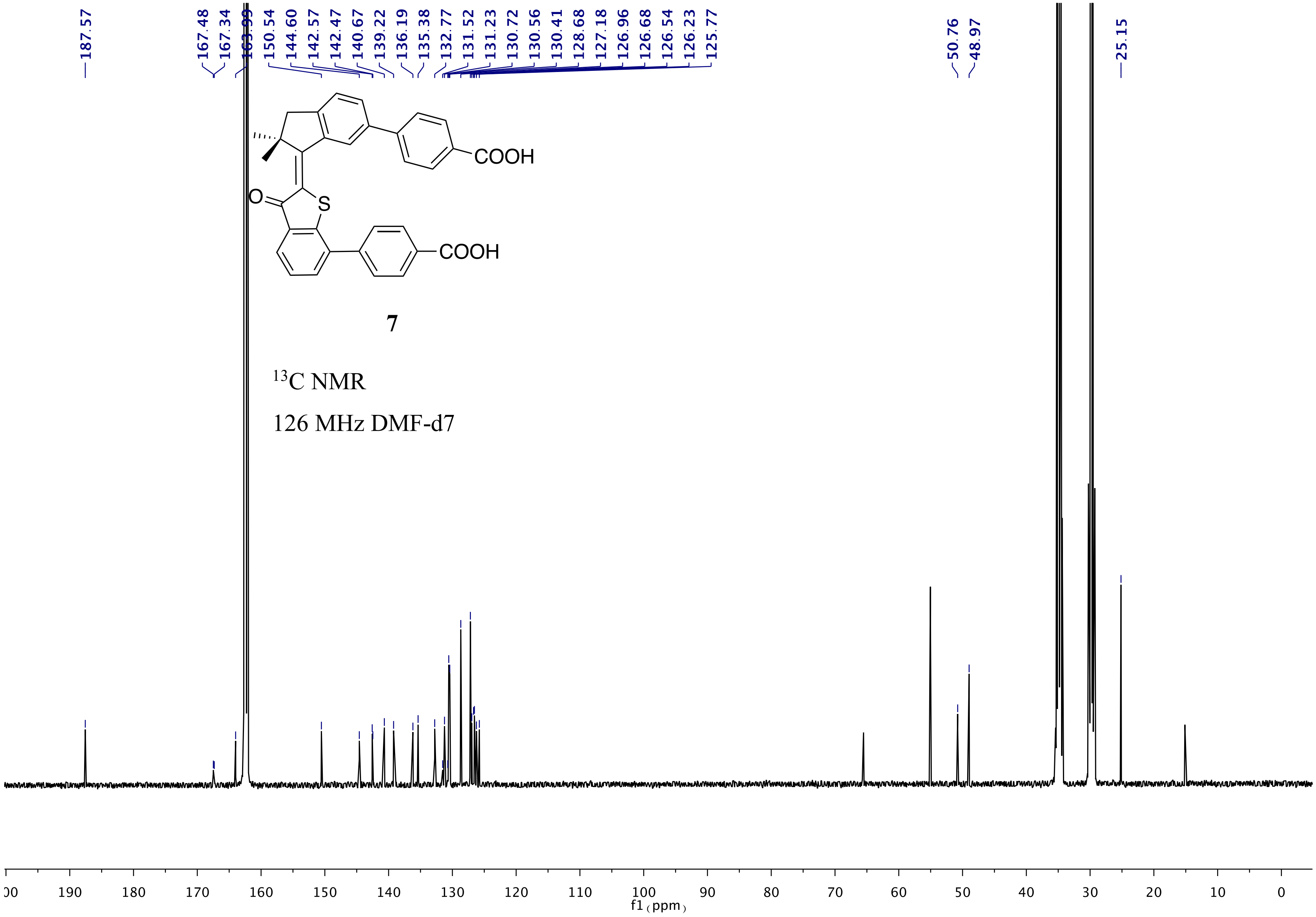
187.57
167.48
167.34
163.99
150.54
144.60
142.57
142.47
140.67
139.22
136.19
135.38
132.77
131.52
131.23
130.72
130.56
130.41
128.68
127.18
126.96
126.68
126.54
126.23
125.77
50.76
48.97
25.15
COOH
COOH
O
S
7
^{13}C NMR
126 MHz DMF-d7
f1 (ppm)

D. Additional characterization of *E* and *Z* isomers of 2

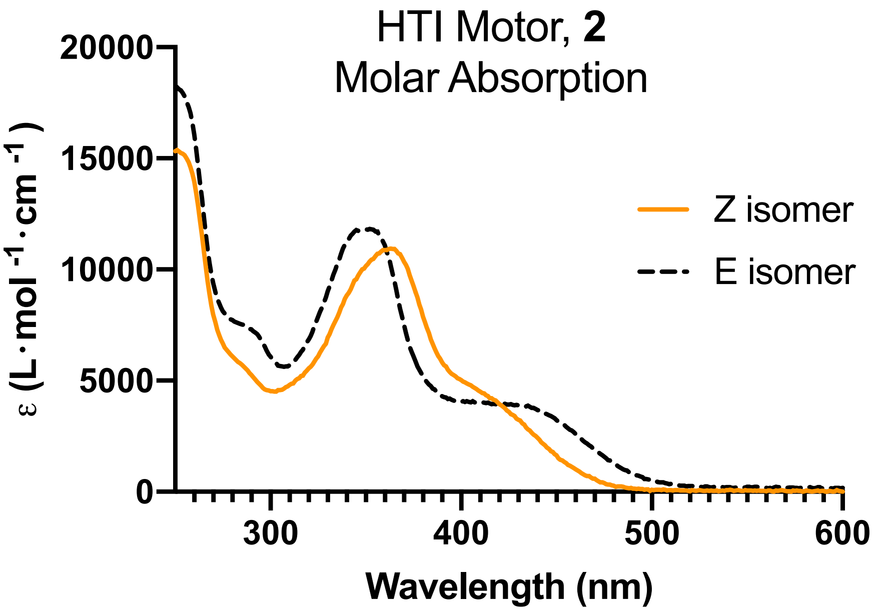

Figure S8. Extinction coefficient for the *E* and *Z* isomers of **2** in $CHCl_3$.

2-*E*: $\varepsilon_{(455\ nm)}$ = 2,738 L· mol^{-1} ·cm^{-1}

2-*Z*: $\varepsilon_{(455\ nm)}$ = 1,255 L· mol^{-1} ·cm^{-1}

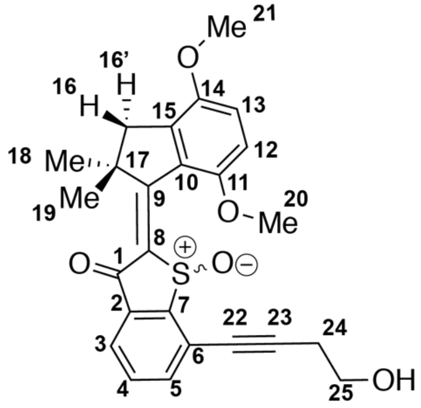

Figure S9. Structure of HTI **2-*Z***. The numbers in the figure correspond to the numbers on the spectra below. ^{1}H NMR (500 MHz, $CDCl_3$) 7.87 (dd, J_1 = 7.7 Hz, J_2 = 1.1 Hz, 1H, C-H(3)), 7.69 (dd, J_1 = 7.6 Hz, J_2 = 1.2 Hz, 1H, H-C(5)), 7.61 (app t, J = 7.6 Hz, 1H, H-C(4)), 7.01 (d, J = 8.9 Hz, 1H, H-C(13)), 6.82 (d, J = 8.9 Hz, 1H, H-C(12)), 4.90 (br s, 1H), 4.09 (s, 3H, H_3-C(20)), 3.82 (s, 3H, H_3-C(21)), 3.88-3.76 (br m, 2H, H_2-C(25)), 3.00 (d, J = 16.6 Hz, 1H, H_2-C(16)), 2.92 (d, J = 16.7 Hz, 1H, H_2-C(16')), 2.74 (ddd, J_1= 17.2, J_2= 8.6, J_3=4.1 Hz, 1H, H_2-C(24)), 2.60 (ddd, J_1=17.2, J_2=5.1, J_3=3.1 Hz, 1H, H_2-C(24)), 1.59 (s, 3H, H_3-C(18)), 1.55 (s, 3H, H_3-C(19)) ppm. ^{13}C NMR (126 MHz, $CDCl_3$) δ 183.84 (C(1)), 172.23 (C(9)), 151.49 (C(11)), 149.96 (C(14 or 7)), 149.93 (C(14 or 7)), 140.02 (C(8)), 139.05 (C(15)), 136.52 (C(5)), 136.31 (C(2)), 131.97 (C(4)), 126.01 (C(10)), 123.75 (C(6)), 123.52 (C(3)), 116.91 (C(13)), 109.40 (C(12)), 99.76 (C(23)), 76.98 (C(22)), 61.10 (C(25)), 55.97 (C(21)), 54.74 (C(20)), 52.09 (C(17)), 47.24 (C(16)), 28.40 (C(18)), 25.30 (C(19)), 24.88 (C(24)) ppm. Only relative, not absolute, assignments for protons 18/19 and 16/16' were determined.

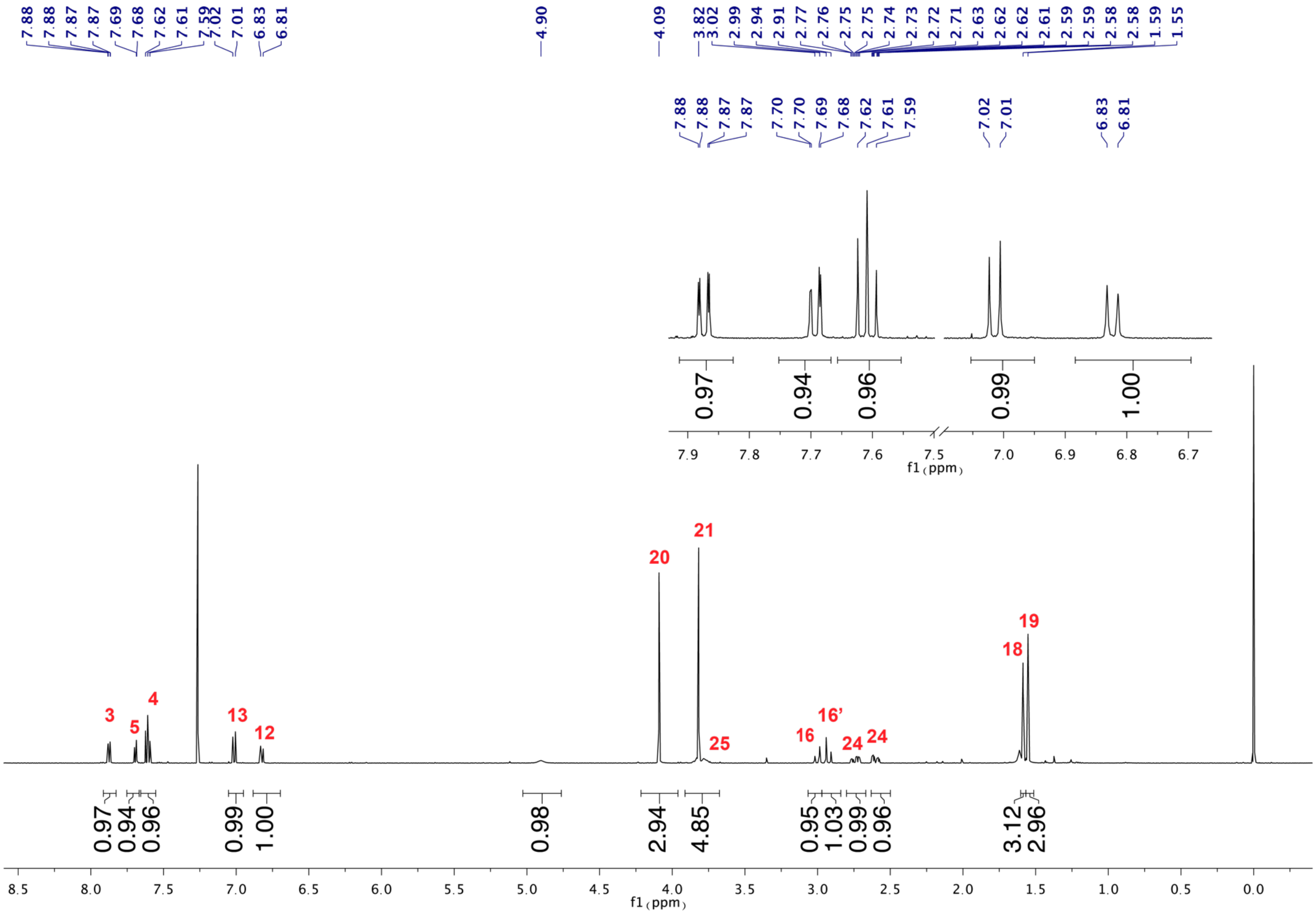

Figure S10. ^{1}H NMR (500 MHz, $CDCl_3$) and assignments that correspond to **Figure S9** for HTI **2-*Z***.

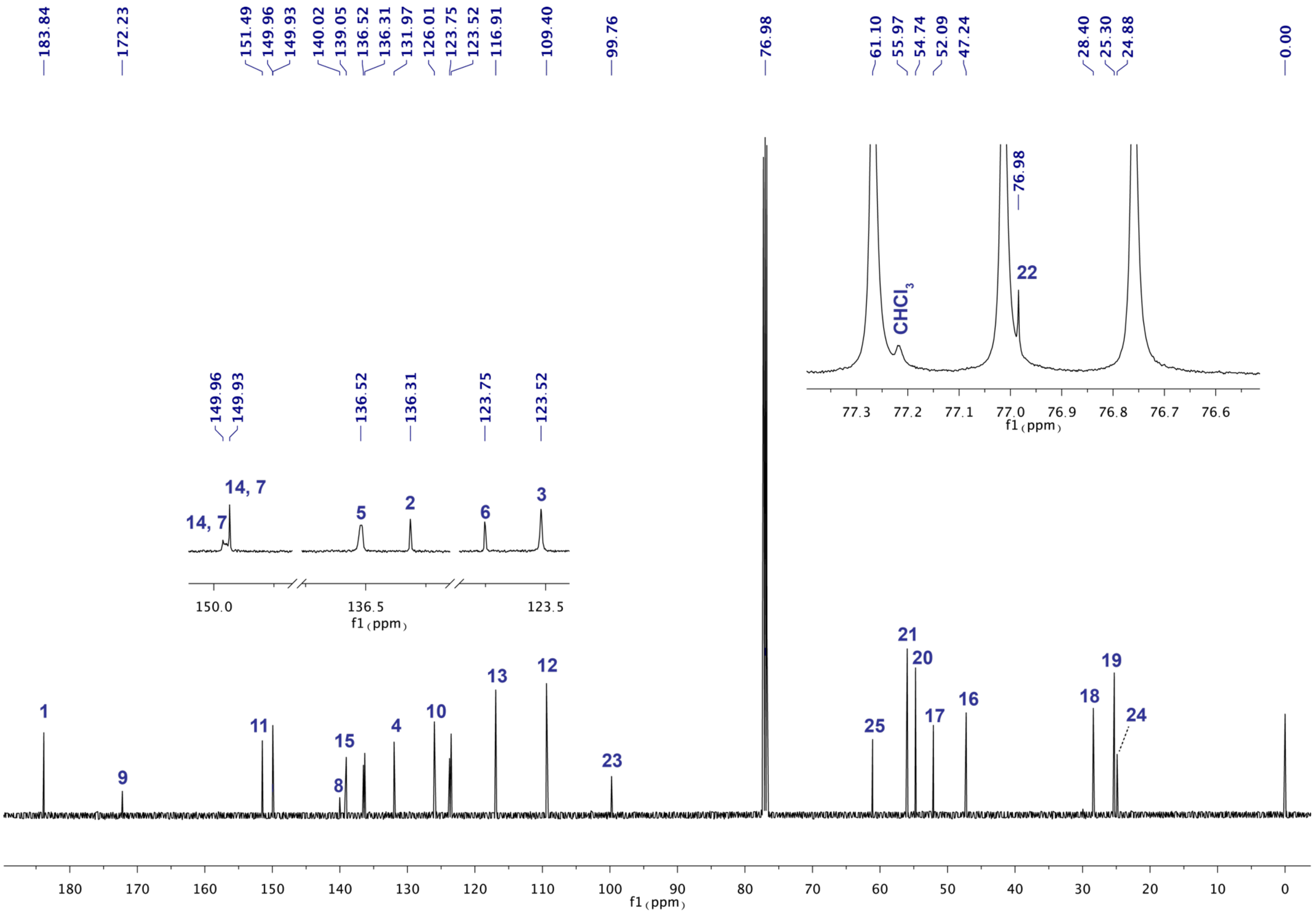

Figure S11. ${}^{13}C$ NMR (126 MHz, $CDCl_3$) and assignments that correspond **Figure S9** for HTI **2-*Z***.

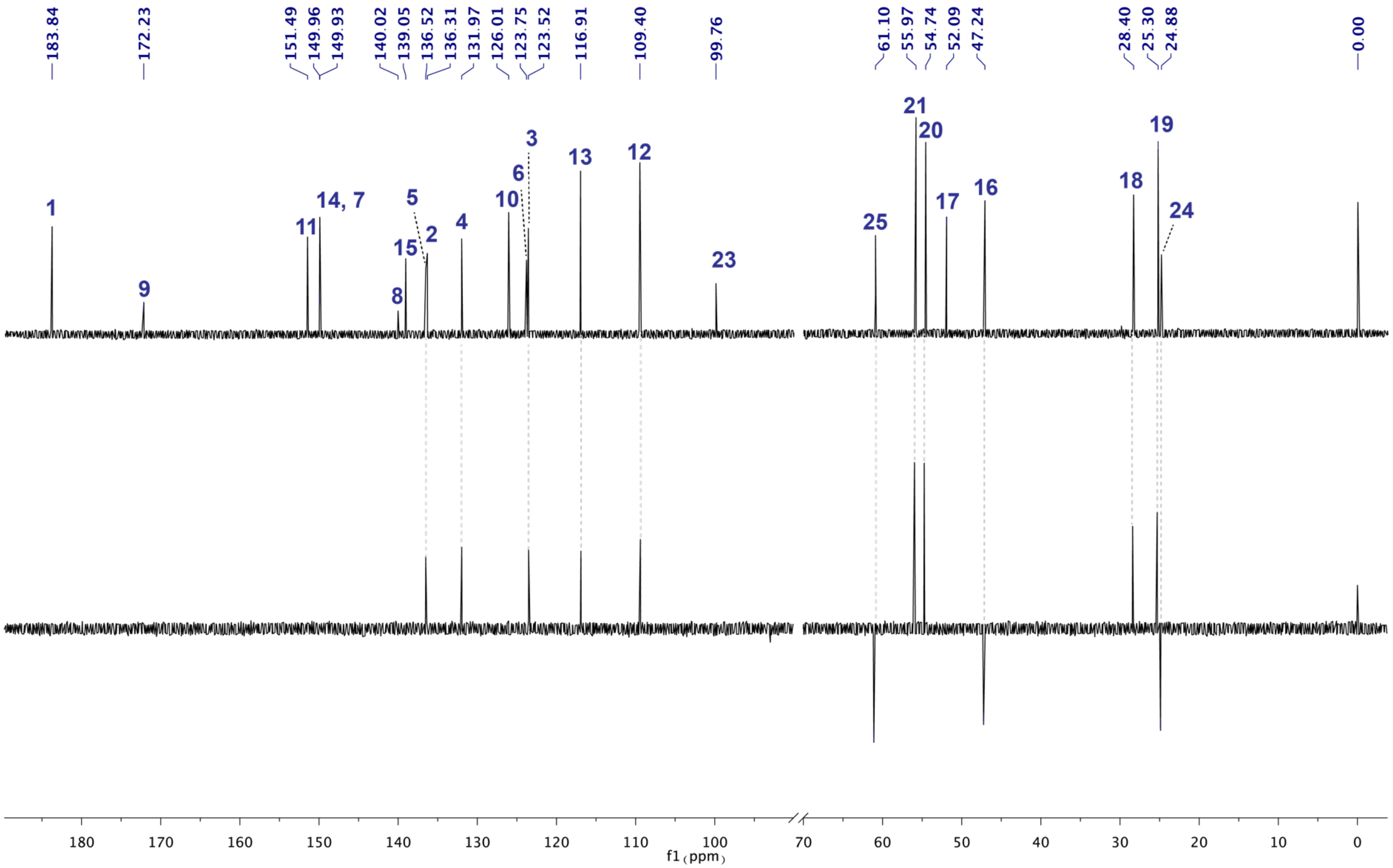

Figure S12. Top: ^{13}C NMR spectrum of **2-*Z*** Bottom: DEPT-135 ^{13}C NMR spectrum of **2-*Z*** (126 MHz, $CDCl_3$).

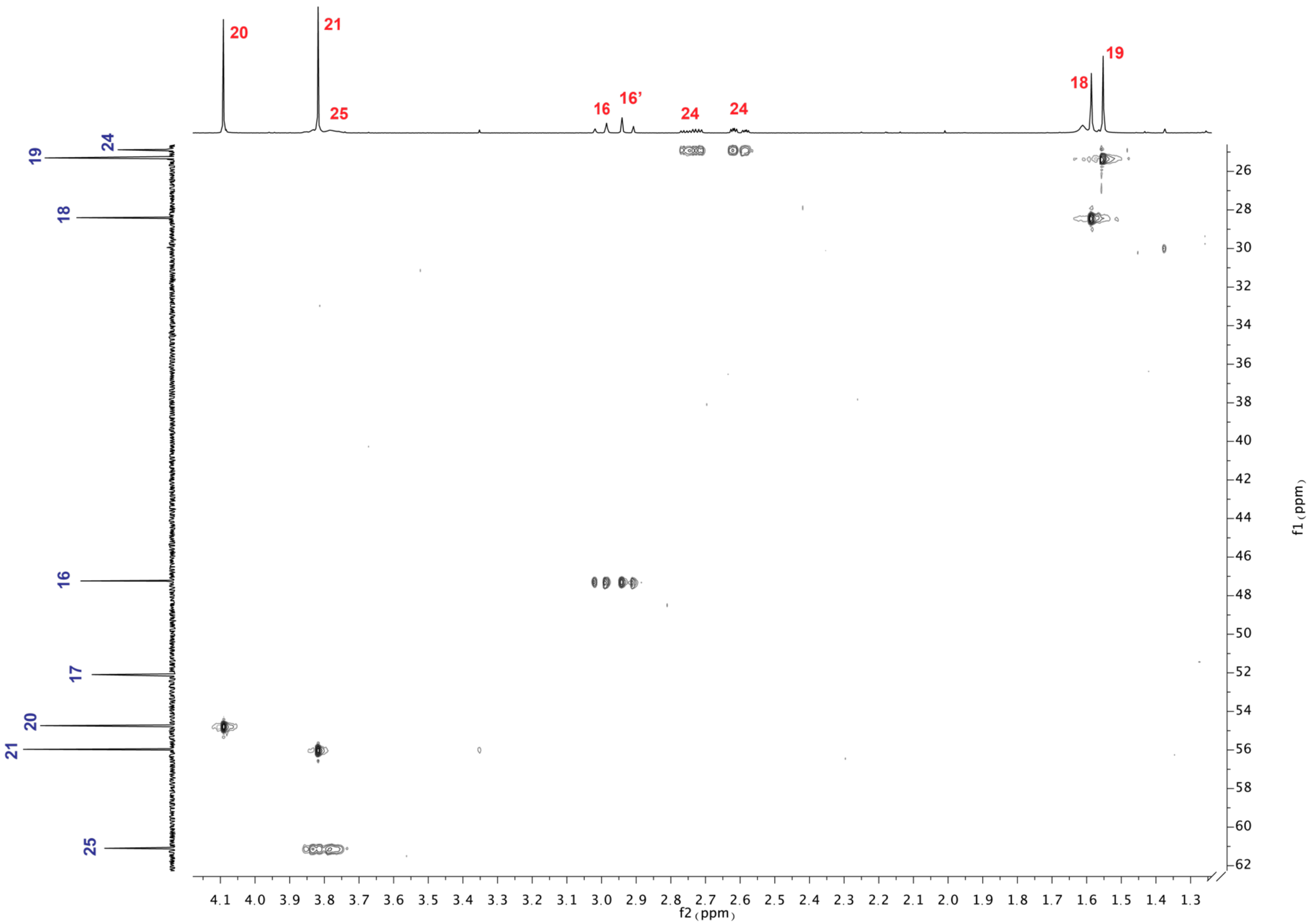

Figure S13. Selected region of HSQC spectrum of HTI **2-*Z*** (600 MHz, $CDCl_3$) and assignments that correspond to **Figure S9.**

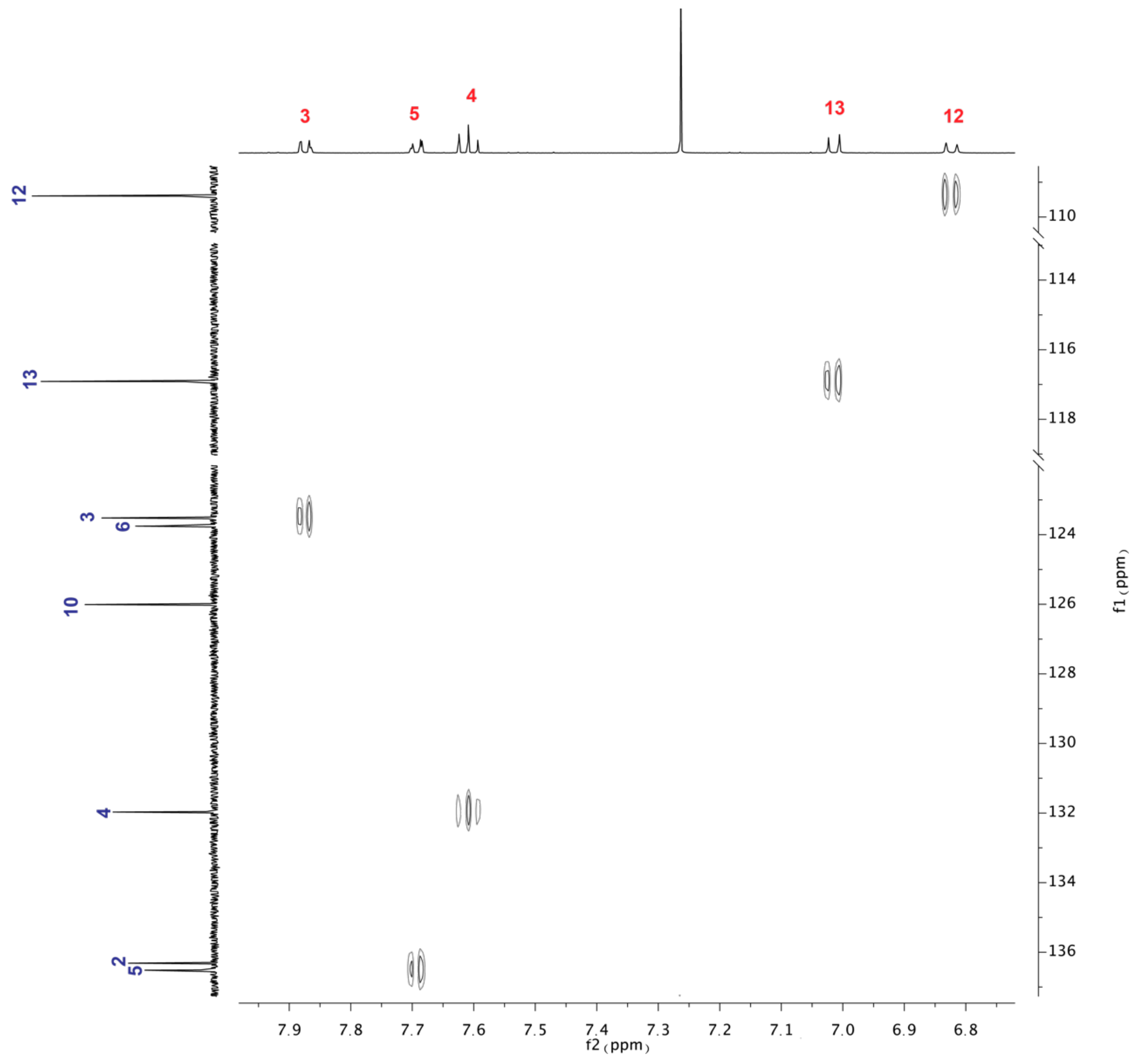

Figure S14. Selected region of HSQC spectrum of HTI **2-*Z*** (600 MHz, $CDCl_3$) and assignments that correspond to **Figure S9.**

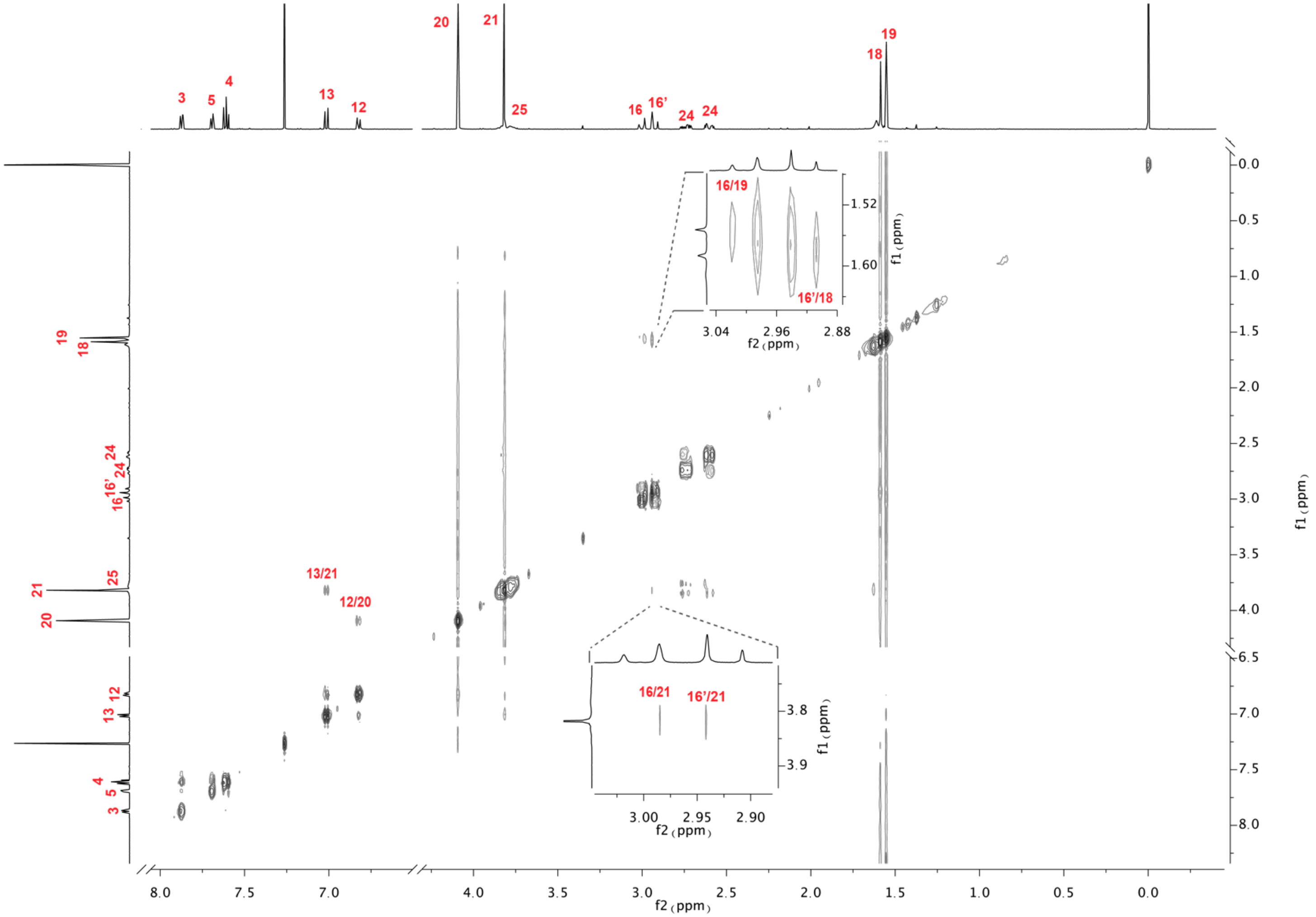

Figure S15. NOESY NMR Spectrum (500 MHz, $CDCl_3$, 0.3 s mixing time) of HTI **2-*Z***. The cross-peaks between protons 12/20, 13/21, 16 /21, and 16’/21 were used to assign methoxy protons. The cross-peaks between protons 16/19 and 16’/18 were used to determine their relative assignments shown in **Figure S9.**

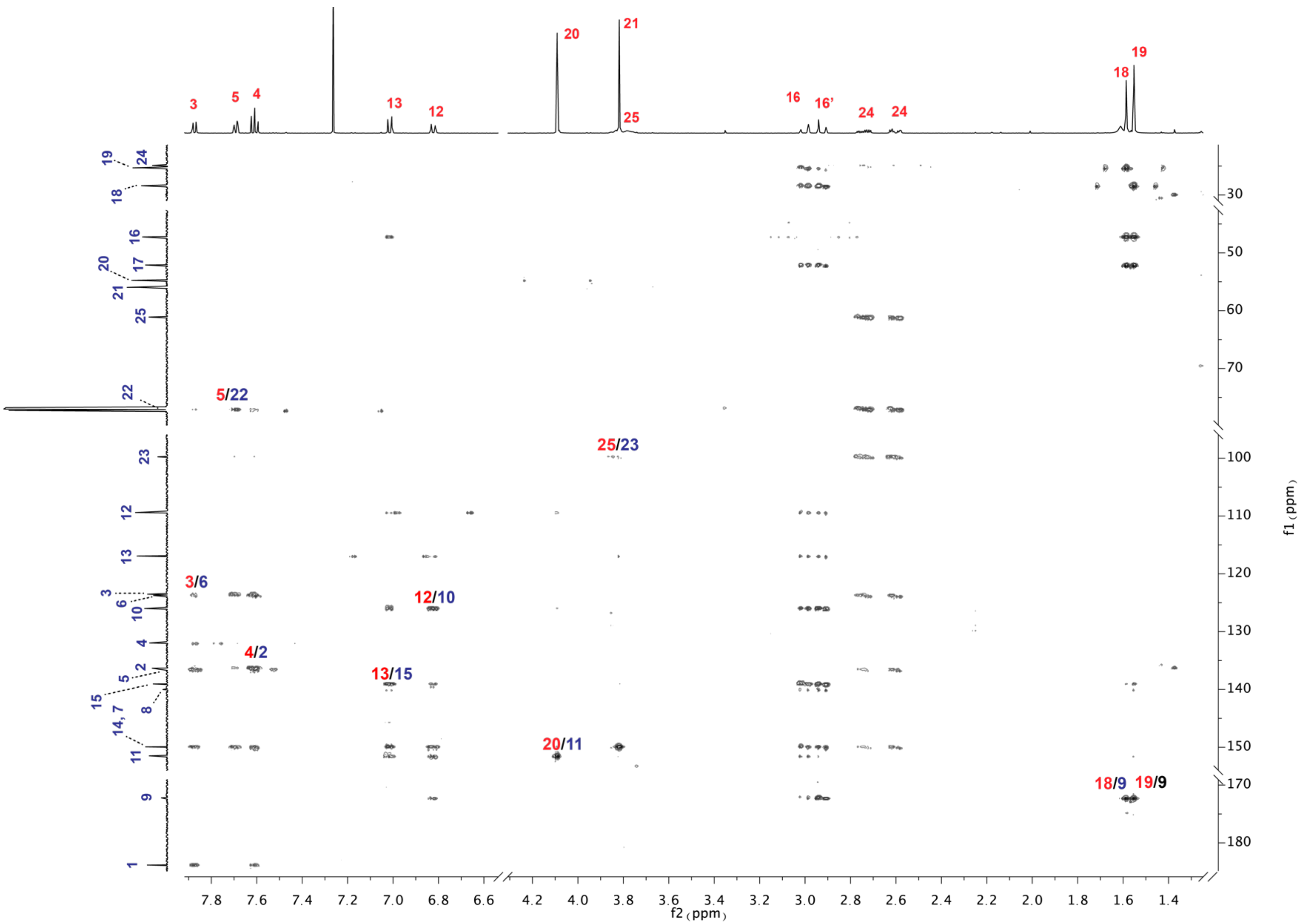

Figure S16. HMBC spectrum of HTI **2-*Z*** (600 MHz, $CDCl_3$) and assignments that correspond to **Figure S9.** Contours that were used to aid in assignments are noted.

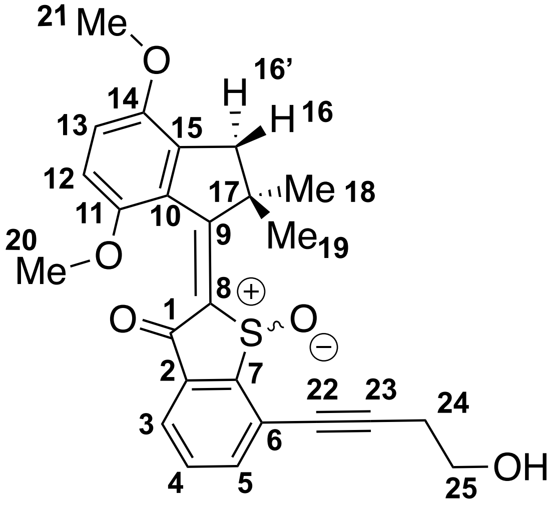

Figure S17. Structure of HTI **2-*E***. The numbers in the figure correspond to the numbers on the spectra below. ^{1}H NMR (500 MHz, $CDCl_3$) 7.92 (dd, J_1 = 7.6 Hz, J_2 = 1.1 Hz, 1H, C-H(3)), 7.72 (dd, J_1 = 7.6 Hz, J_2 = 1.1 Hz, 1H, C-H(5)), 7.65 (app t, J = 7.6 Hz, 1H, C-H(4)), 6.97 (d, J = 8.6 Hz, 1H, H-C(13)), 6.73 (d, J = 8.7, 1H, H-C(12)), 4.79 (br s, 1H), 3.93-3.91 (br m, 2H, H_2-C(25)), 3.83 (s, 3H, H_3-C(21)), 3.74 (s, 3H, H_3-C(20)), 3.08 (d, J = 15.7 Hz, 1H, H_2-C(16)), 2.92 (d, J = 15.7 Hz, 1H, H_2-C(16’)), 2.79 (ddd, J_1= 17.2, J_2= 6.9, J_3=4.8 Hz, 1H, H2-C(24)), 2.70 (ddd, J_1=17.2, J_2=5.4, J_3=4.1 Hz, 1H, H2-C(24)), 1.95 (s, 3H, H_3-C(19)), 1.42 (s, 3H, H_3-C(18)) ppm. ^{13}C NMR (126 MHz, $CDCl_3$) δ 181.84 (C(1)), 171.05 (C(9)), 153.25 (C(11)), 149.64 (C(14)), 148.90 (C(7)), 137.80 (C(15)), 137.44 (C(8)), 136.23 (C(2)), 136.21 (C(5)), 132.55 (C(4)), 128.83 (C(10)), 124.14 (C(6)), 123.94 (C(3)), 116.14 (C(13)), 109.73 (C(12)), 100.03 (C(23)), 77.00 (C(22)), 61.24(C(25)), 56.08 (C(21)), 55.36 (C(20)), 52.95 (C(17)), 46.44 (C(16)), 28.63 (C(18)), 26.07 (C(19)), 24.85 (C(24)) ppm. Only relative, not absolute, assignments for protons 18/19 and 16/16' were determined.

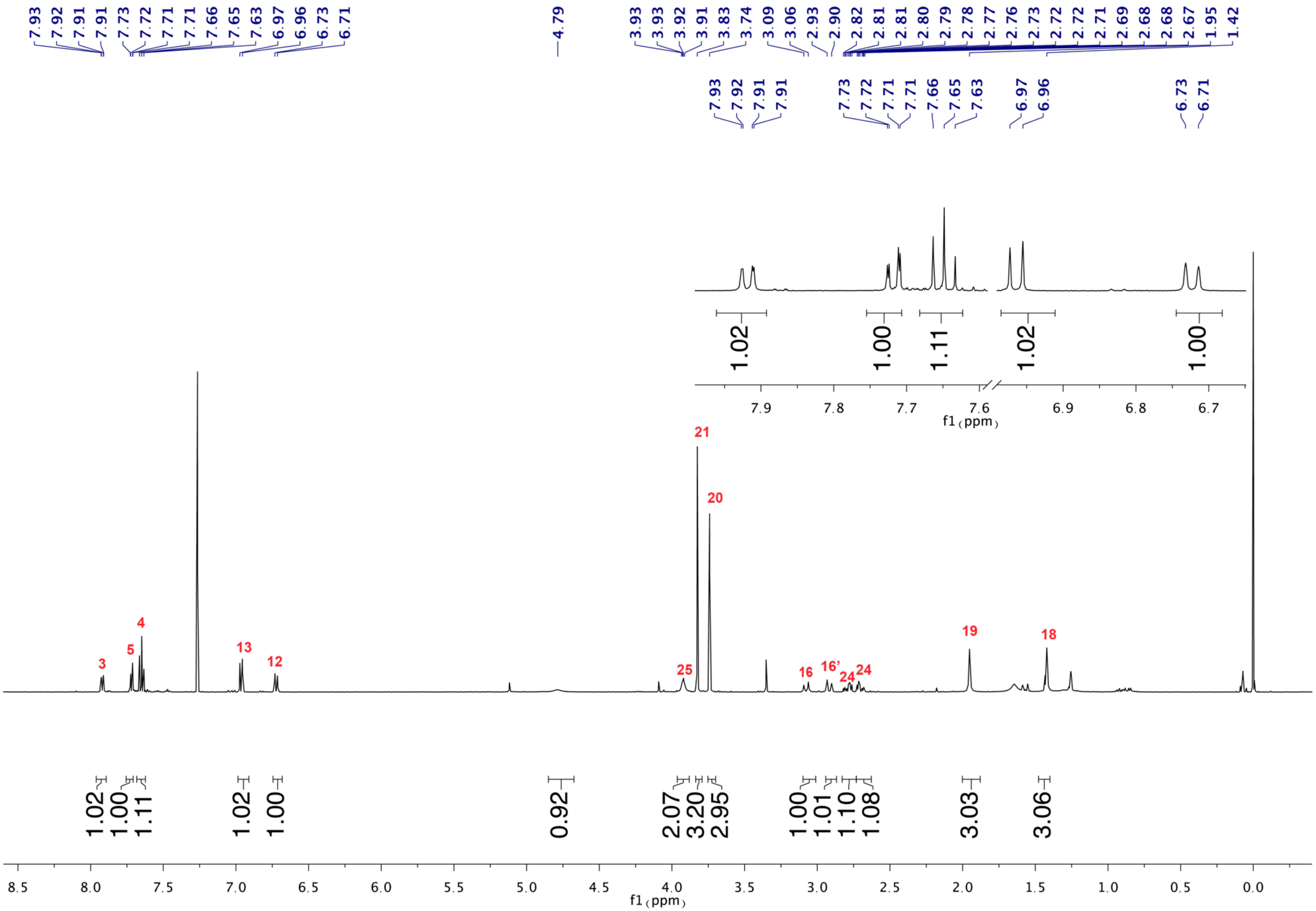

Figure S18. ^{1}H NMR (500 MHz, $CDCl_3$) and assignments that correspond to **Figure S17** for HTI **2-*E***.

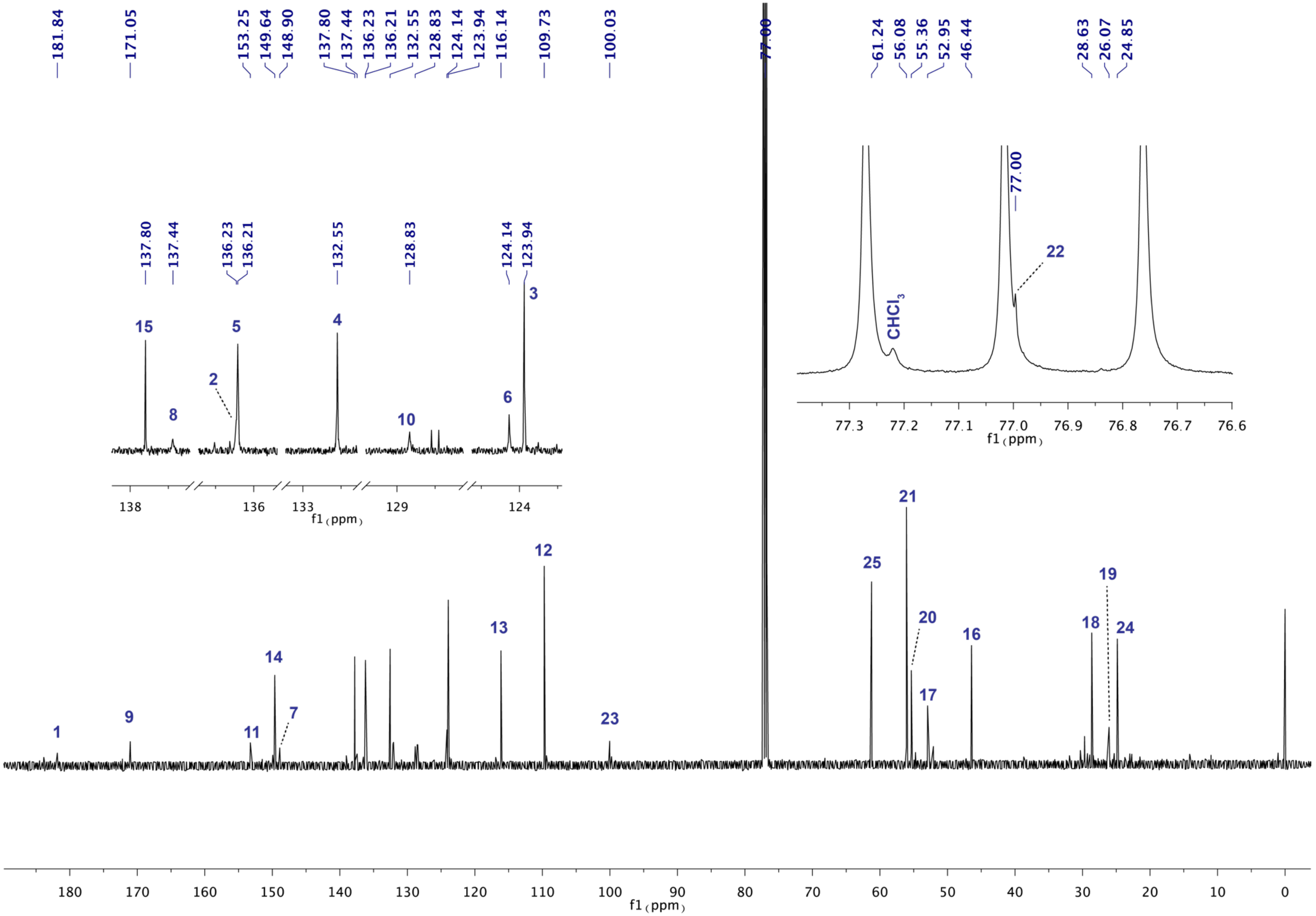

Figure S19. ^{13}C NMR (126 MHz, $CDCl_3$) and assignments that correspond to **Figure S17** for HTI **2-*E***.

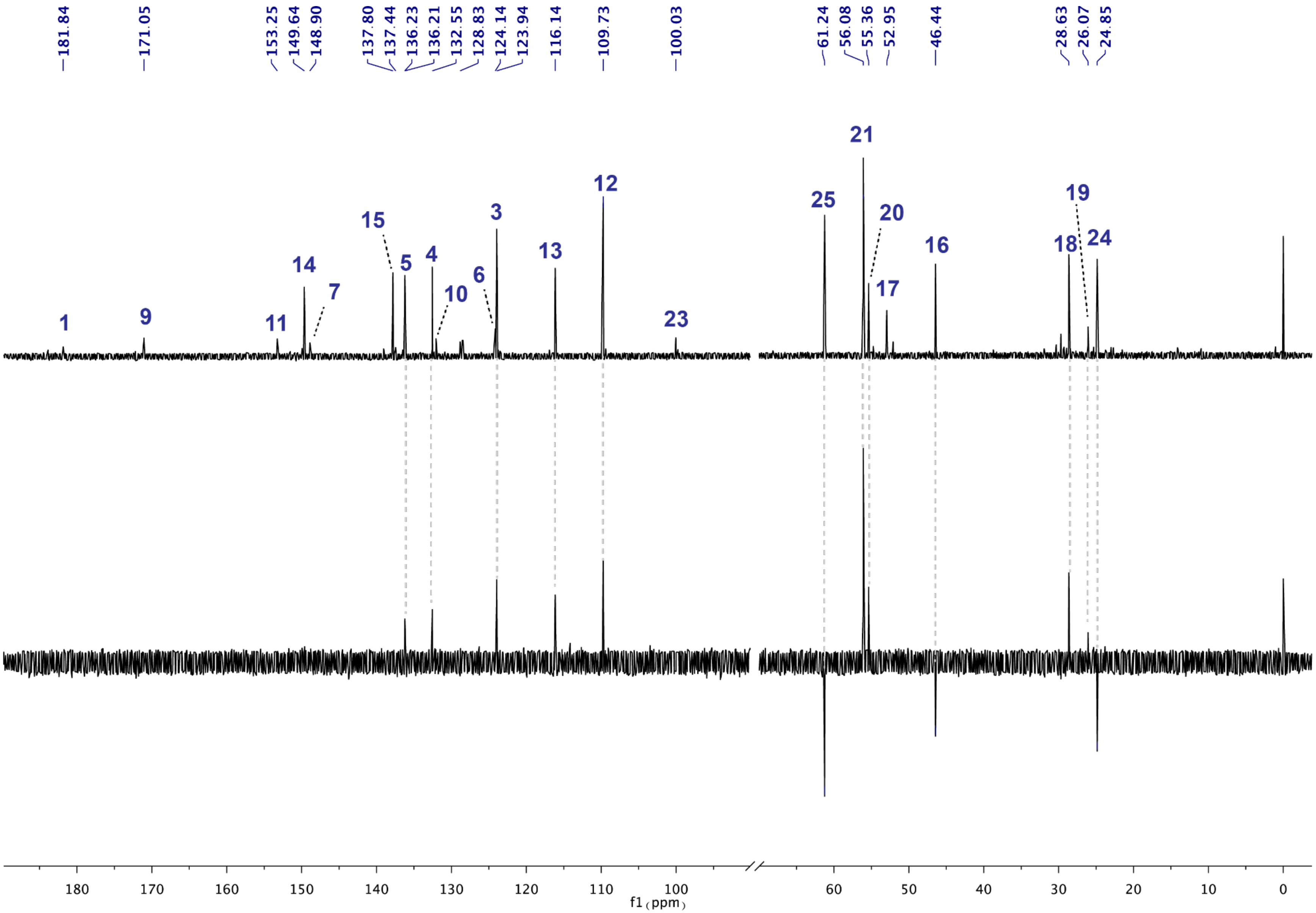

Figure S20. Top: ^{13}C NMR spectrum of **2-E** Bottom: DEPT-135 ^{13}C NMR spectrum of **2-*E*** (126 MHz, $CDCl_3$).

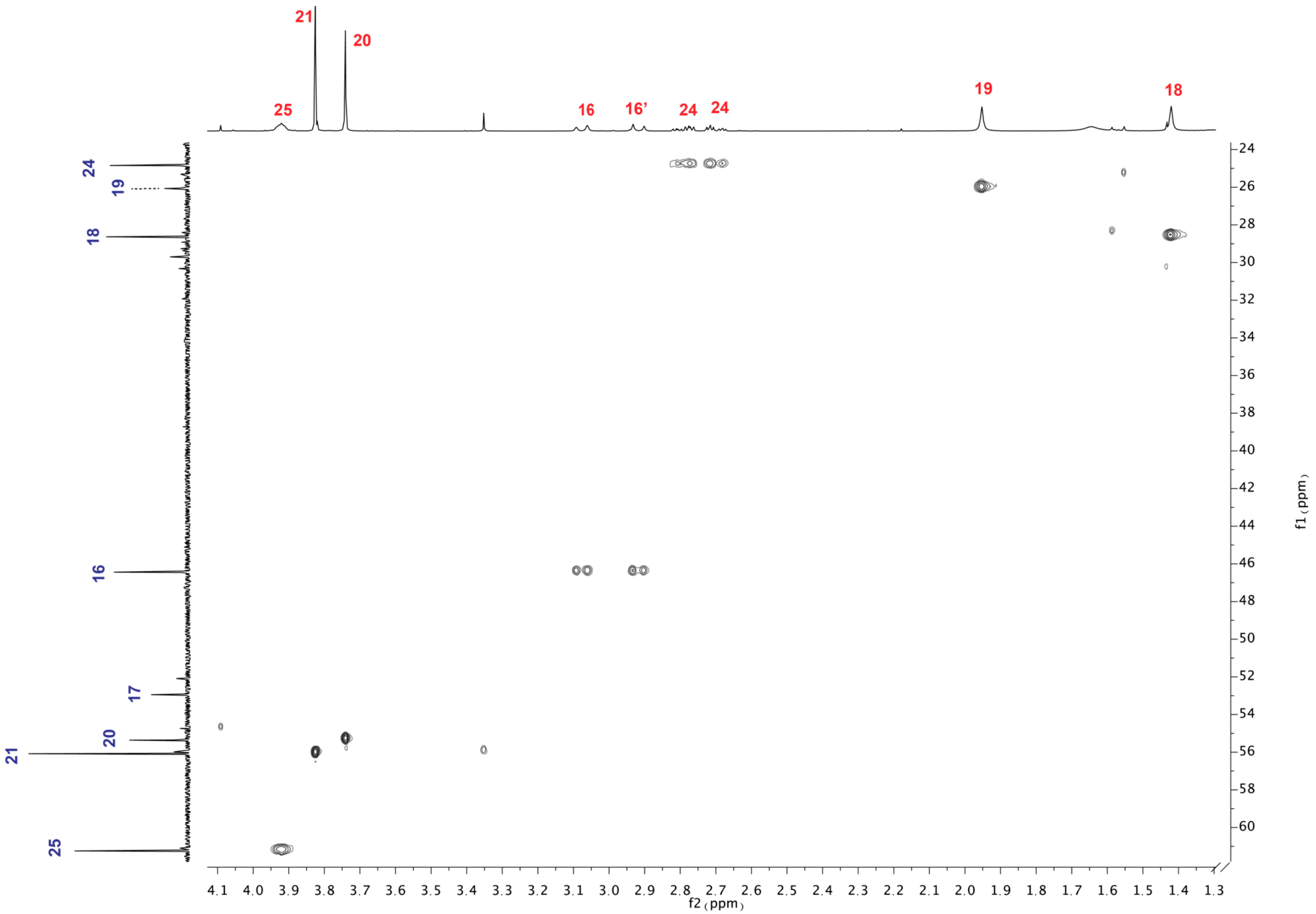

Figure S21. Selected region of HSQC spectrum of HTI **2-*E*** (600 MHz, $CDCl_3$) and assignments that correspond to **Figure S17.**

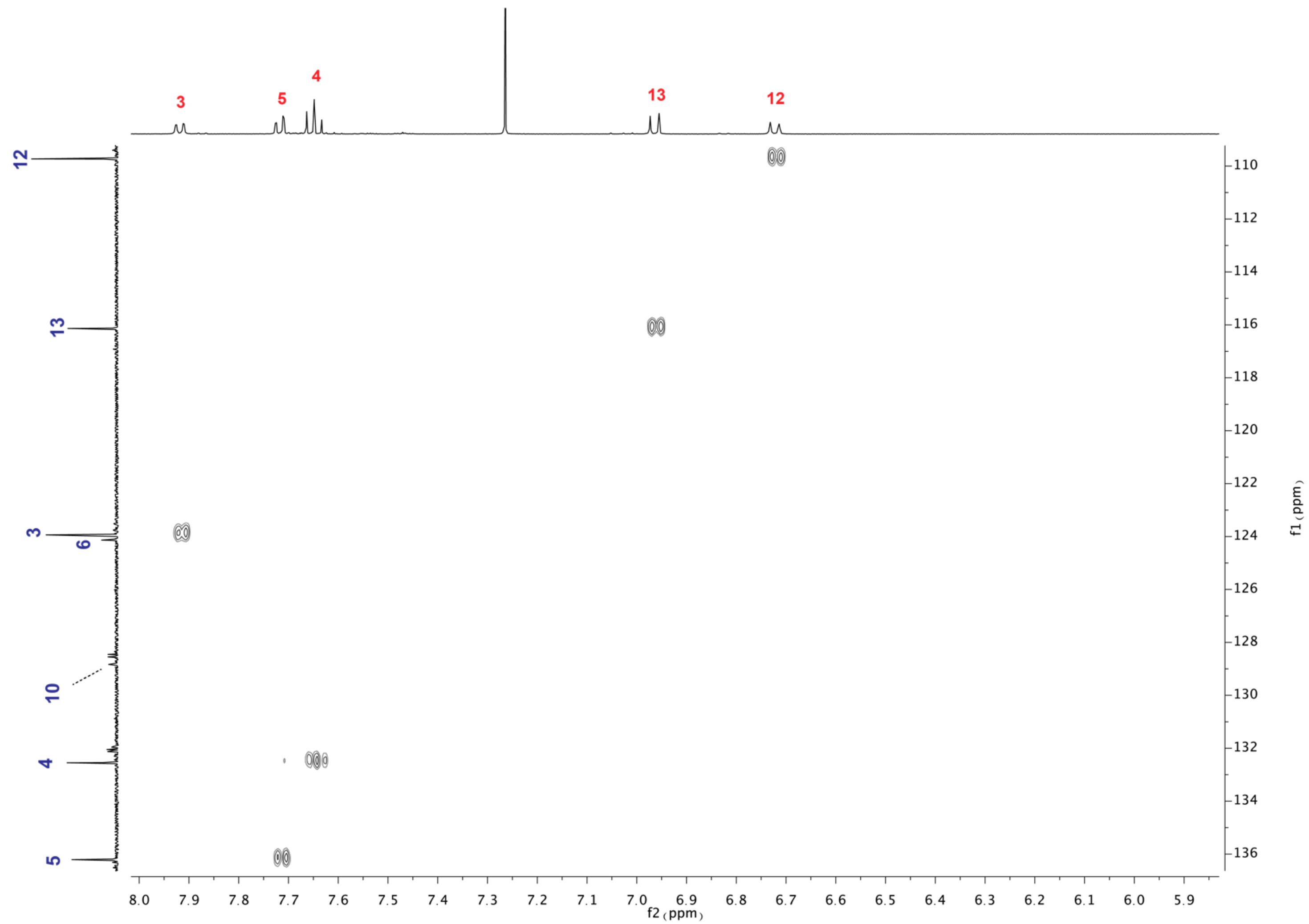

Figure S22. Selected region of HSQC spectrum of HTI **2-*E*** (600 MHz, $CDCl_3$) and assignments that correspond to **Figure S17.**

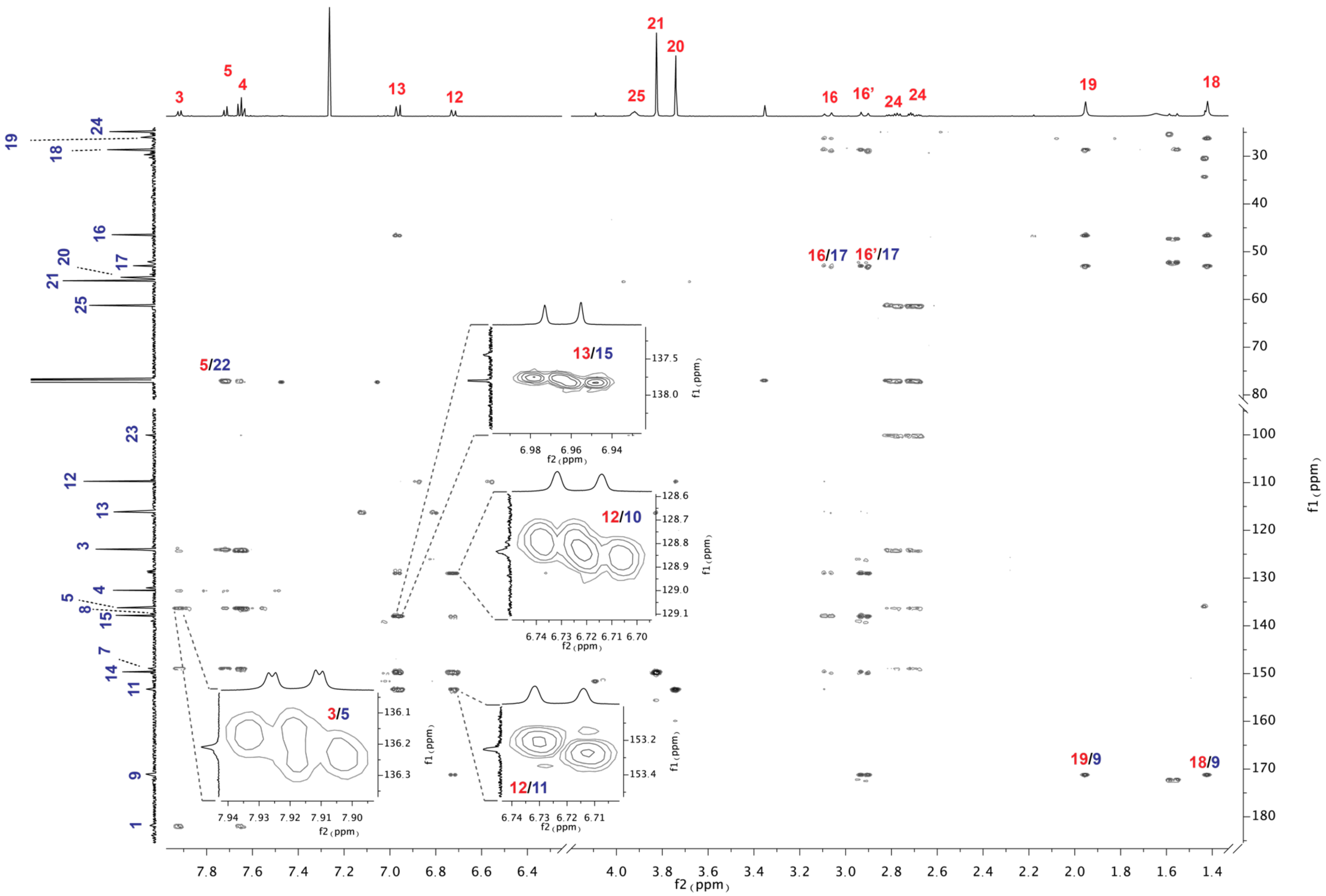

Figure S23. Selected region of HMBC spectrum of HTI **2-*E*** (600 MHz, $CDCl_3$) and assignments that correspond to **Figure S17.** Contours that were used to aid in assignments are noted.

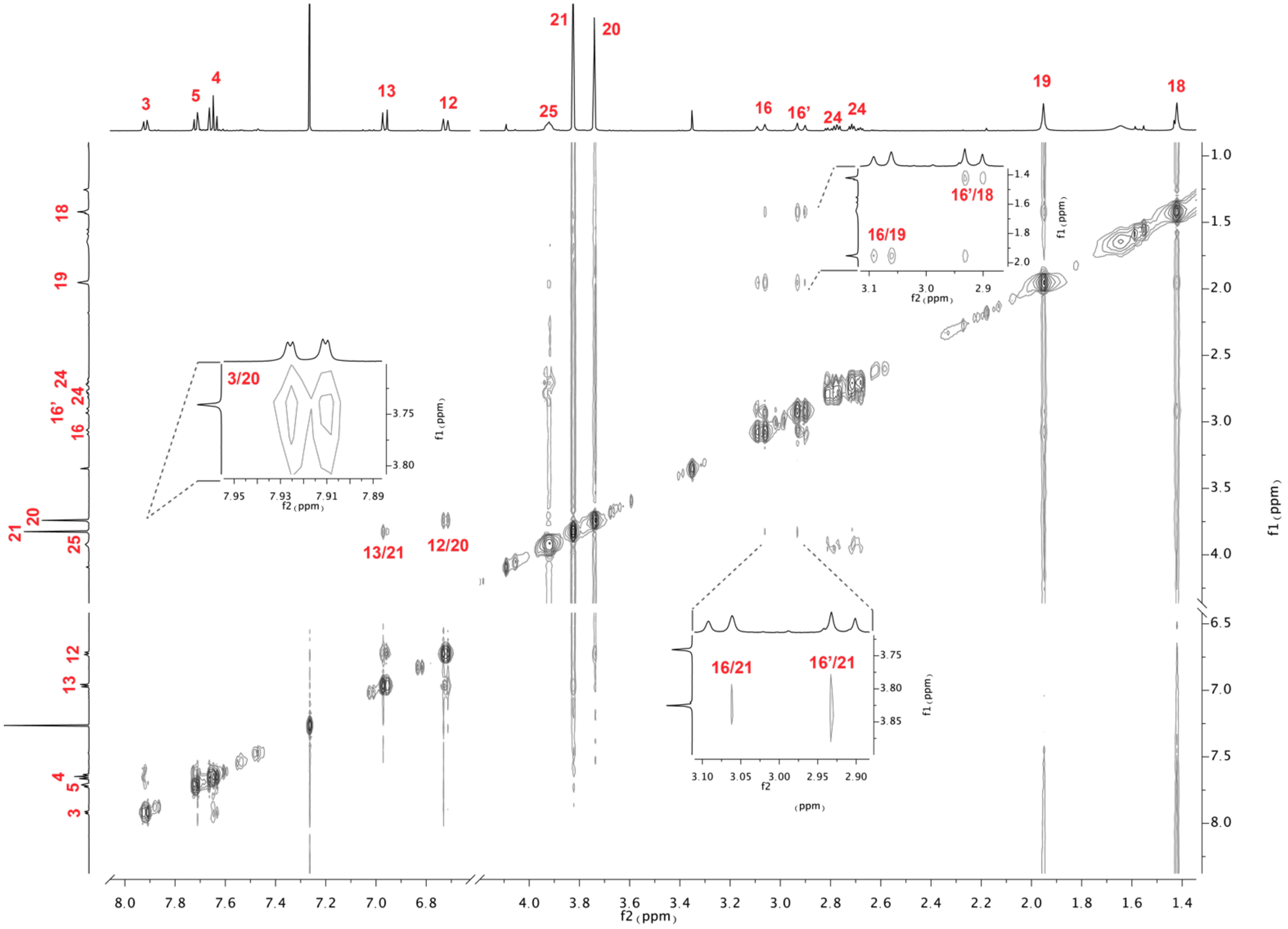

Figure S24. NOESY NMR Spectrum (500 MHz, $CDCl_3$, 0.3 s mixing time) of HTI **2-*E***. The cross-peaks between protons 12/20, 13/21, 16 /21, and 16'/21 were used to assign methoxy protons. The cross-peaks between protons 16/19 and 16'/19 were used to determine their relative assignments shown in **Figure S17.** The weak cross peak between 3 and 20 is consistent with the assignment of the E isomer. This correlation is also seen in related literature compounds.[7]

Additional Comments on the NMR Analysis of HTI Motors

The signals for the methylene protons in the five-membered ring differ significantly in the various motors; these differences have important structural implications. Each isomer of **1-4** and **14-16** gives an AB quartet. The doublet for the more shielded proton in the *E* isomer often extensively overlaps the doublet for the more shielded proton in the *Z* isomer (**1**, **3**, **4**, **14-16**); in **3**, the doublet for the more deshielded proton in the *E* isomer also extensively overlaps the doublet for the more deshielded proton in the *Z* isomer. In contrast, **5** and **23** give an AB quartet for the methylene protons in one isomer but just a singlet for the methylene protons in the other isomer, while **6** and **28** give a singlet for the methylene protons in each isomer. **5**, **23**, **6**, and **28** are sulfides, while **1-4** and **14-16** are chiral sulfoxides.

In sulfides **6** and **28**, there is no steric hindrance that would prevent the entire molecular framework from adopting a planar conformation. Neither compound has any chirality or helicity. This is analogous to the situation for a simple HTI switch, as shown in **Figure 1** (main text).

The presence of an AB quartet for a methylene group in one isomer of sulfide **5** and in one isomer of sulfide **23** (which differ only in the location of the Br substituent on the C_6H_3 ring) implies that the two methylene protons are inherently inequivalent; a planar framework is not present. Steric hindrance between the carbonyl group and proximate methoxy group in the *E* isomer is readily apparent. There is no steric hindrance between the sulfur atom and proximate methoxy group in the *Z* isomer. To alleviate congestion in the *E* isomer, the framework deviates from planarity. As a result, just a singlet is observed for the methylene protons in the *Z* isomer, while an AB quartet is observed for the methylene protons in the *E* isomer.

In **5** and **23**, the methylene singlet is clearly weaker than the methylene AB quartet. Thus, when other signals are resolved, they can easily be attributed to either the minor (*Z*) or major (*E*) isomer.

We note that a singlet would also be observed if the two methylene protons had extremely similar, but not identical, chemical shifts where the difference Δ in chemical shifts (in Hz) is much less than the geminal coupling constant ($\Delta \ll {}^2J$), as the outer components of this AB quartet would be so weak that they could easily be obscured by the baseline noise, while the inner components would severely overlap. This appears to be the case in analogous *Z* and *E* sulfides with Cl substituents, where crystal structures were also obtained.[7]

For a sulfoxide, steric hindrance is unavoidable between the carbonyl group and the proximate methoxy group in the *E* isomer or between the sulfoxide group and the proximate methoxy group in the *Z* isomer. To alleviate congestion in either isomer, the framework deviates from planarity, as shown in **Figure 1** (main text) and in references,[6,7] and adopts a helical structure. As a result, an AB quartet is observed for the methylene protons in each isomer. This has previously been observed in analogous *Z* and *E* sulfoxides with Cl substituents[7] and without Cl substituents[6] on the hemithioindigo aromatic ring; crystal structures were also obtained.[6,7]

References:

[1] G. Bertolini, F. Rossi, G. Valduga, G. Jori, J. Van Lier, *FEMS Microbiol. Lett.* **1990**, *71*, 149.

[2] M. Song, Y. Liu, X. Huang, S. Ding, Y. Wang, J. Shen, K. Zhu, *Nat. Microbiol.* **2020**, *5*, 1040.

[3] R. F. Epand, P. B. Savage, R. M. Epand, *Biochim. Biophys. Acta (BBA)-Biomembranes* **2007**, *1768*, 2500.

[4] E. Uhl, S. Thumser, P. Mayer, H. Dube, *Angew. Chemie Int. Ed.* **2018**, *57*, 11064.

[5] J. Su, Y. Qiu, K. Ma, Y. Yao, Z. Wang, X. Li, D. Zhang, Z. Tu, S. Jiang, *Tetrahedron* **2014**, *70*, 7763.

[6] M. Guentner, M. Schildhauer, S. Thumser, P. Mayer, D. Stephenson, P. J. Mayer, H. Dube, *Nat. Commun.* **2015**, *6*, 1.

[7] K. Hoffmann, P. Mayer, H. Dube, *Org. Biomol. Chem.* **2019**, *17*, 1979.
